# Membrane properties modulation by SanA: implications for xenobiotic resistance in *Salmonella* Typhimurium

**DOI:** 10.3389/fmicb.2023.1340143

**Published:** 2024-01-05

**Authors:** Adrianna Aleksandrowicz, Rafał Kolenda, Karolina Baraniewicz, Teresa L. M. Thurston, Jarosław Suchański, Krzysztof Grzymajlo

**Affiliations:** ^1^Department of Biochemistry and Molecular Biology, Faculty of Veterinary Medicine, Wrocław University of Environmental and Life Sciences, Wrocław, Poland; ^2^Department of Infectious Disease, Centre for Bacterial Resistance Biology, Imperial College London, London, United Kingdom

**Keywords:** antibiotics, *Salmonella*, inner membrane proteins, membrane permeability, SanA

## Abstract

**Introduction:**

Multidrug resistance in bacteria is a pressing concern, particularly among clinical isolates. Gram-negative bacteria like *Salmonella* employ various strategies, such as altering membrane properties, to resist treatment. Their two-membrane structure affects susceptibility to antibiotics, whereas specific proteins and the peptidoglycan layer maintain envelope integrity. Disruptions can compromise stability and resistance profile toward xenobiotics. In this study, we investigated the unexplored protein SanA’s role in modifying bacterial membranes, impacting antibiotic resistance, and intracellular replication within host cells.

**Methods:**

We generated a *sanA* deletion mutant and complemented it *in trans* to assess its biological function. High-throughput phenotypic profiling with Biolog Phenotype microarrays was conducted using 240 xenobiotics. Membrane properties and permeability were analyzed via cytochrome c binding, hexadecane adhesion, nile red, and ethidium bromide uptake assays, respectively. For intracellular replication analysis, primary bone marrow macrophages served as a host cells model.

**Results:**

Our findings demonstrated that the absence of *sanA* increased membrane permeability, hydrophilicity, and positive charge, resulting in enhanced resistance to certain antibiotics that target peptidoglycan synthesis. Furthermore, the *sanA* deletion mutant demonstrated enhanced replication rates within primary macrophages, highlighting its ability to evade the bactericidal effects of the immune system. Taking together, we provide valuable insights into a poorly known SanA protein, highlighting the complex interplay among bacterial genetics, membrane physiology, and antibiotic resistance, underscoring its significance in understanding *Salmonella* pathogenicity.

## Introduction

Salmonellosis is a major intestinal foodborne disease, globally affecting approximately 200 million people and causing 60,000 fatalities annually (Havelaar et al., 2015). Thus, it is an epidemiological threat and an impediment to socio-economic development worldwide. Considering the ability of *Salmonella* to survive in various conditions, adapt to new environments, and facultatively survive and replicate inside cells, the prevention and treatment of salmonellosis become quite challenging. This often results in an over-reliance on antibiotic therapy, particularly in developing countries (Ayukekbong et al., 2017). Predictive models suggest that by 2050, antimicrobial resistance (AMR) may result in 10 million annual fatalities worldwide (O’Neil, 2016). Hence, non-typhoidal *Salmonella* and *Salmonella ser.* Typhi have been categorized by the Center for Disease Control as “Serious Threats,” alongside other pathogens such as multidrug-resistant *Pseudomonas aeruginosa* and methicillin-resistant *Staphylococcus aureus* (Centers for Disease Control and Prevention, 2019).

Primarily, pathogenic bacteria have developed various defense mechanisms to withstand different environmental challenges, including exposure to xenobiotics. These mechanisms include (I) efflux pumps, which eliminate drugs from bacterial cells, thus reducing their concentration to non-toxic levels and causing loss of potency; (II) antibiotic inactivation by bacterial enzymes that alter or degrade antibiotic structures; (III) target site modification by spontaneous mutation and changing the chemical structure of their molecular targets; and (IV) preventing drug entry by altering bacterial membrane compositions (Alenazy, 2022). In all these processes the cell envelope, consisting of two lipid bilayers—inner membrane (IM) and outer membrane (OM) plays a critical role in protecting microorganisms from environmental stresses, as well as in cell viability and growth (Silhavy et al., 2010). The interdependence between OM and IM proteins is essential for preserving structural integrity of the bacterial cell envelope. Mutations in genes encoding IM proteins, such as *dedA* or *tat* may alter the membrane composition, potentially impacting membrane permeability and consequently resulting in antibiotic resistance (Boughner and Doerrler, 2012).

The outer membrane (OM) is a distinctive feature of gram-negative bacteria (Sun et al., 2022). It consists of an asymmetric lipid bilayer with the outer leaflet made of lipopolysaccharide (LPS) and the inner leaflet made of phospholipids (Nikaido, 2003; Sun et al., 2022). OM proteins can be classified as integral transmembrane β-barrel proteins (OMPs) and lipoproteins anchored in the inner leaflet (Malinverni and Silhavy, 2011). The most common lipoprotein is Lpp, which maintains periplasmic distance (Asmar and Collet, 2018). The OM’s essential role is to protect against hydrophobic molecules, and some OMPs act as channels for small or large molecules (Nikaido, 2003). It also provides mechanical strength to compensate for the thin cell wall (Nikaido, 2003). Changes in OM composition can lead to drug resistance, emphasizing its importance in antibiotic sensitivity. They may also influence the efficiency of phagocytosis and the intracellular survival of pathogens within macrophages, as a result of an increased resistance toward antimicrobial activity of these host cells (Matz and Jürgens, 2001; Lei et al., 2019).

In addition to the OM, the bacterial cytoplasm is surrounded by a phospholipid bilayer IM, regulating the movement of nutrients and ions in and out of the cytoplasm. It serves as the site for various metabolic processes such as energy production, lipid and peptidoglycan biosynthesis, protein transport, and translocation (Silhavy et al., 2010). IM proteins vary extensively, from peripheral and integral proteins to lipoproteins attached to the periplasmic side of the IM. Together, they constitute approximately 25% of the bacterial proteome (Papanastasiou et al., 2016). Despite their abundance, the functions of several IM proteins are still unclear. One such IM protein is SanA, which is potentially involved in envelope biogenesis (Rida et al., 1996).

*sanA* multi-copy expression suppresses the vancomycin sensitivity of *Escherichia coli* K-12 mutant, showing OM permeability defect which was confirmed using compounds such as Sodium Dodecyl Sulfate (SDS), Ethidium Bromide (EB), and the ingredients of MacConkey medium (Rida et al., 1996). The *S.* Typhimurium *sfiX* (*sanA* ortholog) deletion mutant is also vancomycin-sensitive, which suggests that SanA may constitute a barrier that denies antibiotic access to its site of action (Mouslim et al., 1998). Furthermore, our previous study demonstrated the role of SanA in the initial stages of *Salmonella* pathogenicity—invasion and adhesion (Kolenda et al., 2021). Although SanA is hypothesized to be potentially associated with bacterial cell wall synthesis or may function as an efflux pump activated during extreme conditions such as cold/heat shock or bile exposure, these roles lack conclusive establishment. Notably, the subcellular localization of the SanA protein has not been experimentally demonstrated, and prediction tools provide inconsistent results in this context. Furthermore, the influence of SanA on membrane properties remains unexplored, and the correlation between physicochemical changes in the envelope and their subsequent effects on antibiotic resistance has not been investigated.

Considering all these aspects, we hypothesized that *sanA* deletion affects the membrane permeability of *Salmonella* and induces shifts in the membrane’s physicochemical properties. These modifications are postulated to alter resistance to multiple antibiotic classes and enhance the bacterium’s ability to replicate within primary macrophages.

## Materials and methods

### Bacteria, plasmids, and growth conditions

All bacterial strains, plasmids, and primers used in this study are listed in Tables 1–3, respectively. All *Salmonella* strains used in this work were derived from the *Salmonella enterica* serovar Typhimurium 4/74. Unless stated otherwise, bacterial cultures were routinely grown at 37°C for 16 h under dynamic or static conditions in Lysogeny Broth (LB) or on agar plates, respectively. According to manufacturer’s recommendations, Biolog Universal Growth agar with 5% sheep blood was used to grow bacteria for the Biolog Phenotype Microarray. Mueller Hinton Broth (MHB) was used to measure antimicrobial activity. When necessary, ampicillin (Amp, 100 μg/mL) or kanamycin (Km, 50 μg/mL) was added. For *lac* promoter induction, isopropylthio-β-galactoside was added to a final concentration of 0.5 mM. Cell growth was monitored by measuring the optical density (OD) at 600 nm.

**Table 1:** Bacterial strains used in this study.

Strain	Relevant feature(s)	References
*S.* Typhimurium 4/74	Wild type (WT)	Dr Derek Pickard, Cambridge Institute for Therapeutic Immunology and Infectious Disease, University of Cambridge Department of Medicine, Cambridge, United Kingdom
*S.* Typhimurium 4/74 *ΔsanA*	*S.* Typhimurium 4/74 with *sanA* gene knockout (*ΔsanA*)	This study
*S.* Typhimurium 4/74 *ΔsanA*-pWSK29	*S.* Typhimurium 4/74 *ΔsanA* with pWSK29 empty plasmid	This study
*S.* Typhimurium 4/74 *ΔsanA-pWSK29*-*sanA*	*S.* Typhimurium 4/74 *ΔsanA* complemented with *sanA* carrying pWSK29 plasmid	This study
*E. coli* XL1-Blue	*recA1 endA1 gyrA96 thi-1 hsdR17 supE44 relA1 lac [F proAB lacIq ZM15 Tn10 (Tetr)]*	Wroclaw University of Environmental and Life Sciences, Department of Biochemistry and Molecular Biology collection

**Table 2:** Plasmids used in this study.

Plasmid	Relevant feature(s)	References
pKD46	pBAD λ redαβγ ts ori; AmpR	Datsenko and Wanner (2000)
pKD4	template plasmids for FRT-flanked kanamycin cassette	Datsenko and Wanner (2000)
pCP20	Helper plasmid FLP ts *ori*; AmpR, KanR	Cherepanov (1995)
pWSK29	Expression vector under the IPTG-induced lac promoter, AmpR	prof. dr hab. Dariusz Bartosik, Institute of Microbiology, Department of Bacterial Genetics, University of Warsaw
pWSK29-*sanA*	pWSK29 vector with *sanA* sequence insert, AmpR	This study

**Table 3:** Primers used in this study.

Name	Sequence (5′-3′)	References
sanA_del_for	ATGTTAAAGCGCGTGTTTTACAGCCTGTTGGTCCTGGTAGGCTTGCTGCTGTGTAGGCTGGAGCTGCTTC	Datsenko and Wanner (2000) and this study
sanA_del_rev	TCATTTCCCTTTTTTCTTTTCCAGTTCAAGCAATTGTTCCGGCGTAACTGCATATGAATATCCTCCTTAG	Datsenko and Wanner (2000) and this study
sanA_upstream_for	CGATACAAGGGAAATCATGCTG	This study
sanA_downstream_rev	TTCCAGGCCTCACGGAAG	This study
sanA_internal_rev	GCCCTGGATACGATAACGA	This study
O1646 k1	CAGTCATAGCCGAATAGCCT	Datsenko and Wanner (2000)
O1647 k2	CGGTGCCCTGAATGAACTGC	Datsenko and Wanner (2000)
sanA_XbaIpWSK_for	ACATTCTAGAAGGAGGACAGCTATGTTAAAGCGCGTGTTTTAC	This study
sanA_PstIpWSK_rev	ATCTGCAGTCATTTCCCTTTTTTCTTTTCCAG	This study
pWSK_T7_up	CTTCGCTATTACGCCAGCTG	This study

### Xenobiotics

The following xenobiotic stock solutions were used: 5,7-dichloro-8-hydroxyquinaldine [55 mg/mL in dimethyl sulfoxide (DMSO)]; bleomycin (2.56 mg/mL in sterile water); carbenicillin (20.5 mg/mL in sterile water); ceftriaxone (4 mg/mL in sterile water); cetylpyridinium chloride (164 mg/mL in sterile water); chlorhexidine acetate (25.6 mg/mL in ethanol); norfloxacin (0.8 mg/mL in DMSO); phosphomycin (40 mg/mL in sterile water); polymyxin B (1 mg/mL in sterile water); spectinomycin [100 mg/mL in DMSO:water (1:1)]; streptomycin (1 mg/mL in sterile water); sulfamonomethoxine (5 mg/mL in ethanol); thioridazine (16 mg/mL in DMSO); tobramycin (43.2 mg/mL in sterile water); umbelliferone (40 mg/mL in ethanol); and vancomycin (100 mg/mL in sterile water). All xenobiotic solutions were sterilized using 0.22 μm membrane filters and diluted in MHB medium to the appropriate concentration.

### Bioinformatic analysis

In the study, a comprehensive array of open-access bioinformatics tools was utilized to investigate the SanA in *S.* Typhimurium 4/74. The nucleotide and protein sequences of SanA (accession number CP002487.1: 2277943-2278662; protein ID: ADX17941.1) were extracted from the NCBI database in a FASTA format for the analyses. Orthologs of the SanA across various taxonomic groups were identified using the EggNOG tool, enabling the generation of a report on the prevalence of SanA in different taxa (Huerta-Cepas et al., 2019).

To compare the sequence similarity of SanA between *Salmonella* and *Escherichia coli*, BLASTN and BLASTP were employed for nucleotide and protein sequence analysis, respectively. Furthermore, the investigation included an in-depth analysis of subcellular protein localization. For this purpose, several tools such as Phobius, SignalP-5.0, PsortB, THMM 2.0, and TMpred were applied (Krogh et al., 2001; Käll et al., 2007; Yu et al., 2010; Petersen et al., 2011; Finn et al., 2014).

Subsequently, Phyre2 was employed to conduct a comparative analysis of SanA against homologous sequences available in the database (Kelley et al., 2015). Additionally, the Panther classification system was utilized to categorize the protein and predict its function, providing insights into its potential biological roles and activities (Thomas et al., 2003).

### Bacterial mutant construction

*Salmonella* Typhimurium 4/74 with *sanA* gene knockout was generated using the protocol described by Datsenko and Wanner (2000), with slight modifications (Datsenko and Wanner, 2000). Initially, electrocompetent cells of the wild-type (WT) strain were transformed with a Red recombinase-carrying plasmid pKD46. The positive clones were further transformed with a kanamycin cassette flanked by FRT sites, which was obtained via polymerase chain reaction (PCR) using primers sanA_del_for and sanA_del_rev on the pKD4 template, and selected on LB agar plates containing kanamycin at 37°C. The FRT flippase present on the pCP20 plasmid was then utilized to eliminate the kanamycin cassette. Colony PCR using locus-specific primer pairs, sanA_upstream_for, sanA_upstream_rev, and sanA_internal_rev was performed to confirm the correct integration and removal of the marker cassette. To determine whether the newly-created strain differed in growth rate or morphology from the parental isolate, growth curves were determined, and acridine orange staining was used to examine them using a fluorescence microscope. Furthermore, the absence of any unintended mutations was confirmed using Next Generation Sequencing.

### Cloning of *sanA* into pWSK29 plasmid and mutation complementation

The *sanA* from *S.* Typhimurium 4/74 was amplified using sanA_XbaIpWSK_for, sanA_PstIpWSK_rev primers, and Phusion polymerase (Thermo), according to the manufacturer’s protocol. The PCR products were purified using the GeneJET PCR purification kit (Thermo) and the plasmid DNA was isolated using the GeneJET Plasmid Miniprep Kit (Thermo). To insert *sanA* into the pWSK29 plasmid, the gene was cloned into the XbaI/PstI digestion sites using the classical ligation method. DNA sequence of the insert was confirmed using colony PCR via the use of a specific primer pair sanA_internal_rev and pWSK_T7_up, and Sanger sequencing. For complementing the deletion mutant, electrocompetent *S.* Typhimurium 4/74 *ΔsanA* was transformed with a plasmid carrying complementing gene as well as pWSK29 vector plasmid alone (without insert) as a control. All clones were analyzed in positive selection on LB agar with ampicillin.

### Growth curve determination

To determine the growth curves of *Salmonella* strains, a single bacterial colony of each isolate was inoculated in LB and incubated overnight at 37°C with shaking (180 rpm). The resulting cultures were diluted to OD_600_ = 0.05 using LB and incubated until the early logarithmic growth phase (OD_600_ = 0.5, 37°C, 220 rpm). Each culture was then centrifuged, washed, and suspended in 0.9% NaCl solution. The OD_600_ values were measured, and the cultures were diluted in LB to obtain 5 × 10^6^ CFU/mL bacterial suspensions. For determining the antimicrobial effect of vancomycin and bile salts, the assay was performed in LB or MHB medium, respectively with 0%–15% bile salts and 0–500 μg/mL vancomycin. The samples were then applied to a polystyrene or polypropylene 96-well plate in triplicate and incubated in a spectrophotometer (Tecan) at 37°C with measurements taken at 15-min intervals for 16 h, with shaking before each reading. The experiment was performed in at least three independent biological replicates, and dilution series on LB agar were prepared to verify initial bacterial concentrations.

### Phenotype microarray analysis

The susceptibility of mutant and the parental strain to 240 chemical compounds was determined in three independent experiments using the Phenotype MicroArray (PM) PM11-PM20 (Biolog), as described in a previous study (Shea et al., 2012). Briefly, strains were grown overnight on Biolog Universal Growth agar with 5% sheep blood at 37°C, colonies were then picked using a sterile cotton swab and suspended in 15 mL of 1× inoculation fluid (IF-0a GN/GP Base, Biolog). The cell density was adjusted to 85% transmittance (T) using a Biolog turbidimeter. The inoculation fluid for PM11-20 was prepared by mixing 100 mL of IF-10a GN Base (1.2X; Biolog), 1.2 mL of Biolog Redox Dye A (100X; Biolog), 0.6 mL of cell suspension at 85% T, and sterile water to reach a final volume of 120 mL. The mixture was then inoculated in the PM plates (100 μL per well) and color development was monitored every 15 min for 48 h at 37°C using an Omnilog reader (Biolog). The kinetic curves of both strains were compared using Omnilog-PM software to identify the phenotypes. Raw data were obtained for 10 plates, which included 240 antibiotics arranged as a dilution series across four wells (960 wells in total). Data were recorded in the RA format and filtered using differences of average height with standard thresholds to identify statistically significant differences using Student’s t-test (Guard-Bouldin et al., 2007). The reproducibility of our results was ensured by excluding any differences greater than 50 Omnilog units between biological replicates from the analysis (see Supplementary Table S1).

### Antimicrobial susceptibility testing

As the manufacturer of PM plates (Biolog) does not disclose the concentrations of compounds in their plates, on the basis of available literature, we selected 8 different concentrations for all compounds by making two-fold dilutions. Polypropylene and polystyrene plates were utilized for cationic and anionic compounds, respectively. Bacterial strains were incubated in LB for 16 h at 37°C, 180 rpm. Further, the OD_600_ was determined, and bacteria were diluted in MHB to get a total density of 10^6^ CFU/mL. The suspensions were aliquoted at 50 μL per well into previously prepared 96-well plates and incubated at 37°C. After 16 h, OD_600_ of each well was measured with using the Tecan microplate reader (Spark®). MHB without xenobiotics serves as a positive control of growth. At least three technical and biological repetitions were performed for each strain. All xenobiotics and tested concentration ranges are listed in Table 4. Antibiotic susceptibility testing results determine the fold change of OD_600_ values between the *ΔsanA* and WT strain at concentrations showing a significant difference. The standard error of mean (SEM) was calculated using the standard deviation of the sample and the square root of the sample size.

**Table 4:** Antimicrobial concentration ranges included in the study.

Compound	Concentration range (μg/mL)	Solvent
5,7-Dichloro-8-hydroxyquinaldine	0.100–27.700	DMSO
Bleomycin	0.025–6.400	Water
Carbenicillin	0.400–102.400	Water
Ceftriaxone	0.002–0.4192	Water
Cetylpyridinium chloride	3.250–832	Water
Chlorhexidine acetate	0.250–64	Ethanol
Norfloxacin	0.010–2	DMSO
Phosphomycin	0.200–39.600	Water
Polymyxin B	0.020–5	Water
Spectinomycin	1.953–500	DMSO:water (1:1)
Streptomycin	0.390–100	Water
Sulfamonomethoxine	1–248	Ethanol
Thioridazine	6.250–1,600	DMSO
Tobramycin	0.084–21.600	Water
Umbelliferone	7.810–2000	Ethanol
Vancomycin	1.953–500	Water

### Membrane permeability

The OM permeability was investigated by utilizing the influx of either the cationic Ethidium Bromide (EB) or the neutral Nile Red dye (NR; Murata et al., 2007; Viau et al., 2011). Overnight bacterial cultures were diluted to OD_600_ = 0.05 using LB, incubated until early stationary growth phase (OD_600_ = 2.0, 37°C, 220 rpm) and rinsed twice with assay buffer (50 mM KH_2_PO_4_, 137 mM NaCl, pH 7.0). To perform dye uptake assays, the proton motive force inhibitor carbonyl cyanide-m-chlorophenylhydrazone (CCCP) was added at a final concentration of 10 μM. Fluorescence was measured for 30 min at 1-min intervals using a Tecan microplate reader (Spark®) immediately upon mixing cells (final OD_600_ = 0.2) with EB at a final concentration of 6 μM (with excitation at 545 nm and emission at 600 nm) or NR at a final concentration of 2 μM (with excitation at 540 nm and emission at 630 nm). Membrane permeability was measured in at least three independent experiments. According to Murata et al., the dye uptake rates of different strains varied between experiments, but the pattern of dye uptake remained consistent across repetitions (Murata et al., 2007).

### Hexadecane adhesion assay

Bacterial surface hydrophobicity was determined using the hexadecane adhesion assay (Oguri et al., 2016). Overnight bacterial cultures were diluted to OD_600_ = 0.05 using LB and incubated until early stationary growth phase (OD_600_ = 2.0, 37°C, 220 rpm). Subsequently, the cultures were harvested, washed twice with phosphate buffered saline (PBS), and resuspended in 1 mL of PBS. Following this, 100 μL of cells were diluted 10× in PBS and the OD_600_ was measured (C_0_). Next, 900 μL of the cell suspension was mixed with 200 μL of hexadecane (Merck Millipore), vortexed for 1 min, and left undisturbed at room temperature until the phases separated. Cell samples (100 μL) from the lower, aqueous phase were then diluted in 900 μL PBS and OD_600_ was measured (C_H_). The percentage of hexadecane adherence was determined in three independent experiments, using the following formula: % hexadecane adherence = [(C_0_ – C_H_)/C_0_] × 100.

### Cytochrome c binding assay

The cytochrome c binding assay was performed as described previously, with minor modifications (Kristian et al., 2005). Briefly, overnight bacterial cultures were diluted to OD_600_ = 0.05 using LB and incubated until early stationary growth phase (OD_600_ = 2.0, 37°C, 220 rpm). Bacteria were then collected, washed twice in 3-(N-morpholino)propanesulfonic acid (MOPS) buffer (20 mM, pH 7.4), and adjusted to a final OD_600_ = 7.0 in the same buffer. Next, bacteria were mixed with cytochrome c (Merck Millipore) to a final concentration of 0.5 mg/mL, incubated for 10 min at room temperature, and centrifuged at 18,000 × g for 6 min. Cytochrome c without bacteria in the same buffer was also incubated as a negative control. The cytochrome c contents in the supernatants were measured at the absorption maximum of the prosthetic group (530 nm). The percentage of bound cytochrome c was calculated from three independent experiments, each performed in triplicate.

### Bone marrow-derived macrophages derivation and culture

The isolation of primary bone marrow-derived macrophages (pBMDMs) was performed in accordance with a UK Home Office Project License in a Home Office designated facility, as previously described (Thurston et al., 2016; Bailey et al., 2020). Briefly, bone marrow was obtained from 6 to 8 week-old female C57BL/6 mice (Charles River) by flushing the tibias and femurs. The collected cells were then added to non-tissue culture-treated petri plates at a concentration of 3 × 10^6^ cells per plate in 8 mL Dulbecco’s modified Eagle’s medium (DMEM) with high glucose supplemented with 20% (v/v) L929-MCSF supernatant, 10% (v/v) fetal bovine serum (FBS), 10 mM HEPES, 1 mM sodium pyruvate, 0.05 mM β-mercaptoethanol, and 100 U/mL penicillin/streptomycin. After 3–4 days, 10 mL fresh medium was supplemented and the differentiated BMDMs were harvested on day 7. The macrophages were then seeded into 24-well tissue culture-treated plates at a concentration of 2 × 10^5^ macrophages per well and infected the following day with DMEM media supplemented with the above concentrations of FBS, HEPES, sodium pyruvate, and β-mercaptoethanol but without antibiotics.

### Infection assay

The macrophage monolayer was infected with stationary phase bacteria opsonized in mouse serum for 20 min at room temperature at a multiplicity of infection of 10:1. To synchronize the infection, the culture plates were centrifuged for 5 min at 165 × g, followed by a 30-min incubation at 37°C (5% CO_2_). Fresh DMEM supplemented with 100 μg/mL gentamicin (Gm) was added to kill extracellular bacteria, and the macrophage monolayers were incubated with added Gm for 90 min (Monack et al., 1996). After washing with DMEM, the monolayers were lysed in 1% Triton X-100 and diluted with PBS. Dilutions of the suspension were then plated on LB agar to quantify the number of viable bacteria. To evaluate intracellular growth, the medium containing 100 μg/mL Gm was replaced with DMEM supplemented with 10 μg/mL Gm, and parallel cell cultures were examined for viable bacteria 24 h following infection (Monack et al., 1996).

### Statistical analyses

Statistical analyses were performed using GraphPad Prism (GraphPad Software, Inc., La Jolla, CA, United States). The Shapiro–Wilk normality test was used to determine data distribution. Depending on the data distribution, either Student’s *t*-test, two-way ANOVA analysis of variance with Tukey’s correction, or the Kruskal–Wallis test with Dunn’s multiple comparison post-hoc test was used. For each condition, data were collected from at least three independent experiments. *p* ≤ 0.05 was considered statistically significant. The results were presented as mean ± SEM. The symbols ^*^p < 0.05; ^**^p < 0.01; ^***^p < 0.001; and ^****^p < 0.0001 were used to indicate significance levels.

## Results

### Insights into SanA protein: analysis using bioinformatic tools

According to our analysis of the eggNOG database, we found that the *sanA* is present across a diverse range of bacterial taxa. Specifically, the gene was identified in both, gram-negative and gram-positive bacteria with a prevalence of 44.1% in species classified under *Gammaproteobacteria*, 17.2% of *Bacteroidetes*, 18.9% of *Actinobacteridae*, 5.72% of *Clostridia*, and 3.37% of *Spirochaetia*. Additionally, all SanA homologs have an unknown domain, DUF218. We performed a BLAST comparison and found that SanA in *E. coli* and *Salmonella* share 94% identity, with an estimated 97% amino acids having identical or similar chemical properties, suggesting its high conservative among bacteria.

The predictions for the subcellular localization were inconsistent: Phobius suggested a location outside the cytoplasm, SignalIP-5.0 detected no signal peptide implying a cytoplasmic protein, PsortB indicated a cytoplasmic position, while both THMM 2.0 and TMpred identified a transmembrane domain. Since proteins with similar structures can have similar functions, we elucidated the potential function of SanA by predicting its structure using Phyre2 and comparing it to homologous sequences. Our findings suggested that SanA shares structural similarities with the YdcF protein of *E. coli*, which is involved in binding S-adenosyl-L-methionine; transferases (5-methyltetrahydrofolate homocysteine s-methyltransferase); OmpA like protein; peptide binding protein; membrane protein, and structural protein. Moreover, the Panther classification system revealed that SanA is an IM protein with potential permease activity and is classified into the transporters group.

### Impact of *sanA* knockout on resistance profile toward vancomycin and bile salts

**Figure 1:**
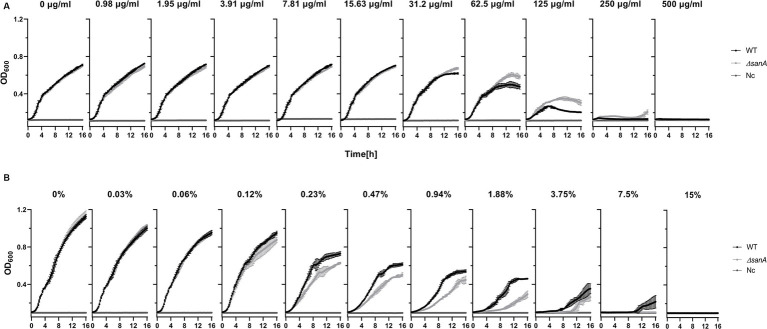
Growth curve of *Salmonella* Typhimurium 4/74 and its deletion mutant *ΔsanA* strains in the presence of: **(A)** vancomycin in the concentration range between 0 and 500 μg/mL, **(B)** bile salts in the concentration range between 0% and 15%. The data comprises of median values and median absolute deviation (MAD) of at least three separate experiments in triplicate. Nc represents a negative control: medium containing xenobiotics but lacking bacteria.

As SanA is known as a vancomycin exclusion protein, the first stage of our investigation incorporated analysis of the WT and *ΔsanA* mutant bacteria growth in the presence of vancomycin or bile salts. Surprisingly, the mutant strain showed higher resistance to vancomycin than the WT (Figure 1A). While the WT grew only up to 125 μg/mL vancomycin, *sanA* deletion allowed the strain to grow up to a concentration of 250 μg/mL vancomycin. Moreover, a significant difference in the optical density between the two strains was observed in the presence of 62.5 and 125 μg/mL vancomycin, and the highest contrast was visible in the stationary growth phase—after 10 h (Figure 1A). Additionally, the two strains displayed contrasting growth patterns in the presence of bile salts. The deletion mutant strain demonstrated decreased resistance with growth up to only 3.75% bile salts compared to the WT, which grew up to 7.5% bile salts. Significant growth variations were also noted between the two strains at bile salt concentrations of 0.47%–1.88% (Figure 1B). The phenotypic parallels observed between the strain complemented with *sanA* and the one transformed with the empty pWSK29 plasmid further underscore the function of SanA in these resistance profiles (Supplementary Figure S2).

### High-throughput analysis of xenobiotic resistance phenotype

WT and *ΔsanA* were further characterized using Biolog (Biolog®) phenotypic arrays to investigate potential gene knockout-induced changes in resistance profiles. The arrays featured various compounds with some known antimicrobials included on plates PM11a to PM20. Prior to testing, no significant differences in growth kinetics were observed among the strains (Supplementary Figure S1).

Our results revealed distinct resistance patterns for more than 20% (49/240) of the analyzed compounds with different mechanisms of action (*p* < 0.05; Table 5). The *ΔsanA* strain exhibited improved growth in the presence of approximately 35% (17/49) of these 49 agents, grouped mostly as cell wall-and DNA-associated antibiotics. The same strain demonstrated lower resistance to folate antagonists (sulfonamides), membrane-targeting antibiotics, and DNA and protein-associated antibiotics (fluoroquinolones, glycopeptides, nucleic acid analogs; Table 5).

**Table 5:** Schematic representation of statistically significant data obtained from PM (from PM11a to PM20) analyses.

Compound	Difference	Mode of action
**PHENOTYPE LOST (lower optical density after *sanA* knockout) BY *Salmonella* Typhimurium *ΔsanA* RELATIVE TO *S.* Typhimurium WT**		
Umbelliferone	**−67.59**	**DNA intercalator**
Thioridazine	**−63.98**	**Membrane, phenothiazine, efflux pump inhibitor, anti-psychotic**
Cetylpyridinium chloride	**−62.62**	**Membrane, detergent, cationic**
Norfloxacin	**−58.49**	**DNA topoisomerase, fluoroquinolone**
DL-Serine hydroxamate	−51.68	tRNA synthetase
Pentachlorophenol	−51.56	Respiration, ionophore, H+
Chlorhexidine diacetate	**−51.28**	**Membrane, electron transport**
5,7-Dichloro-8-hydroxyquinaldine	**−51.18**	**RNA synthesis inhibitor, interference with transcription**
Sulfamonomethoxine	**−50.87**	**Folate antagonist, sulfonamide**
Ethionamide	−50.21	Anti-tuberculosic
Bleomycin	**−43.09**	**Inhibition DNA replication, oxidation, glycopeptide**
Sulfamethazine	−42.3	Folate antagonist, PABA analog, sulfonamide
Fusaric acid	−40.02	chelator, lipophilic
Trifluorothymidine	−35.2	Nucleic acid analog, pyrimidine, DNA synthesis
Sulfadiazine	−34.56	Folate antagonist, PABA analog, sulfonamide
Sulfisoxazole	−33.73	Folate antagonist, PABA analog, sulfonamide
1-Hydroxypyridine-2-thione (pyrithione)	−32.27	Biofilm inhibitor, chelator, anti-fungal
Sorbic acid	−31.04	Respiration, ionophore, H+, preservative
Sulfanilamide	−30.64	Folate antagonist, PABA analog, sulfonamide
Nitrofurantoin	−29.41	Nitro compound, oxidizing agent, DNA damage
Vancomycin	**−29.24**	**Wall, glycopeptide**
Tetraethylthiuram disulfide	−26.81	Nucleic acid inhibitor, purine
trans-Cinnamic acid	−26.7	Respiration, ionophore, H+
Sulfachloropyridazine	−23.02	Folate antagonist, PABA analog, sulfonamide
Phosphomycin	**−22.85**	**Wall, phosphonic**
5-Fluorouracil	−21.73	Nucleic acid analog, pyrimidine
5-Azacytidine	−21.54	DNA methylation, methyltransferase inhibitor
Polymyxin B	**−20.89**	**Membrane, cyclic peptide, polymyxin**
Ruthenium red	−19.28	Respiration, mitochondrial Ca++ porter
Penimepicycline	−15.34	Protein synthesis, 30S ribosomal subunit, tetracycline
Diamide	−11.06	Oxidizes sulfhydryls, depletes glutathione
Captan	−3.28	Fungicide, carbamate
**PHENOTYPE GAINED (higher optical density after *sanA* knockout) BY *S.* Typhimurium *ΔsanA* RELATIVE TO *S.* Typhimurium WT**		
Menadione, sodium bisulfite	4.87	Respiration, uncoupler
Spectinomycin	**10.16**	**Protein synthesis, 30S ribosomal subunit, aminoglycoside**
Poly-L-lysine	10.67	Membrane, detergent, cationic
2-Phenylphenol	13.67	DNA intercalator, preservative
Streptomycin	**15.65**	**Protein synthesis, 30S ribosomal subunit, aminoglycoside**
Cytosine-1-beta-D-arabinofuranoside	17.12	Nucleic acid analog, pyrimidine
Chromium (III) chloride	19.38	Toxic cation
Hydroxylamine	20.87	DNA damage, mutagen, antifolate (inhibits thymine and methionine synthesis)
3,5-Diamino-1,2,4-triazole (Guanazole)	22.31	Ribonucleotide DP reductase inhibitor, aromatic amine
Thiosalicylate	22.44	Biofilm inhibitor, anti-capsule agent, chelator, prostaglandin syntetase inhibitor
Phenyl-methylsulfonyl-fluoride (PMSF)	24.9	Protease inhibitor, serine
Chelerythrine chloride	26.82	Protein kinase C inhibitor
Myricetin	28.82	DNA & RNA synthesis, polymerase inhibitor
Cesium chloride	29.38	Toxic cation
Ceftriaxone	**31.69**	**Wall, cephalosporin**
Tobramycin	**35.91**	**Protein synthesis, 30S ribosomal subunit, aminoglycoside**
Carbenicillin	**57.92**	**Wall, lactam**

Data from Omnilog were recorded in the RA format and filtered using differences of average height values between *S.* Typhimurium 4/74 WT and *ΔsanA* to identify statistically significant differences. Phenotype lost indicates lower optical density after *sanA* knockout (negative values in the Table), and gained—higher optical density as a result of mutation (positive values in the Table). The bold text indicates agents which were chosen for further analysis with the use of microbroth dilution assay.

**Figure 2:**
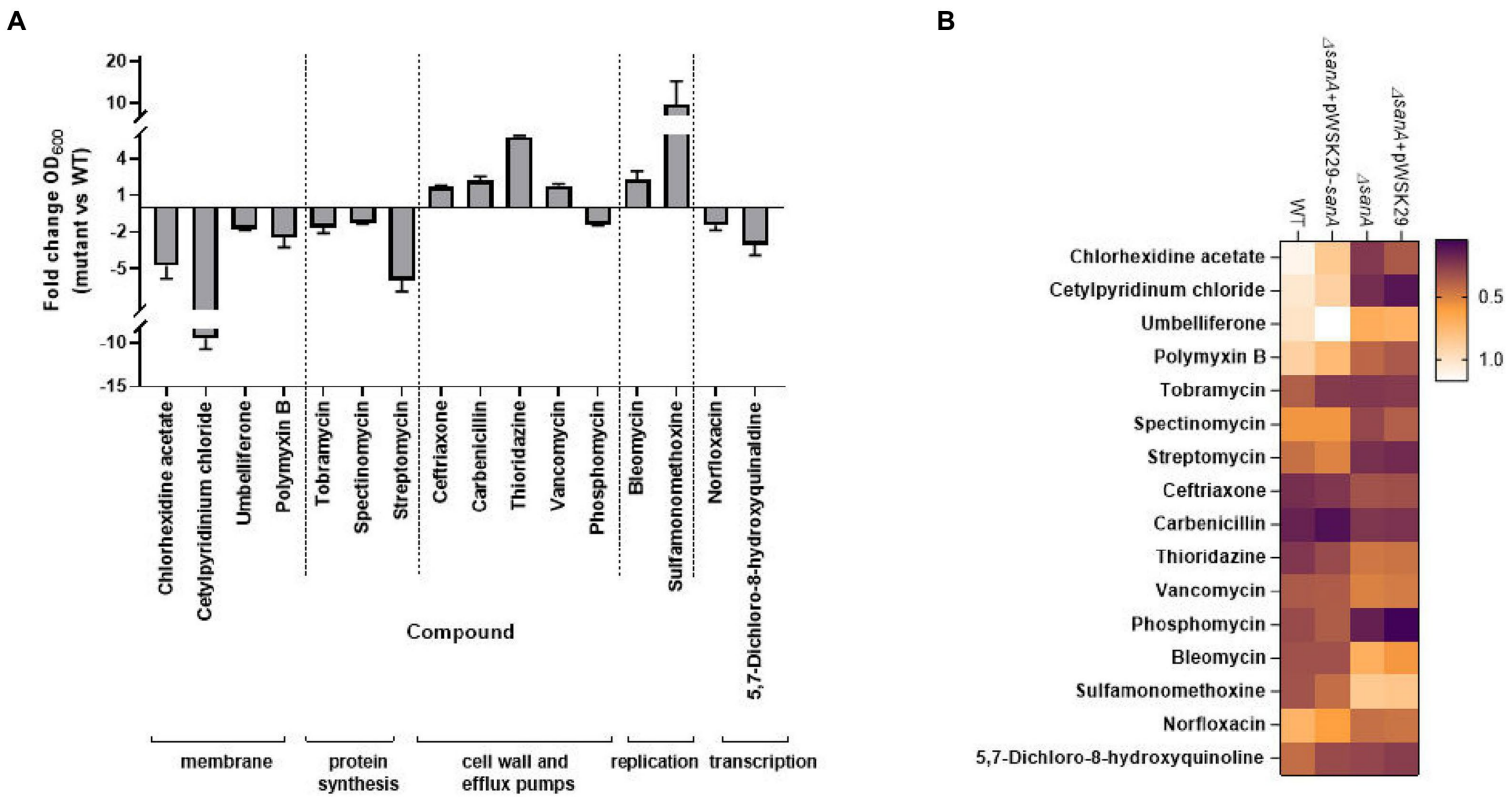
Antibiotic susceptibility testing represented by **(A)** bar chart with fold change OD_600_ of *Salmonella* Typhimurium 4/74 deletion mutant *ΔsanA* and WT after 16 h incubation in MHB medium with the presence of indicated agents. Data shown are means and SEM for at least three independent experiments **(B)** heatmap of OD_600_ of *S.* Typhimurium 4/74, its deletion mutant *ΔsanA* and *ΔsanA* transformed with empty pWSK29 plasmid or vector with *sanA* after 16 h incubation in MHB medium with the presence of indicated agents. Brown represents low relative growth in a given condition while white represents high growth.

Considering the known adjustments of the PM plates method with MIC measurements, we cross-checked PM results using a microbroth dilution assay. We focused on 16 compounds with the most significant differences between strains, particularly those targeting the membrane and cell wall (Table 5). The results, showing fold changes at OD_600_ between the *ΔsanA* and WT at specific concentrations (Supplementary Figure S3), led to further investigation using complemented strains (Figure 2B).

In the presence of 16 different agents, the *ΔsanA* strain exhibited reduced resistance to ten xenobiotics but displayed increased resistance to six others (Figure 2A). We grouped these agents into the following five categories: (1) membrane, (2) protein synthesis, (3) cell wall and efflux pumps, (4) replication, and (5) transcription (Figure 2A). The *sanA* knockout resulted in compromised growth with certain membrane-associated xenobiotics such as chlorhexidine acetate (2 μg/mL; *p = 0.0003*), cetylpyridinium chloride (6.5 μg/mL; *p = 0.0093*), umbelliferone (500 μg/mL; *p = 0.0465*), and polymyxin B (0.625 μg/mL; *p = 0.0205*). Notably, reintroducing *sanA* restored the resistance pattern on these agents to resemble WT bacteria (Figure 2B).

The *ΔsanA* exhibited lower resistance to agents targeting protein synthesis, such as tobramycin (0.675 μg/mL; *p = 0.0070*), streptomycin (100 μg/mL; *p = 0.0152*), and spectinomycin (31.250 μg/mL; *p = 0.0359*), as well as those targeting transcription, such as norfloxacin (0.010 μg/mL; *p = 0.0052*) and 5,7-dichloro-8-hydroxyquinaldine (3.5 μg/mL; *p = 0.0065*; Figure 2A). Notably, the *ΔsanA* strain showed a different resistance pattern to protein synthesis agents than that suggested by PM. However, when the mutation was complemented, the resistance pattern was mostly attributed to the *sanA* deletion, except for tobramycin (0.675 μg/mL; *p = 0.9998*; Figure 2B).

As anticipated, *sanA* deletion resulted in greater resistance to cell wall and efflux pumps associated compounds, like ceftriaxone (0.002 μg/mL; *p = 0.016*), vancomycin (125 μg/mL; *p = 0.0044*), carbenicillin (3.200 μg/mL; *p = 0.0281*), and thioridazine (1,600 μg/mL; *p = 0.027*). Surprisingly, this strain showed reduced resistance to phosphomycin (9.900 μg/mL; *p = 0.0483*; Figure 2A). Complementation mostly restored WT phenotypes, with the exception of thioridazine (1,600 μg/mL; *p = 0.2196*). In contrast to the PM data, *ΔsanA* demonstrated reduced susceptibility to replication agents, such as bleomycin (0.400 μg/mL; *p = 0.0104*) and sulfamonomethoxine (248 μg/mL; *p = 0.0432*). This phenotype was further validated by complementing *sanA,* highlighting its critical role in this phenotype (Figure 2B).

### SanA is responsible for membrane integrity

The impact of *sanA* on resistance to vancomycin along with other antimicrobial agents suggests a general effect on membrane integrity rather than specific vancomycin sensitivity. Thus, OM permeability was determined by measuring influx of the cationic dye, EB or the neutral dye, NR. Dye uptake assays were performed in the presence of CCCP, which enables the inward transport of H^+^ across lipid membranes. Therefore, it prevents the efflux of compound by active pumps, so that only passive permeability is measured. In the experiment without CCCP, a minimal increase in dye uptake was noted, suggesting that the increased retention of EtBr in the *ΔsanA* is not due to pump inactivation, but increased membrane permeability (Supplementary Figure S4).

**Figure 3:**
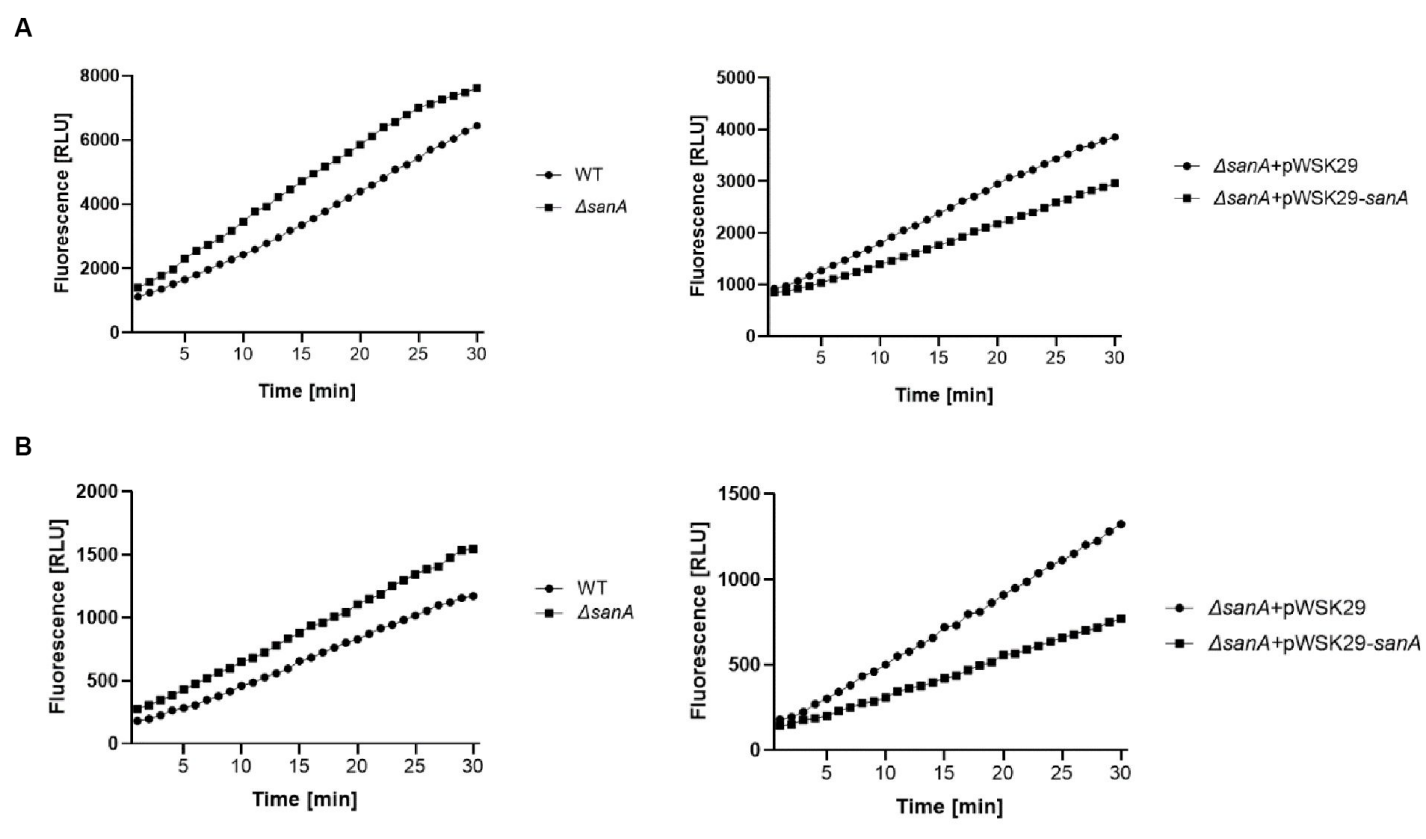
Outer membrane permeability of *Salmonella* Typhimurium 4/74, its deletion mutant *ΔsanA* and *ΔsanA* transformed with empty pWSK29 plasmid or vector with *sanA* **(A)** cationic dye Ethidium Bromide or **(B)** neutral dye Nile Red. The assay was conducted in the presence of CCCP to prevent the efflux of compound by active pump to measure only passive permeability. Data shown are representative of at least three independent experiments with similar results.

When CCCP was present, the *ΔsanA* bacteria demonstrated a notably higher OM permeability baseline than the WT for both EB and NR, with a remarkably increased rate of dye uptake observed particularly after approximately 10 min of assay initiation (Figure 3; Supplementary Figure S5). Importantly, complementation of the mutation restored the WT phenotype for NR and further reduced the permeability for EB, strongly suggesting that the observed phenotype was primarily due to *sanA* deletion. Moreover, the permeabilities differed between WT and *ΔsanA*, as well as between *ΔsanA*-pWSK29 and its complemented *ΔsanA*-pWSK29-*sanA* counterpart, consistently throughout the assay (Figure 3).

### SanA knockout decreases hydrophobicity and negative charge of the bacterial membrane

Considering the distinct resistance phenotype observed for different groups of xenobiotics, we hypothesized that the surface charges and hydrophobicity of bacterial cells could be contributing factors. To investigate potential alterations in the surface properties of the *ΔsanA*, we performed the following two assays: (1) determining surface charges by evaluating the binding of the cationic protein cytochrome c to bacterial cells, and (2) determining surface hydrophobicity by measuring the adherence of cells to the hydrophobic solvent hexadecane.

**Figure 4:**
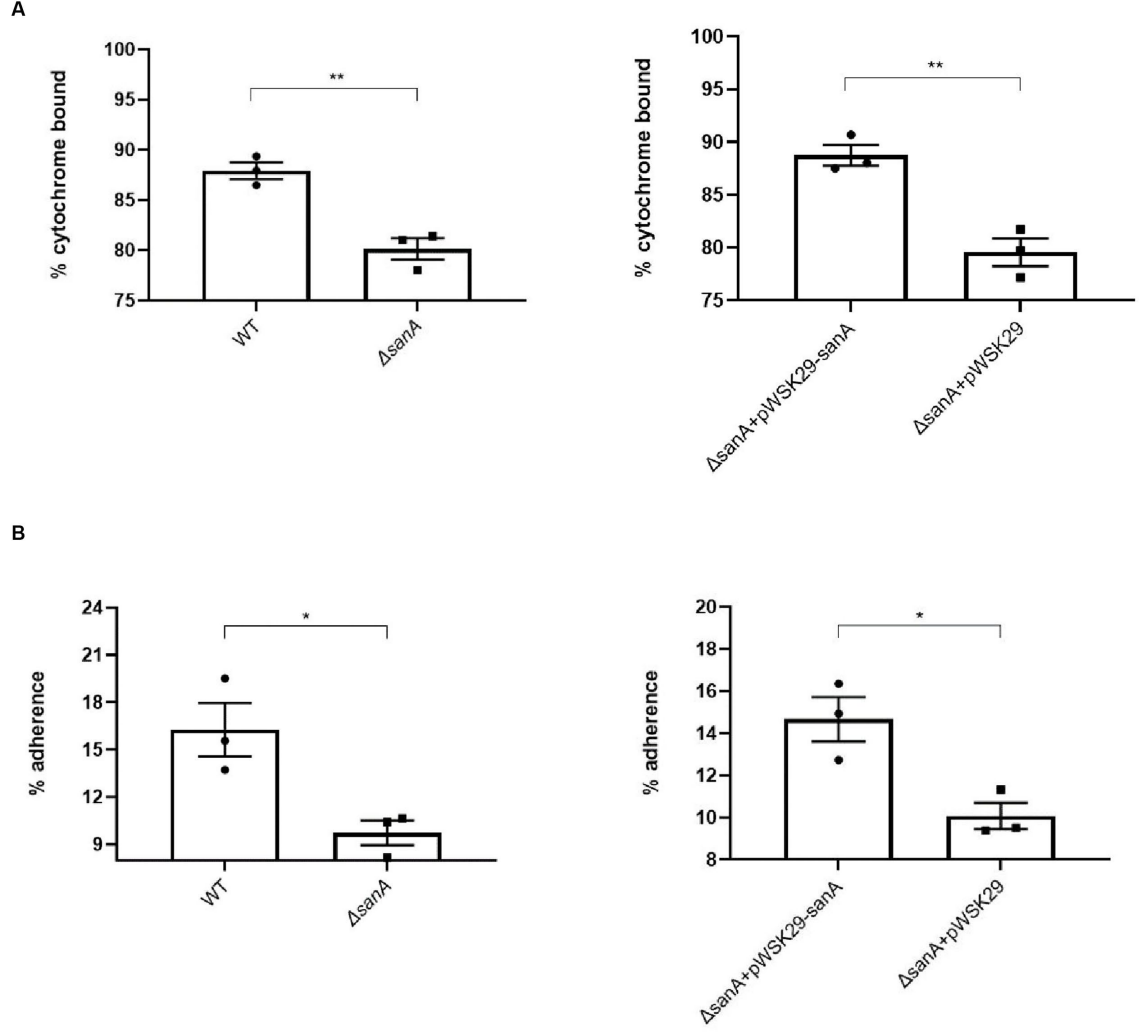
Physicochemical properties of the cell surfaces of *Salmonella* Typhimurium 4/74, its deletion mutant *ΔsanA*, and *ΔsanA* transformed with empty pWSK29 plasmid or vector with *sanA* **(A)** Surface charges were examined by a cytochrome c binding assay. **(B)** Hydrophobicities of cell surfaces were examined by a hexadecane adhesion assay. Data shown are means and SEM for at least three independent experiments. Statistical significance was determined by Student’s *t* test (^*^p < 0.05; ^**^p < 0.01).

The *ΔsanA* cells displayed significantly lower affinity to cytochrome c (80%) than that of WT cells (90%; *p = 0.0046*; Figure 4A). Additionally, approximately 10% of *ΔsanA* cells adhered to hexadecane, in contrast to approximately 16% of WT cells (*p = 0.0256*; Figure 4B). This suggests a decrease in the negative charge and hydrophobicity on the cell surface of *ΔsanA* mutant, respectively. Moreover, all these changes were attributed entirely to *sanA*, as introduction of *sanA* to the deletion mutant restored the WT phenotype (*p = 0.0199*; *p = 0.005*).

### SanA influences the replication of *Salmonella* Typhimurium within primary bone marrow macrophages

**Figure 5:**
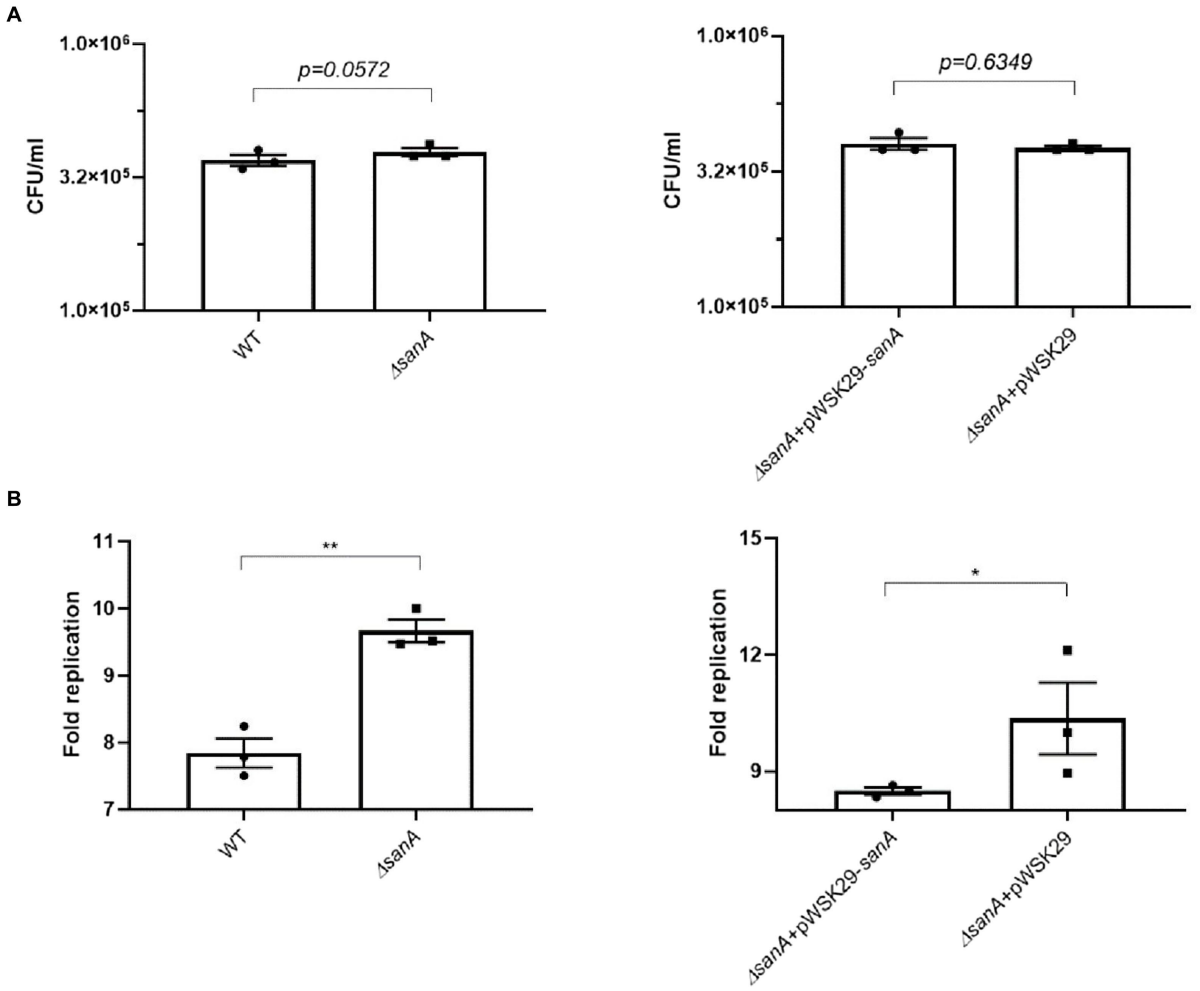
Salmonella infection of primary bone marrow macrophages (pBMDM). **(A)** invasion level of BMDM, **(B)** intracellular replication within BMDM isolated from C57BL/6 mice of *S. Typhimurium* 4/74, its deletion mutant *ΔsanA* and *ΔsanA* transformed with empty pWSK29 plasmid or vector with *sanA*. The fold replication was determined by comparing the bacterial population within macrophages after a 24 h incubation period to that after a 2 h initial incubation. The data are shown as mean values and SEM of three separate experiments of intracellular replication. Statistical differences were analyzed by Student’s *t* test (*p < 0.05; **p < 0.01).

Alterations in bacterial membranes significantly influence antimicrobial efficacy and bacterial replication within phagocytes (Ernst et al., 1999). To further investigate this, we monitored *Salmonella* replication in primary BMDMs, which provide a relevant physiological context to examine the interactions between *Salmonella* and host cells. Our results showed that the uptake of *S.* Typhimurium by BMDMs was similar for the WT and *ΔsanA* (*p = 0.0572;* Figure 5A), but the mutant exhibited a significantly increased number of intracellular bacteria 24 h post-infection (*p = 0.0051*; Figure 5B). Furthermore, we observed a marked difference between *ΔsanA +* pWSK29 and *ΔsanA* + pWSK29-*sanA*, whereby expression of *sanA* reduced replication (*p = 0.0351*; Figure 5B).

## Discussion

Multidrug resistance among bacteria, including prominent species such as *Salmonella*, *Pseudomonas*, and *Campylobacter*, constitutes a major public health concern (Centers for Disease Control and Prevention, 2019). These bacteria utilize diverse mechanisms, including the creation of enzymatic barriers and altering membrane compositions, to mitigate the impact of surface disinfection or antibiotic therapies (Reygaert, 2018). Gram-negative bacteria exhibit a complex cellular envelope with the OM forming an extra line of defense. Its permeability properties have significant implications for the bacterium’s sensitivity to antibiotics (Silhavy et al., 2010). Moreover, although less studied, the IM proteins play a crucial role in coordinating processes pivotal for bacterial survival and resistance to extreme environmental conditions. Mutations in the genes encoding these proteins could increase membrane permeability, thereby promoting antibiotic resistance (Ize et al., 2003; Boughner and Doerrler, 2012).

Our study emphasizes on a lesser-known protein, SanA, and examines its role in altering the physicochemical properties of bacterial membranes, which consequently affect the bacterium’s resistance phenotype.

SanA is composed of 239 amino acids and is predicted to primarily localize in the inner membrane, featuring a small N-terminal cytoplasmic domain spanning just six amino acids. It possesses a single transmembrane helix, with the remainder of the protein predominantly situated in the periplasmic space (Krogh et al., 2001; Petersen et al., 2011). Within the periplasmic part, SanA harbors a DUF218 domain designated as a domain of the unknown function (Finn et al., 2014). DUF218 domains contain multiple charged amino acids, implying potential enzymatic activity, and are prevalent across various bacterial species. These domains are primarily associated with proteins whose functions remain elusive (Mitchell et al., 2017). SanA was initially discovered as a multicopy suppressor in response to unknown mutations that affected the OM permeability. This included not only the deletion of the *sanA* gene but also other mutations that were associated with impairments in the OM (Rida et al., 1996). Moreover, in a study on *sanA* ortholog (97% identity of nucleotide sequence), the *S.* Typhimurium *sfiX-strain* failed to grow in the presence of vancomycin in high temperature (Mouslim et al., 1998).

Thus, we initially aimed to determine how *sanA* deletion affects the growth of *S.* Typhimurium 4/74 in the presence of vancomycin and bile salts, a key substrate of McConkey medium at 37°C, what corresponds to the host’s physiological temperature. Apparently, our findings aligned with previous outcome, indicating that a *Salmonella* strain carrying a 10-nucleotide deletion in *sanA* displays enhanced vancomycin resistance than that with wild-type *sanA* at the same temperature (Kolenda et al., 2021). It is crucial to highlight that the variance in these findings compared to Rida et al.’s study may arise from various factors, including the higher temperature utilized in their assay and the use of a less well-characterized mutant with additional to *sanA* mutations (Rida et al., 1996). Additionally, it might stem from methodological distinctions, particularly the choice of plate material. The polypropylene plates with a neutral surface aimed to minimize non-specific binding—a critical aspect frequently overlooked in the realm of antibiotic resistance research, to ensure precise measurement of vancomycin activity (Singhal et al., 2018). Furthermore, our demonstration of detectable differences in the stationary growth phase allows us to suggest that SanA expression may occur in stress conditions, such as the late growth phase or elevated temperatures employed in prior studies.

In contrast, we observed an inverse effect with anionic bile salts, wherein the WT demonstrated higher resistance. This observation aligns with that of Langridge et al. (2009) who found that an *S.* Typhimurium *sanA* mutant exhibits increased bile sensitivity (Langridge et al., 2009). It suggests a distinct role of SanA on various chemical compounds, implying that the protein affects barrier function by altering properties of the envelope, rather than the antibiotic’s mechanism of action, sequestration, modification, or target blocking (Langridge et al., 2009). Thus, we further explored this phenomenon using the PM, which analyzed the growth of strains in the presence of 240 different agents, simultaneously. The obtained data were then validated using the microbroth dilution assay, since the Biolog phenotype microarray is a screening method and results are not as accurate as using the classical approach (Dunkley et al., 2019). Moreover, the Biolog PM assay indirectly measures bacterial growth through colorimetric signals, which may not directly correlate with the bacterial growth inhibition caused by antibiotics (Dunkley et al., 2019). Our analysis highlighted a decreased resistance trend in the *ΔsanA* to phosphomycin, detergents, and polymyxin B. Additionally, the same strain showed lower resistance to protein synthesis-targeting antibiotics, such as aminoglycosides and aminocyclitoles, as well as to transcription-related compounds such as fluoroquinolones and quinolines. Conversely, enhanced resistance was noted toward cell wall synthesis and efflux pumps-associated xenobiotics as well as DNA targeting agents, such as glycopeptides and sulfonamides.

Previously published data did not determine the role of *sanA* unequivocally, but has suggested its role in peptidoglycan synthesis (Mouslim et al., 1998). The location of the C-terminus, containing DUF218 domain with charged amino acids in the periplasm, which is the site of the cell wall synthesis, may indicate that it plays a role in blocking the activity of vancomycin at its site of action (Mitchell et al., 2017). In contrast, the hydrophobic nature of the SanA protein, suggests that it participates in the barrier functions of bacterial cell envelopes, affecting the synthesis of murein, which is essential for cell wall function and maintenance. This role was indicated by the dual effect of the *sanA* mutation—induction of vancomycin sensitivity and suppression of cell division inhibition (Rida et al., 1996; Mouslim et al., 1998). Our data revealed that *sanA* deletion resulted in higher resistance to vancomycin as well as different classes of antibiotics associated with the cell wall synthesis—ceftriaxone and carbenicillin. In contrast, the same strain revealed higher susceptibility to phosphomycin, another murein synthesis-targeting antibiotic. Since all these agents hinder bacterial growth by inhibiting peptidoglycan synthesis, each of them targets another stage of this process. Carbenicillin, and ceftriaxone are beta-lactam antibiotics, which function by mimicking the D-alanyl-D-alanine structure and binding to Penicillin-binding proteins; this prevents them from cross-linking the peptidoglycan layers and causing cell death in the final, extracytoplasmic stage of peptidoglycan synthesis (Lima et al., 2020). Unlike beta-lactam antibiotics, vancomycin affects the second stage of creating bacterial cell membranes, by targeting the d-Ala-d-Ala terminus of peptidoglycan. In turn, phosphomycin has a unique mechanism of action. It inhibits the first step in peptidoglycan synthesis by targeting the enzyme MurA (UDP-N-acetylglucosamine enolpyruvyl transferase). This enzyme catalyzes the conversion of UDP-N-acetylglucosamine to UDP-N-acetylmuramic acid, the first committed step in peptidoglycan synthesis. By inhibiting this enzyme, phosphomycin disrupts the production of peptidoglycan precursors, repressing early cell wall synthesis (Falagas et al., 2016). Thus, the role of SanA in peptidoglycan synthesis, and hence in antibiotic resistance, may be more complex than expected. Based on our *in silico* predictions and considering SanA’s putative role as a permease, its function might be similar to that of AmpG, an IM permease responsible for transporting anhydromuropeptides into the bacterial cytoplasm, contributing to peptidoglycan recycling (Jacobs et al., 1994). This would explain why the deletion of *sanA* does not confer resistance to all antibiotics targeting peptidoglycan synthesis, as demonstrated by reduced resistance to phosphomycin. It is worth noting however that the Panther database’s classification of SanA as a potential permease may not align with biological reality, given that SanA has only one transmembrane helix (Thomas et al., 2003). The same database assigns a similar classification to YdcF, a cytoplasmic protein containing a DUF218 domain (Thomas et al., 2003).

Any changes in peptidoglycan synthesis can alter the bacterial envelope structure and composition, leading to modified interactions with xenobiotics (Nikolaidis et al., 2014; Yadav et al., 2018). Since peptidoglycan is critical for maintaining the shape and structural integrity of the cell wall, interference at any stage of its synthesis, assembly, or recycling can effectively inhibit cell growth (Typas et al., 2012). It correlates with previously published data demonstrating the role of *sanA* in the cell division of a defective mutant (Mouslim et al., 1998). Additionally, changes to the murein synthesis pathway could impact the overall cell wall structure and stability, bacterial membrane permeability, or transport mechanisms, which could impact the uptake or efflux of antibiotics. The stability of the OM is maintained through tethering of the OM to the sacculus, a process that is facilitated by both covalent and non-covalent interactions between abundant OM proteins (such as Lpp, Pal, and OmpA) and peptidoglycan (Hantke and Braun, 1973; Parsons et al., 2006). Complex resistance effect, based on increased susceptibility to membrane-bound antibiotics–chlorhexidine acetate, cetylpyridinum chloride, umbelliferone, and polymyxin B confirmed this occurrence, suggesting a correlation between the IM protein, SanA, and OM, responsible for maintaining integrity of the envelope. This situation is reminiscent to that of TolA, wherein a defect in *tolA* leads to detergent sensitivities. This protein, being anchored in the IM by its hydrophobic amino-terminal 21-residue segment similar to SanA, presumably interacts through its carboxyl-terminal domain with components on the inner surface of the OM for maintaining its integrity (Levengood et al., 1991; Levengood-Freyermuth et al., 1993). Our data indicating significantly higher OM permeability of the *sanA* mutant corroborates this hypothesis.

Furthermore, the phenotype of *sanA* mutant correlates with an increased sensitivity for aminoglycosides—streptomycin, tobramycin, and aminocyclitole—spectinomycin, having the same target of action. Aminoglycoside resistance typically involves diminished uptake or decreased cellular permeability, modifications at the ribosomal binding sites, or the generation of aminoglycoside modifying enzymes (Garneau-Tsodikova and Labby, 2016). Thus, enhanced membrane permeability due to *sanA* knockout was the primary reason for the observed shifts in the resistance phenotype. Notably, we observed a reverse phenotype for all the agents tested, except tobramycin, further supporting that the resistance phenotype is more complex than initially assumed. Similarly, we demonstrated decreased resistance of the *ΔsanA* to transcription-associated antibiotics such as fluoroquinolones and quinolines. Nevertheless, the expression of *sanA* from a plasmid did not completely reverse the effects of the mutation, indicating that SanA plays only a partial role in this phenotype. Additionally, the absence of a specific SanA antibody prevents direct comparison of *sanA* expression in its plasmid and chromosomal forms. Therefore, variations in expression levels and regulatory elements could be responsible for the observed incomplete restoration of the phenotype.

Although WT bacteria exhibited resistance to a broader spectrum of xenobiotics, the mutant displayed increased resistance to replication-targeting antibiotics, bleomycin and sulfamonomethoxine. These two antibiotics have similar targets of action, but differ significantly in their physicochemical properties. Bleomycin, like vancomycin, has a notably high molecular weight (1,415 Da) and is classified as a cationic glycopeptide however, bleomycin and vancomycin have distinct mechanisms of action (Hecht, 2000). This finding further suggests that *sanA* is not directly associated with the specific action mechanisms of these xenobiotics. Instead, it seems to be linked, at least partially, with the membrane charge (Davlieva et al., 2013). As *sanA* contributes to a more positive membrane charge, it subsequently increased resistance to cationic antibiotics.

Bacterial resistance to bleomycin and sulfamonomethoxine, a derivative of sulfonamide, is mainly attributed to the Resistance-Nodulation-Division (RND) family of efflux pumps. The SanA structure does not resemble that of an RND transporter, suggesting that its absence, as observed in the mutant, may lead to the overexpression of another efflux pump that compensates for the transport of this antibiotic. Moreover, due to the neutral charge of sulfamonomethoxine, alterations in the phospholipid composition of the IM may hinder the passive diffusion of neutral antibiotics (Kadner, 1996).

Considering the distinct effects of *sanA* deletion on resistance to different classes of antibiotics, we decided to explore whether this genetic modification also affects the intracellular replication of *Salmonella* within macrophages. Macrophages are immune cells essential for host defense against bacterial infections, as they internalize and destroy them using various mechanisms, including the production of reactive oxygen and nitrogen species and antimicrobial peptides (Gordon, 1999). These substances possess bactericidal properties and disrupt bacterial cell envelope integrity and function, similar to antibiotics. Therefore, modifications affecting antibiotic resistance might also influence the bacterium’s ability to tolerate the hostile intracellular environment of a macrophage. To further explore this phenomenon, we selected a C57BL/6 primary BMDM model for *Salmonella* replication, which provides a physiologically relevant environment for studying the interactions between *Salmonella* and host cells compared to cell lines As a result, *sanA* deletion resulted in higher replication rates of *Salmonella* within primary macrophages, suggesting that the absence of *sanA* may enhance the ability of the bacterium to resist the bactericidal actions of macrophages. We suggest it is linked to alterations in the bacterial cell envelope associated with *sanA* deletion as our data suggest that *sanA* knockout leads to increased membrane hydrophilicity and positive charge. As the outer layer of bacterial cells possesses an anionic charge, most antimicrobial peptides (AMPs) effective against bacteria are cationic, enabling them to bind to the negatively charged bacterial surface (Lei et al., 2019). Consequently, bacterial resistance to AMPs often involves surface modification to reduce the negative charge, which serves as an initial defense mechanism (Peschel, 2002). Also, previously published data revealed that the efficiency of phagocytosis increases with the hydrophobicity of bacterial cells and that hydrophilic bacteria resist ingestion by phagocytes (Matz and Jürgens, 2001). Surprisingly, we did not observe significant changes in the invasiveness of the analyzed strains in the conditions we used. To better elucidate the role of *sanA* in host-pathogen interactions, it is necessary to investigate changes occurring in the bacterial envelope due to *sanA* knockout. We hypothesize that *sanA* deletion and the subsequent increase in membrane permeability may be linked to an upregulation of SPI-II and/or SPI-I genes, which are responsible for intracellular replication and invasion, respectively. Currently, this hypothesis is under investigation.

In conclusion, our study offers a crucial understanding of the dynamics of antibiotic resistance, underscoring how alterations in membrane properties influence bacterial susceptibility to various xenobiotics. The insights regarding SanA’s influence on membrane physicochemical properties shed new light on the role of membrane proteins in *Salmonella*’s resistance to environmental stressors. This highlights the importance of these proteins in comprehending bacterial pathogenicity and survival mechanisms.

## Data availability statement

The original contributions presented in the study are included in the article/Supplementary material, further inquiries can be directed to the corresponding author.

## Ethics statement

The animal study was approved by UK Home Office Project License in a Home Office designated facility. Imperial College Animal Welfare and Ethical Review Body (AWERB) granted approval for all mouse work. The study was conducted in accordance with the local legislation and institutional requirements.

## Author contributions

AA: Conceptualization, Data curation, Investigation Methodology, Validation, Visualization, Writing – original draft. RK: Conceptualization, Resources, Supervision, Writing – review & editing. KB: Investigation, Writing – review & editing. TLMT: Methodology, Resources, Writing – review & editing. JS: Investigation, Writing – review & editing. KG: Conceptualization, Data curation, Formal analysis, Funding acquisition, Project administration, Resources, Supervision, Writing – review & editing.

## References

[ref1] Alenazy R. (2022). Antibiotic resistance in Salmonella: targeting multidrug resistance by understanding efflux pumps, regulators and the inhibitors. J King Saud Univ Sci. 34:102275. doi: 10.1016/j.jksus.2022.102275

[ref2] Asmar A. T. Collet J. F. (2018). Lpp, the Braun lipoprotein, turns 50—major achievements and remaining issues. FEMS Microbiol. Lett. 365:fny199. doi: 10.1093/femsle/fny199 30107563

[ref3] Ayukekbong J. A. Ntemgwa M. Atabe A. N. (2017). The threat of antimicrobial resistance in developing countries: causes and control strategies. Antimicrob. Resist. Infect. Control 6:47. doi: 10.1186/s13756-017-0208-x, PMID: 28515903 PMC5433038

[ref4] Bailey J. D. Shaw A. McNeill E. Nicol T. Diotallevi M. Chuaiphichai S.. (2020). Isolation and culture of murine bone marrow-derived macrophages for nitric oxide and redox biology. Nitric Oxide 100-101, 17–29. doi: 10.1016/j.niox.2020.04.005, PMID: 32339668 PMC7284309

[ref5] Boughner L. A. Doerrler W. T. (2012). Multiple deletions reveal the essentiality of the DedA membrane protein family in *Escherichia coli*. Microbiology 158, 1162–1171. doi: 10.1099/mic.0.056325-0, PMID: 22301910

[ref6] Centers for Disease Control and Prevention (2019). Antibiotic resistance threats in the United States, Centers for Disease Control and Prevention (United States) 2019

[ref8] Cherepanov W. W. (1995). Gene disruption in *Escherichia coli*: Tc R and km R cassettes with the option of Flp-catalyzed excision of the antibiotic-resistance determinant. Gene 158, 9–14. doi: 10.1016/0378-1119(95)00193-a 7789817

[ref9] Datsenko K. A. Wanner B. L. (2000). One-step inactivation of chromosomal genes in *Escherichia coli* K-12 using PCR products. Proc. Natl. Acad. Sci. 97, 6640–6645. doi: 10.1073/pnas.120163297, PMID: 10829079 PMC18686

[ref10] Davlieva M. Zhang W. Arias C. A. Shamoo Y. (2013). Biochemical characterization of cardiolipin synthase mutations associated with daptomycin resistance in enterococci. Antimicrob. Agents Chemother. 57, 289–296. doi: 10.1128/AAC.01743-12, PMID: 23114777 PMC3535954

[ref11] Dunkley E. J. Chalmers J. D. Cho S. Finn T. J. Patrick W. M. (2019). Assessment of phenotype microarray plates for rapid and high-throughput analysis of collateral sensitivity networks. PloS One 14:e0219879. doi: 10.1371/journal.pone.0219879, PMID: 31851668 PMC6919586

[ref12] Ernst R. K. Guina T. Miller S. I. (1999). How Intracellular Bacteria survive: surface modifications that promote resistance to host innate immune responses. J Infect Dis 179, S326–S330. doi: 10.1086/513850 10081503

[ref13] Falagas M. E. Vouloumanou E. K. Samonis G. Vardakasa K. Z. (2016). Fosfomycin. Clin Microbiol rev. American society for. Microbiology 29, 321–347. doi: 10.1128/CMR.00068-15 PMC4786888 26960938

[ref14] Finn R. D. Bateman A. Clements J. Coggill P. Eberhardt R. Y. Eddy S. R.. (2014). Pfam: the protein families database. Nucleic Acids Res. 42, D222–D230. doi: 10.1093/nar/gkt1223, PMID: 24288371 PMC3965110

[ref15] Garneau-Tsodikova S. Labby K. J. (2016). Mechanisms of resistance to aminoglycoside antibiotics: overview and perspectives. Medchemcomm 7, 11–27. doi: 10.1039/c5md00344j, PMID: 26877861 PMC4752126

[ref16] Gordon S. (1999). Phagocytosis: The host. Stamford, CT: JAI Press.

[ref17] Guard-Bouldin J. Morales C. A. Frye J. G. Gast R. K. Musgrove M. (2007). Detection of *Salmonella enterica* subpopulations by phenotype microarray antibiotic resistance patterns. Appl. Environ. Microbiol. 73, 7753–7756. doi: 10.1128/AEM.01228-07, PMID: 17965201 PMC2168045

[ref18] Hantke K. Braun V. (1973). Covalent binding of lipid to protein diglyceride and amide-linked fatty acid at the N-terminal end of the Murein-lipoprotein of the *Escherichia coli* outer membrane Eur. J. Biochem. 34, 284–296. doi: 10.1111/j.1432-1033.1973.tb02757.x 4575979

[ref19] Havelaar A. H. Kirk M. D. Torgerson P. R. Gibb H. J. Hald T. Lake R. J.. (2015). World Health Organization global estimates and regional comparisons of the burden of foodborne disease in 2010. PLoS Med. 12:e1001923. doi: 10.1371/journal.pmed.1001923, PMID: 26633896 PMC4668832

[ref20] Hecht S. M. (2000). Bleomycin: new perspectives on the mechanism of action. J. Nat. Prod. 63, 158–168. doi: 10.1021/np990549f, PMID: 10650103

[ref21] Huerta-Cepas J. Szklarczyk D. Heller D. Hernández-Plaza A. Forslund S. K. Cook H.. (2019). Egg NOG 5.0: a hierarchical, functionally and phylogenetically annotated orthology resource based on 5090 organisms and 2502 viruses. Nucleic Acids Res. 47, D309–D314. doi: 10.1093/nar/gky1085 30418610 PMC6324079

[ref22] Ize B. Stanley N. R. Buchanan G. Palmer T. (2003). Role of the *Escherichia coli* tat pathway in outer membrane integrity. Mol. Microbiol. 48, 1183–1193. doi: 10.1046/j.1365-2958.2003.03504.x, PMID: 12787348

[ref23] Jacobs C. Huang L.-J. Bartowsky E. Normark S. Park J. T. (1994). Bacterial cell wall recycling provides cytosolic muropeptides as effectors for-lactamase induction. EMBO J. 13, 4684–4694. doi: 10.1002/j.1460-2075.1994.tb06792.x 7925310 PMC395403

[ref24] Kadner R. J. (1996). Cytoplasmic membrane. Cell. Mol. Biol. 1, 58–87.

[ref25] Käll L. Krogh A. Sonnhammer E. L. L. (2007). Advantages of combined transmembrane topology and signal peptide prediction-the Phobius web server. Nucleic Acids Res. 35, W429–W432. doi: 10.1093/nar/gkm256, PMID: 17483518 PMC1933244

[ref26] Kelley L. A. Mezulis S. Yates C. M. Wass M. N. Sternberg M. J. E. (2015). The Phyre2 web portal for protein modeling, prediction and analysis. Nat. Protoc. 10, 845–858. doi: 10.1038/nprot.2015.053, PMID: 25950237 PMC5298202

[ref27] Kolenda R. Burdukiewicz M. Wimonc M. Aleksandrowicz A. Ali A. Szabo I.. (2021). Identification of natural mutations responsible for altered infection phenotypes of *Salmonella enterica* clinical isolates by using cell line infection screens. Appl. Environ. Microbiol. 87, e02177–e02120. doi: 10.1128/AEM.02177-20, PMID: 33127819 PMC7783345

[ref28] Kristian S. A. Datta V. Weidenmaier C. Kansal R. Fedtke I. Peschel A.. (2005). D-alanylation of teichoic acids promotes group a Streptococcus antimicrobial peptide resistance, neutrophil survival, and epithelial cell invasion. J. Bacteriol. 187, 6719–6725. doi: 10.1128/JB.187.19.6719-6725.2005, PMID: 16166534 PMC1251589

[ref29] Krogh A. Larsson B. Von Heijne G. Sonnhammer E. L. L. (2001). Predicting transmembrane protein topology with a hidden Markov model: application to complete genomes. J. Mol. Biol. 305, 567–580. doi: 10.1006/jmbi.2000.4315 11152613

[ref30] Langridge G. C. Phan M. D. Turner D. J. Perkins T. T. Parts L. Haase J.. (2009). Simultaneous assay of every *Salmonella Typhi* gene using one million transposon mutants. Genome Res. 19, 2308–2316. doi: 10.1101/gr.097097.109, PMID: 19826075 PMC2792183

[ref31] Lei J. Sun L. Huang S. Zhu C. Li P. He J.. (2019). The antimicrobial peptides and their potential clinical applications. Am. J. Transl. Res. 11, 3919–3931. PMID: 31396309 PMC6684887

[ref32] Levengood S. K. Beyer W. F. Webster R. E. (1991). TolA: a membrane protein involved in colicin uptake contains an extended helical region. Nati. Acad. Sci. USA. 88, 5939–5943. doi: 10.1073/pnas.88.14.5939 PMC51997 2068069

[ref33] Levengood-Freyermuth S. K. Click E. M. Webster R. E. (1993). Role of the carboxyl-terminal domain of TolA in protein import and integrity of the outer membrane. J. Bacteriol. 175, 222–228. doi: 10.1128/jb.175.1.222-228.1993, PMID: 8416897 PMC196117

[ref34] Lima L. M. Silva B. N. M. Barbosa G. Barreiro E. J. (2020). β-Lactam antibiotics: an overview from a medicinal chemistry perspective. Eur. J. Med. Chem. 208:112829. doi: 10.1016/j.ejmech.2020.112829, PMID: 33002736

[ref35] Malinverni J. C. Silhavy T. J. (2011). Assembly of outer membrane β-barrel proteins: the bam complex. Eco Sal Plus. 4, 16–23. doi: 10.1128/ecosalplus.4.3.8 PMC4231818 26442509

[ref36] Matz C. Jürgens K. (2001). Effects of hydrophobic and electrostatic cell surface properties of bacteria on feeding rates of heterotrophic nanoflagellates. Appl. Environ. Microbiol. 67, 814–820. doi: 10.1128/AEM.67.2.814-820.2001, PMID: 11157248 PMC92652

[ref37] Mitchell A. M. Wang W. Silhavy T. J. (2017). Novel RpoS-dependent mechanisms strengthen the envelope permeability barrier during stationary phase. J. Bacteriol. 199, e00708–e00716. doi: 10.1128/JB.00708-16 27821607 PMC5198486

[ref38] Monack D. M. Raupach B. Hromockyj A. E. Falkow S. (1996). *Salmonella typhimurium* invasion induces apoptosis in infected macrophages source. Proc. Natl. Acad. Sci. U. S. A. 93, 9833–9838. doi: 10.1073/pnas.93.18.9833 8790417 PMC38515

[ref39] Mouslim C. Cano D. A. Casadesús J. (1998). The sfiX, rfe and metN genes of Salmonella typhimurium and their involvement in the his (c) pleiotropic response. Mol. Gen. Genet. 259, 46–53. doi: 10.1007/s004380050787 9738879

[ref40] Murata T. Tseng W. Guina T. Miller S. I. Nikaido H. (2007). PhoPQ-mediated regulation produces a more robust permeability barrier in the outer membrane of *Salmonella enterica* serovar typhimurium. J. Bacteriol. 189, 7213–7222. doi: 10.1128/JB.00973-07, PMID: 17693506 PMC2168427

[ref41] Nikaido H. (2003). Molecular basis of bacterial outer membrane permeability revisited. Microbiol. Mol. Biol. Rev. 67, 593–656. doi: 10.1128/mmbr.67.4.593-656.2003, PMID: 14665678 PMC309051

[ref42] Nikolaidis I. Favini-Stabile S. Dessen A. (2014). Resistance to antibiotics targeted to the bacterial cell wall. Protein Sci. 23, 243–259. doi: 10.1002/pro.2414, PMID: 24375653 PMC3945833

[ref43] O’Neil J. (2016). Antimicrobial resistance: Tackling a crisis for the health and wealth of nations. London, United Kingdom: Antimicrobial Resistance.

[ref44] Oguri T. Yeo W. S. Bae T. Lee H. (2016). Identification of envC and its cognate amidases as novel determinants of intrinsic resistance to cationic antimicrobial peptides. Antimicrob. Agents Chemother. 60, 2222–2231. doi: 10.1128/AAC.02699-15, PMID: 26810659 PMC4808223

[ref45] Papanastasiou M. Orfanoudaki G. Kountourakis N. Koukaki M. Sardis M. F. Aivaliotis M.. (2016). Rapid label-free quantitative analysis of the *E. coli* BL21(DE3) inner membrane proteome. Proteomics 16, 85–97. doi: 10.1002/pmic.201500304, PMID: 26466526

[ref46] Parsons L. M. Lin F. Orban J. (2006). Peptidoglycan recognition by pal, an outer membrane lipoprotein. Biochemistry 45, 2122–2128. doi: 10.1021/bi052227i, PMID: 16475801

[ref47] Peschel A. (2002). How do bacteria resist human antimicrobial peptides? Trends Microbiol. 10, 179–186. doi: 10.1016/s0966-842x(02)02333-8 11912025

[ref48] Petersen T. N. Brunak S. Von Heijne G. Nielsen H. (2011). SignalP 4.0: discriminating signal peptides from transmembrane regions. Nat. Methods 8, 785–786. doi: 10.1038/nmeth.1701, PMID: 21959131

[ref49] Reygaert C. (2018). An overview of the antimicrobial resistance mechanisms of bacteria. AIMS Microbiol 4, 482–501. doi: 10.3934/microbiol.2018.3.482, PMID: 31294229 PMC6604941

[ref50] Rida S. Caillet J. Alix J. H. (1996). Amplification of a novel gene, sanA, abolishes a vancomycin-sensitive defect in *Escherichia coli*. J. Bacteriol. 178, 94–102. doi: 10.1128/jb.178.1.94-102.1996, PMID: 8550448 PMC177625

[ref51] Shea A. Wolcott M. Daefler S. Rozak D. A. (2012). Biolog phenotype microarrays. Methods Mol. Biol. 881, 331–373. doi: 10.1007/978-1-61779-827-6_12 22639219

[ref52] Silhavy T. J. Kahne D. Walker S. (2010). The bacterial cell envelope. Cold Spring Harb. Perspect. Biol. 2:a000414. doi: 10.1101/cshperspect.a000414, PMID: 20452953 PMC2857177

[ref53] Singhal L. Sharma M. Verma S. Kaur R. Britto X. B. Kumar S. M.. (2018). Comparative evaluation of broth microdilution with polystyrene and glass-coated plates, agar dilution, E-test, vitek, and disk diffusion for susceptibility testing of colistin and polymyxin B on carbapenem-resistant clinical isolates of *acinetobacter baumannii*. Microb. Drug Resist. 24, 1082–1088. doi: 10.1089/mdr.2017.0251, PMID: 29406804

[ref54] Sun J. Rutherford S. T. Silhavy T. J. Huang K. C. (2022). Physical properties of the bacterial outer membrane. Nat. Rev. Microbiol. 20, 236–248. doi: 10.1038/s41579-021-00638-0, PMID: 34732874 PMC8934262

[ref55] Thomas P. D. Campbell M. J. Kejariwal A. Mi H. Karlak B. Daverman R.. (2003). PANTHER: a library of protein families and subfamilies indexed by function. Genome Res. 13, 2129–2141. doi: 10.1101/gr.772403, PMID: 12952881 PMC403709

[ref56] Thurston T. L. M. Matthews S. A. Jennings E. Alix E. Shao F. Shenoy A. R.. (2016). Growth inhibition of cytosolic Salmonella by caspase-1 and caspase-11 precedes host cell death. Nat. Commun. 7:13292. doi: 10.1038/ncomms13292, PMID: 27808091 PMC5097160

[ref57] Typas A. Banzhaf M. Gross C. A. Vollmer W. (2012). From the regulation of peptidoglycan synthesis to bacterial growth and morphology. Nat. Rev. Microbiol. 10, 123–136. doi: 10.1038/nrmicro2677, PMID: 22203377 PMC5433867

[ref58] Viau C. Le Sage V. Ting D. K. Gross J. Le Moual H. (2011). Absence of PmrAB-mediated phosphoethanolamine modifications of *Citrobacter rodentium* lipopolysaccharide affects outer membrane integrity. J. Bacteriol. 193, 2168–2176. doi: 10.1128/JB.01449-10, PMID: 21378194 PMC3133075

[ref59] Yadav A. K. Espaillat A. Cava F. (2018). Bacterial strategies to preserve cell wall integrity against environmental threats. Front. Microbiol. 9:2064. doi: 10.3389/fmicb.2018.02064, PMID: 30233540 PMC6127315

[ref60] Yu N. Y. Wagner J. R. Laird M. R. Melli G. Rey S. Lo R.. (2010). PSORTb 3.0: improved protein subcellular localization prediction with refined localization subcategories and predictive capabilities for all prokaryotes. Bioinformatics 26, 1608–1615. doi: 10.1093/bioinformatics/btq249, PMID: 20472543 PMC2887053

