# Supplementary material for: Membrane properties modulation by SanA: implications for xenobiotic resistance in Salmonella Typhimurium
Source: Front Microbiol. 2024 Jan 5;14:1340143. doi: 10.3389/fmicb.2023.1340143 (PMC10797042; doi:10.3389/fmicb.2023.1340143)
Supplement: Supplementary file 1 [file Data_Sheet_1.PDF]

**Table S1** Antibiotic resistance patterns of *S* . Typhimurium 4/74 obtained by Biolog Phenotype MicroArray™

| Compound name | Plate | Well | S. Typhimurium 4/74 WT | | | S. Typhimurium 4/74 *ΔsanA* | | | *Probability (P) of similarity to S. Typhimurium 4/74 WT* |
|---|---|---|---|---|---|---|---|---|---|
| | | | i | ii | iii | i | ii | iii | |
| Amikacin | PM11C | A01 | 292 | 290 | 302 | 302 | 298 | 296 | 0.206 |
| Amikacin | PM11C | A02 | 303 | 302 | 306 | 305 | 301 | 296 | |
| Amikacin | PM11C | A03 | 305 | 299 | 301 | 305 | 298 | 300 | |
| Amikacin | PM11C | A04 | 309 | 311 | 307 | 299 | 300 | 295 | |
| Chlortetracycline | PM11C | A05 | 292 | 300 | 303 | 298 | 297 | 298 | 0.694 |
| Chlortetracycline | PM11C | A06 | 291 | 288 | 297 | 283 | 280 | 280 | |
| Chlortetracycline | PM11C | A07 | 275 | 273 | 269 | 266 | 251 | 263 | |
| Chlortetracycline | PM11C | A08 | 242 | 270 | 268 | 278 | 278 | 264 | |
| Lincomycin | PM11C | A09 | 301 | 292 | 296 | 298 | 293 | 294 | 0.356 |
| Lincomycin | PM11C | A10 | 298 | 296 | 296 | 311 | 301 | 306 | |
| Lincomycin | PM11C | A11 | 291 | 285 | 292 | 305 | 300 | 300 | |
| Lincomycin | PM11C | A12 | 272 | 261 | 300 | 279 | 283 | 267 | |
| Amoxicillin | PM11C | B01 | 290 | 297 | 295 | 294 | 290 | 283 | 1.000 |
| Amoxicillin | PM11C | B02 | 298 | 292 | 298 | 297 | 297 | 290 | |
| Amoxicillin | PM11C | B03 | | | | | | | |
| Amoxicillin | PM11C | B04 | 12 | 17 | 13 | 21 | 23 | 17 | |
| Cloxacillin | PM11C | B05 | 307 | 303 | 308 | 304 | 299 | 296 | 0.916 |
| Cloxacillin | PM11C | B06 | 315 | 312 | 317 | 313 | 308 | 310 | |
| Cloxacillin | PM11C | B07 | 289 | 310 | 307 | 293 | 293 | 289 | |
| Cloxacillin | PM11C | B08 | 27 | 14 | 15 | 17 | 18 | 16 | |
| Lomefloxacin | PM11C | B09 | 299 | 301 | 296 | 292 | 286 | 285 | 0.856 |
| Lomefloxacin | PM11C | B10 | 301 | 301 | 298 | 288 | 288 | 285 | |
| Lomefloxacin | PM11C | B11 | 288 | 274 | 291 | 263 | 263 | 263 | |
| Lomefloxacin | PM11C | B12 | 20 | 20 | 21 | 28 | 33 | 29 | |
| Bleomycin | PM11C | C01 | 294 | 306 | 289 | 288 | 290 | 288 | 0.000 |
| Bleomycin | PM11C | C02 | 292 | 297 | 300 | 285 | 282 | 280 | |

| | | | | | | | | | |
|---|---|---|---|---|---|---|---|---|---|
| Bleomycin | PM11C | C03 | 291 | 299 | 298 | 285 | 283 | 282 | |
| Bleomycin | PM11C | C04 | 292 | 297 | 298 | 284 | 282 | 280 | |
| Colistin | PM11C | C05 | 301 | 306 | 304 | 302 | 298 | 297 | 0.944 |
| Colistin | PM11C | C06 | 304 | 303 | 306 | 304 | 299 | 294 | |
| Colistin | PM11C | C07 | 286 | 273 | | 286 | 271 | | |
| Colistin | PM11C | C08 | 15 | 17 | 18 | 15 | 12 | 11 | |
| Minocycline | PM11C | C09 | 287 | 291 | 295 | 282 | 273 | 275 | 0.820 |
| Minocycline | PM11C | C10 | 281 | 288 | 291 | 270 | 265 | 259 | |
| Minocycline | PM11C | C11 | 64 | 23 | 29 | 23 | 28 | 27 | |
| Minocycline | PM11C | C12 | 34 | 32 | 29 | 35 | 29 | 32 | |
| Capreomycin | PM11C | D01 | 294 | 301 | 299 | 307 | 298 | 308 | 0.842 |
| Capreomycin | PM11C | D02 | 289 | 291 | 296 | 290 | 290 | 280 | |
| Capreomycin | PM11C | D03 | 294 | 293 | 302 | 297 | 288 | 287 | |
| Capreomycin | PM11C | D04 | 278 | 246 | 285 | 282 | 283 | 271 | |
| Demeclocyline | PM11C | D05 | 301 | 295 | 300 | 295 | 290 | 287 | 0.360 |
| Demeclocyline | PM11C | D06 | 301 | 291 | 297 | 292 | 286 | 276 | |
| Demeclocyline | PM11C | D07 | 303 | 289 | 308 | 277 | 257 | 270 | |
| Demeclocyline | PM11C | D08 | | 67 | 86 | 24 | 37 | 14 | |
| Nafcillin | PM11C | D09 | 295 | 289 | 298 | 288 | 285 | 285 | 0.241 |
| Nafcillin | PM11C | D10 | 289 | 293 | 296 | 290 | 285 | 279 | |
| Nafcillin | PM11C | D11 | 303 | 298 | 296 | 290 | 283 | 282 | |
| Nafcillin | PM11C | D12 | 141 | 157 | | 20 | 55 | 21 | |
| Cefazolin | PM11C | E01 | 287 | 286 | 288 | 290 | 285 | 299 | 0.128 |
| Cefazolin | PM11C | E02 | 285 | 290 | 295 | 295 | 302 | 292 | |
| Cefazolin | PM11C | E03 | 290 | 291 | 293 | 301 | 295 | 290 | |
| Cefazolin | PM11C | E04 | 299 | 299 | 298 | 297 | 299 | 295 | |
| Enoxacin | PM11C | E05 | 301 | 299 | 303 | 291 | 289 | 291 | 0.430 |
| Enoxacin | PM11C | E06 | 297 | 295 | 297 | 303 | 298 | 296 | |
| Enoxacin | PM11C | E07 | 287 | | 277 | 19 | 59 | | |
| Enoxacin | PM11C | E08 | 19 | 21 | 22 | 25 | 22 | 20 | |
| Nalidixic acid | PM11C | E09 | 289 | 288 | 293 | 292 | 283 | 284 | 0.988 |
| Nalidixic acid | PM11C | E10 | 262 | 262 | 272 | 274 | 267 | 265 | |
| Nalidixic acid | PM11C | E11 | 17 | 12 | 22 | 14 | 18 | 17 | |
| Nalidixic acid | PM11C | E12 | 24 | 18 | 26 | 22 | 17 | 22 | |
| Chloramphenicol | PM11C | F01 | 273 | 275 | 276 | 290 | 278 | 287 | 0.874 |
| Chloramphenicol | PM11C | F02 | 270 | 240 | 265 | 263 | 278 | 255 | |

| | | | | | | | | | |
|---|---|---|---|---|---|---|---|---|---|
| Chloramphenicol | PM11C | F03 | 49 | 40 | 48 | 20 | | 18 | |
| Chloramphenicol | PM11C | F04 | 16 | 15 | 21 | 14 | 19 | 12 | |
| Erythromycin | PM11C | F05 | 301 | 300 | 304 | 294 | 291 | 292 | 0.969 |
| Erythromycin | PM11C | F06 | 308 | 310 | 309 | 319 | 301 | 310 | |
| Erythromycin | PM11C | F07 | 280 | 284 | 291 | 284 | 283 | 279 | |
| Erythromycin | PM11C | F08 | 271 | 265 | 267 | 284 | 279 | 271 | |
| Neomycin | PM11C | F09 | 284 | 283 | 276 | 281 | 287 | 282 | 0.937 |
| Neomycin | PM11C | F10 | 271 | 272 | 269 | 278 | 269 | 271 | |
| Neomycin | PM11C | F11 | 270 | 266 | 273 | 287 | 286 | 283 | |
| Neomycin | PM11C | F12 | 238 | | 258 | | 216 | 212 | |
| Ceftriaxone | PM11C | G01 | 282 | 280 | 281 | 296 | 290 | 293 | 0.008 |
| Ceftriaxone | PM11C | G02 | 275 | 285 | 296 | 294 | 301 | 296 | |
| Ceftriaxone | PM11C | G03 | 291 | 284 | 294 | 302 | 299 | 294 | |
| Ceftriaxone | PM11C | G04 | 296 | 299 | 296 | 297 | 297 | 290 | |
| Gentamicin | PM11C | G05 | 304 | 282 | 293 | 280 | 289 | 289 | 0.977 |
| Gentamicin | PM11C | G06 | 301 | 293 | 289 | 290 | 299 | 298 | |
| Gentamicin | PM11C | G07 | 296 | 297 | 304 | 310 | 298 | 296 | |
| Gentamicin | PM11C | G08 | 295 | 299 | 299 | 303 | 300 | 299 | |
| Potassium tellurite | PM11C | G09 | 281 | 274 | 277 | 282 | 283 | 277 | 0.088 |
| Potassium tellurite | PM11C | G10 | 273 | 293 | 268 | 277 | 281 | 284 | |
| Potassium tellurite | PM11C | G11 | 286 | 278 | 284 | 300 | 300 | 295 | |
| Potassium tellurite | PM11C | G12 | 293 | 296 | 299 | 304 | 309 | 307 | |
| Cephalothin | PM11C | H01 | 310 | 296 | 297 | 298 | 293 | 291 | 0.824 |
| Cephalothin | PM11C | H02 | 288 | 306 | 288 | 282 | 303 | 272 | |
| Cephalothin | PM11C | H03 | 294 | | 290 | | 242 | 286 | |
| Cephalothin | PM11C | H04 | 24 | 22 | 24 | 15 | 11 | 11 | |
| Kanamycin | PM11C | H05 | 293 | 295 | 299 | 291 | 295 | 292 | 0.930 |
| Kanamycin | PM11C | H06 | 295 | 293 | 290 | 297 | 292 | 282 | |
| Kanamycin | PM11C | H07 | 292 | 290 | 307 | 290 | 293 | 298 | |
| Kanamycin | PM11C | H08 | 289 | 277 | 276 | 293 | 286 | 284 | |
| Ofloxacin | PM11C | H09 | 290 | 287 | 295 | 282 | 286 | 278 | 0.850 |
| Ofloxacin | PM11C | H10 | 304 | 299 | 293 | 288 | 293 | 281 | |
| Ofloxacin | PM11C | H11 | 295 | 284 | 302 | 288 | 286 | 288 | |
| Ofloxacin | PM11C | H12 | 38 | 36 | 35 | 23 | 29 | 26 | |
| Penicillin G | PM12B | A01 | 265 | 303 | 304 | 306 | 301 | 278 | 0.908 |
| Penicillin G | PM12B | A02 | | | | | | | |

| | | | | | | | | | |
|---|---|---|---|---|---|---|---|---|---|
| Penicillin G | PM12B | A03 | 14 | 24 | 12 | 29 | 21 | 20 | |
| Penicillin G | PM12B | A04 | 14 | 15 | 11 | 28 | 29 | 18 | |
| Tetracycline | PM12B | A05 | 306 | 305 | 299 | 304 | 299 | 301 | 0.111 |
| Tetracycline | PM12B | A06 | 306 | 305 | 301 | 301 | 295 | 298 | |
| Tetracycline | PM12B | A07 | 297 | 296 | 298 | 299 | 290 | 297 | |
| Tetracycline | PM12B | A08 | 308 | 321 | 323 | 312 | 298 | 310 | |
| Carbenicillin | PM12B | A09 | 303 | 296 | 298 | 296 | 293 | 292 | 0.220 |
| Carbenicillin | PM12B | A10 | 296 | 296 | 299 | 302 | 304 | 299 | |
| Carbenicillin | PM12B | A11 | 295 | 299 | 303 | 299 | 296 | 305 | |
| Carbenicillin | PM12B | A12 | | 83 | 24 | 246 | 290 | 268 | |
| Oxacillin | PM12B | B01 | 302 | 301 | 291 | 294 | 290 | 291 | 0.936 |
| Oxacillin | PM12B | B02 | 306 | 302 | 307 | 293 | 287 | 293 | |
| Oxacillin | PM12B | B03 | 282 | 269 | 266 | 272 | 261 | 259 | |
| Oxacillin | PM12B | B04 | 11 | 16 | 14 | 30 | 28 | 21 | |
| Penimepicycline | PM12B | B05 | 302 | 298 | 304 | 294 | 292 | 289 | 0.003 |
| Penimepicycline | PM12B | B06 | 301 | 309 | 311 | 300 | 301 | 299 | |
| Penimepicycline | PM12B | B07 | 296 | 304 | 307 | 294 | 274 | 299 | |
| Penimepicycline | PM12B | B08 | | 299 | 304 | 296 | | 297 | |
| Polymyxin B | PM12B | B09 | 304 | 305 | 301 | 288 | 290 | 284 | 0.039 |
| Polymyxin B | PM12B | B10 | 299 | 297 | 307 | 288 | 286 | 285 | |
| Polymyxin B | PM12B | B11 | 293 | 293 | 293 | 275 | 273 | 270 | |
| Polymyxin B | PM12B | B12 | | 244 | 277 | 234 | | 260 | |
| Paromomycin | PM12B | C01 | 291 | 289 | 288 | 293 | 283 | 285 | 0.992 |
| Paromomycin | PM12B | C02 | | | | | | | |
| Paromomycin | PM12B | C03 | 18 | 35 | 21 | 17 | 35 | 14 | |
| Paromomycin | PM12B | C04 | 16 | 14 | 17 | 20 | 22 | 14 | |
| Vancomycin | PM12B | C05 | 305 | 302 | 302 | 297 | 290 | 296 | 0.046 |
| Vancomycin | PM12B | C06 | 308 | 306 | 305 | 297 | 299 | 295 | |
| Vancomycin | PM12B | C07 | 307 | 300 | 302 | 294 | 293 | 287 | |
| Vancomycin | PM12B | C08 | 274 | 292 | 282 | 276 | 279 | 287 | |
| D,L-Serine Hydroxamate | PM12B | C09 | 303 | 301 | 300 | 288 | 288 | 283 | 0.001 |
| D,L-Serine Hydroxamate | PM12B | C10 | 294 | 289 | 294 | 270 | 273 | 268 | |
| D,L-Serine Hydroxamate | PM12B | C11 | 283 | 283 | 290 | 261 | 263 | 260 | |
| D,L-Serine Hydroxamate | PM12B | C12 | 275 | 270 | 274 | 270 | 267 | 253 | |
| Sisomicin | PM12B | D01 | 292 | 289 | 299 | 306 | 291 | 299 | 0.098 |
| Sisomicin | PM12B | D02 | 291 | 290 | 279 | 283 | 285 | 278 | |

| | | | | | | | | | |
|---|---|---|---|---|---|---|---|---|---|
| Sisomicin | PM12B | D03 | 298 | 301 | 293 | 292 | 294 | 289 | |
| Sisomicin | PM12B | D04 | 306 | 301 | 296 | 286 | 283 | 286 | |
| Sulfamethazine | PM12B | D05 | 305 | 301 | 294 | 293 | 294 | 289 | 0.000 |
| Sulfamethazine | PM12B | D06 | 306 | 311 | 300 | 298 | 297 | 300 | |
| Sulfamethazine | PM12B | D07 | 309 | 304 | 299 | 293 | 287 | 287 | |
| Sulfamethazine | PM12B | D08 | 305 | 302 | 300 | 288 | 290 | 291 | |
| Novobiocin | PM12B | D09 | 294 | 301 | 288 | 291 | 284 | 262 | 0.447 |
| Novobiocin | PM12B | D10 | 296 | 298 | 292 | 280 | 285 | 286 | |
| Novobiocin | PM12B | D11 | 282 | 279 | 281 | 281 | 272 | 277 | |
| Novobiocin | PM12B | D12 | 227 | 246 | 215 | 215 | 239 | 222 | |
| 2,4-Diamino-6,7-Diisopropylpteridine | PM12B | E01 | 295 | 284 | 299 | 303 | 297 | 305 | 0.202 |
| 2,4-Diamino-6,7-Diisopropylpteridine | PM12B | E02 | 290 | 288 | 291 | 291 | 303 | 292 | |
| 2,4-Diamino-6,7-Diisopropylpteridine | PM12B | E03 | 278 | 295 | 281 | 290 | 295 | 291 | |
| 2,4-Diamino-6,7-Diisopropylpteridine | PM12B | E04 | 279 | 275 | 269 | 280 | 278 | 266 | |
| Sulfadiazine | PM12B | E05 | 303 | 305 | 304 | 295 | 290 | 291 | 0.000 |
| Sulfadiazine | PM12B | E06 | 303 | 296 | 295 | 294 | 299 | 300 | |
| Sulfadiazine | PM12B | E07 | 299 | 297 | 299 | 288 | 290 | 287 | |
| Sulfadiazine | PM12B | E08 | 304 | 300 | 298 | 294 | 292 | 291 | |
| Benzethonium Chloride | PM12B | E09 | 303 | 297 | 292 | 289 | 286 | 277 | 0.753 |
| Benzethonium Chloride | PM12B | E10 | 292 | 294 | 292 | 287 | 295 | 291 | |
| Benzethonium Chloride | PM12B | E11 | | 18 | 15 | 13 | 18 | 19 | |
| Benzethonium Chloride | PM12B | E12 | 54 | 27 | 24 | 30 | 27 | 27 | |
| Tobramycin | PM12B | F01 | 283 | 281 | 277 | 299 | 290 | 290 | 0.011 |
| Tobramycin | PM12B | F02 | 262 | 290 | 290 | 282 | 290 | 278 | |
| Tobramycin | PM12B | F03 | 293 | 276 | 279 | 298 | 297 | 300 | |
| Tobramycin | PM12B | F04 | 271 | 291 | | | 286 | 295 | |
| Sulfathiazole | PM12B | F05 | 293 | 300 | 286 | 290 | 288 | 276 | 0.898 |
| Sulfathiazole | PM12B | F06 | 310 | 296 | 299 | 301 | 299 | 301 | |
| Sulfathiazole | PM12B | F07 | 298 | 292 | 283 | 296 | 294 | 290 | |
| Sulfathiazole | PM12B | F08 | 295 | 298 | 253 | 290 | 294 | 291 | |
| 5-Fluoroorotic Acid | PM12B | F09 | 302 | 289 | 264 | 275 | 290 | 289 | 0.452 |
| 5-Fluoroorotic Acid | PM12B | F10 | 255 | 265 | 272 | 260 | 262 | 263 | |
| 5-Fluoroorotic Acid | PM12B | F11 | 251 | 255 | 258 | 273 | 270 | 268 | |
| 5-Fluoroorotic Acid | PM12B | F12 | 265 | 271 | 260 | 266 | 268 | 270 | |

| | | | | | | | | | | |
|---|---|---|---|---|---|---|---|---|---|---|
| Spectinomycin | PM12B | G01 | 273 | 279 | 273 | 285 | 288 | 290 | 0.021 |
| Spectinomycin | PM12B | G02 | 267 | 269 | 274 | 286 | 275 | 261 | |
| Spectinomycin | PM12B | G03 | 276 | 269 | 263 | 277 | 287 | 279 | |
| Spectinomycin | PM12B | G04 | 277 | 276 | 275 | 271 | 280 | 275 | |
| Sulfamethoxazole | PM12B | G05 | 285 | 309 | 299 | 303 | 303 | 279 | 0.965 |
| Sulfamethoxazole | PM12B | G06 | 312 | 292 | 296 | 309 | 304 | 293 | |
| Sulfamethoxazole | PM12B | G07 | 306 | 302 | 301 | 307 | 296 | 295 | |
| Sulfamethoxazole | PM12B | G08 | 308 | 310 | 304 | 316 | 304 | 317 | |
| L-Aspartic-b-Hydroxamate | PM12B | G09 | 304 | 291 | 288 | 290 | 295 | 291 | 0.146 |
| L-Aspartic-b-Hydroxamate | PM12B | G10 | 279 | 284 | 280 | 288 | 291 | 285 | |
| L-Aspartic-b-Hydroxamate | PM12B | G11 | 259 | 262 | 259 | 283 | 280 | 279 | |
| L-Aspartic-b-Hydroxamate | PM12B | G12 | | | | | | | |
| Spiramycin | PM12B | H01 | 288 | 302 | 304 | 296 | 291 | 294 | 0.738 |
| Spiramycin | PM12B | H02 | 292 | 300 | 292 | 300 | 295 | 300 | |
| Spiramycin | PM12B | H03 | 266 | 260 | 257 | 264 | 275 | 227 | |
| Spiramycin | PM12B | H04 | 25 | 47 | 24 | 46 | | 19 | |
| Rifampicin | PM12B | H05 | 311 | 299 | 286 | 309 | 305 | 305 | 0.306 |
| Rifampicin | PM12B | H06 | 309 | 292 | 308 | 313 | 298 | 292 | |
| Rifampicin | PM12B | H07 | 317 | 313 | 300 | 315 | 312 | 316 | |
| Rifampicin | PM12B | H08 | 293 | 301 | 303 | 307 | 303 | 300 | |
| Dodecyltrimethyl Ammonium Bromide | PM12B | H09 | 296 | 300 | 300 | 295 | 294 | 280 | 0.551 |
| Dodecyltrimethyl Ammonium Bromide | PM12B | H10 | 306 | 290 | 297 | 287 | 284 | 273 | |
| Dodecyltrimethyl Ammonium Bromide | PM12B | H11 | 308 | 312 | 307 | 304 | 302 | 295 | |
| Dodecyltrimethyl Ammonium Bromide | PM12B | H12 | 45 | | 39 | 26 | 51 | 26 | |
| Ampicillin | PM13B | A01 | 287 | 290 | 293 | 305 | 298 | 299 | 0.750 |
| Ampicillin | PM13B | A02 | 298 | 305 | 301 | 304 | 306 | 296 | |
| Ampicillin | PM13B | A03 | 305 | 299 | 304 | 307 | 307 | 299 | |
| Ampicillin | PM13B | A04 | 307 | 309 | 313 | 303 | 302 | 295 | |
| Dequalinium | PM13B | A05 | 306 | 303 | 307 | 311 | 309 | 304 | 0.430 |
| Dequalinium | PM13B | A06 | 311 | 304 | 311 | 309 | 308 | 304 | |
| Dequalinium | PM13B | A07 | 298 | 303 | 307 | 314 | 307 | 304 | |
| Dequalinium | PM13B | A08 | 306 | 305 | 311 | 307 | 305 | 304 | |
| Nickel chloride | PM13B | A09 | 291 | 297 | 295 | 300 | 296 | 294 | 0.823 |

| | | | | | | | | | |
|---|---|---|---|---|---|---|---|---|---|
| Nickel chloride | PM13B | A10 | 287 | 292 | 292 | 305 | 306 | 295 | |
| Nickel chloride | PM13B | A11 | 292 | 280 | 287 | 293 | 295 | 294 | |
| Nickel chloride | PM13B | A12 | | 33 | 30 | 34 | 32 | 49 | |
| Azlocillin | PM13B | B01 | 298 | 297 | 293 | 286 | 283 | 288 | 0.119 |
| Azlocillin | PM13B | B02 | 299 | 304 | 300 | 294 | 299 | 286 | |
| Azlocillin | PM13B | B03 | 315 | 306 | 313 | 288 | 305 | 292 | |
| Azlocillin | PM13B | B04 | 287 | 261 | 274 | 277 | 272 | 274 | |
| 2,2`-Dipyridyl | PM13B | B05 | 302 | 275 | 306 | 320 | 322 | 280 | 0.905 |
| 2,2`-Dipyridyl | PM13B | B06 | 24 | 17 | 18 | 26 | 34 | 24 | |
| 2,2`-Dipyridyl | PM13B | B07 | 24 | 14 | 18 | 18 | 32 | 17 | |
| 2,2`-Dipyridyl | PM13B | B08 | 24 | 21 | 18 | 19 | 20 | 24 | |
| Oxolinic acid | PM13B | B09 | 301 | 305 | 307 | 297 | 303 | 290 | 0.876 |
| Oxolinic acid | PM13B | B10 | 299 | 302 | 304 | 294 | 292 | 283 | |
| Oxolinic acid | PM13B | B11 | 279 | 265 | 270 | 255 | 257 | 248 | |
| Oxolinic acid | PM13B | B12 | 23 | 22 | 21 | 26 | 27 | 34 | |
| 6-Mercaptopurine | PM13B | C01 | 282 | 274 | 281 | 275 | 274 | 277 | 0.356 |
| 6-Mercaptopurine | PM13B | C02 | 265 | 265 | 264 | 263 | 259 | 251 | |
| 6-Mercaptopurine | PM13B | C03 | 258 | 268 | 267 | 270 | 265 | 255 | |
| 6-Mercaptopurine | PM13B | C04 | | | | | | | |
| Doxycycline | PM13B | C05 | 301 | 293 | 303 | 299 | 294 | 296 | 0.751 |
| Doxycycline | PM13B | C06 | 298 | 297 | 304 | 292 | 296 | 288 | |
| Doxycycline | PM13B | C07 | 295 | 263 | 287 | 223 | 225 | 249 | |
| Doxycycline | PM13B | C08 | 24 | 23 | 21 | 22 | 19 | 16 | |
| Potassium chromate | PM13B | C09 | 287 | 279 | 284 | 280 | 280 | 278 | 0.261 |
| Potassium chromate | PM13B | C10 | 271 | 266 | 271 | 262 | 260 | 260 | |
| Potassium chromate | PM13B | C11 | 248 | 259 | 270 | 22 | | 22 | |
| Potassium chromate | PM13B | C12 | 83 | 36 | 36 | 41 | 34 | 37 | |
| Cefuroxime | PM13B | D01 | 293 | 292 | 286 | 292 | 296 | 304 | 0.983 |
| Cefuroxime | PM13B | D02 | 302 | 301 | 307 | 292 | 298 | 279 | |
| Cefuroxime | PM13B | D03 | 264 | 267 | 265 | 287 | 276 | 282 | |
| Cefuroxime | PM13B | D04 | 29 | 24 | 25 | 23 | 24 | 15 | |
| 5-Fluorouracil | PM13B | D05 | 303 | 295 | 301 | 298 | 300 | 291 | 0.006 |
| 5-Fluorouracil | PM13B | D06 | 308 | 303 | 308 | 302 | 301 | 299 | |
| 5-Fluorouracil | PM13B | D07 | 302 | 299 | 305 | 298 | 301 | 289 | |
| 5-Fluorouracil | PM13B | D08 | 301 | 300 | 301 | 298 | 292 | 282 | |
| Rolitetracycline | PM13B | D09 | 301 | 284 | 296 | 287 | 288 | 292 | 0.439 |

| | | | | | | | | | |
|---|---|---|---|---|---|---|---|---|---|
| Rolitetracycline | PM13B | D10 | 278 | 289 | 292 | 270 | 283 | 271 | |
| Rolitetracycline | PM13B | D11 | 258 | 268 | 296 | 242 | 255 | 237 | |
| Rolitetracycline | PM13B | D12 | 99 | 101 | 144 | 29 | 57 | 37 | |
| Cytosine arabinoside | PM13B | E01 | 287 | 286 | 290 | 290 | 285 | 299 | 0.021 |
| Cytosine arabinoside | PM13B | E02 | 270 | 284 | 280 | 287 | 297 | 283 | |
| Cytosine arabinoside | PM13B | E03 | 289 | 281 | 284 | 296 | 290 | 286 | |
| Cytosine arabinoside | PM13B | E04 | 284 | 287 | 289 | 295 | 289 | 282 | |
| Geneticin (G418) | PM13B | E05 | 306 | 302 | 308 | 302 | 301 | 288 | 0.330 |
| Geneticin (G418) | PM13B | E06 | 296 | 297 | 302 | 303 | 302 | 307 | |
| Geneticin (G418) | PM13B | E07 | 301 | 295 | 298 | 294 | 294 | 289 | |
| Geneticin (G418) | PM13B | E08 | 292 | 296 | 298 | 301 | 295 | 287 | |
| Ruthenium red | PM13B | E09 | 298 | 299 | 296 | 291 | 290 | 295 | 0.013 |
| Ruthenium red | PM13B | E10 | 290 | 294 | 296 | 295 | 294 | 289 | |
| Ruthenium red | PM13B | E11 | 295 | 295 | 295 | 289 | 298 | 284 | |
| Ruthenium red | PM13B | E12 | 301 | 300 | 302 | 291 | 300 | 293 | |
| Cesium chloride | PM13B | F01 | 284 | 281 | 280 | 288 | 286 | 292 | 0.003 |
| Cesium chloride | PM13B | F02 | 278 | 280 | 283 | 290 | 293 | 287 | |
| Cesium chloride | PM13B | F03 | 282 | 284 | 294 | 305 | 299 | 304 | |
| Cesium chloride | PM13B | F04 | 277 | 292 | 279 | 291 | 281 | 285 | |
| Glycine | PM13B | F05 | 306 | 301 | 299 | 298 | 300 | 291 | 0.450 |
| Glycine | PM13B | F06 | 281 | 292 | 304 | 301 | 302 | 297 | |
| Glycine | PM13B | F07 | 295 | 291 | 273 | 282 | 291 | 296 | |
| Glycine | PM13B | F08 | 272 | 275 | 284 | 291 | 288 | 275 | |
| Thallium (I) acetate | PM13B | F09 | 301 | 287 | 288 | 299 | 299 | 300 | 0.139 |
| Thallium (I) acetate | PM13B | F10 | 267 | 263 | 266 | 265 | 272 | 272 | |
| Thallium (I) acetate | PM13B | F11 | 261 | 262 | 269 | 283 | 285 | 280 | |
| Thallium (I) acetate | PM13B | F12 | | | | | | | |
| Cobalt chloride | PM13B | G01 | 287 | 292 | 294 | 305 | 295 | 311 | 0.893 |
| Cobalt chloride | PM13B | G02 | 289 | 288 | 284 | 295 | 303 | 294 | |
| Cobalt chloride | PM13B | G03 | 301 | 272 | 293 | 305 | 296 | 288 | |
| Cobalt chloride | PM13B | G04 | 86 | 63 | 74 | 60 | 87 | 52 | |
| Manganese (II) chloride | PM13B | G05 | 294 | 279 | 316 | 305 | 311 | 299 | 0.970 |
| Manganese (II) chloride | PM13B | G06 | 298 | 287 | 288 | 291 | 294 | 297 | |
| Manganese (II) chloride | PM13B | G07 | 298 | 289 | 297 | 300 | 295 | 290 | |
| Manganese (II) chloride | PM13B | G08 | 152 | 134 | 156 | 134 | 157 | 128 | |
| Trifluoperazine | PM13B | G09 | 295 | 294 | 296 | 292 | 293 | 290 | 0.139 |

| | | | | | | | | | |
|---|---|---|---|---|---|---|---|---|---|
| Trifluoperazine | PM13B | G10 | 289 | 281 | 284 | 286 | 292 | 291 | |
| Trifluoperazine | PM13B | G11 | 282 | 291 | 291 | 304 | 308 | 310 | |
| Trifluoperazine | PM13B | G12 | 309 | 307 | 310 | 313 | 323 | 309 | |
| Cupric chloride | PM13B | H01 | 304 | 315 | 290 | 303 | 304 | 305 | 0.995 |
| Cupric chloride | PM13B | H02 | 266 | 288 | 301 | 286 | 287 | 285 | |
| Cupric chloride | PM13B | H03 | 130 | 148 | 151 | 129 | 146 | 134 | |
| Cupric chloride | PM13B | H04 | 60 | 65 | 65 | 80 | 56 | 65 | |
| Moxalactam | PM13B | H05 | 299 | 292 | 293 | 306 | 306 | 311 | 0.876 |
| Moxalactam | PM13B | H06 | 290 | 310 | 306 | 314 | 313 | 302 | |
| Moxalactam | PM13B | H07 | | 19 | 18 | 11 | 60 | 11 | |
| Moxalactam | PM13B | H08 | 56 | 20 | 18 | 13 | 13 | 19 | |
| Tylosin | PM13B | H09 | 301 | 295 | 298 | 292 | 291 | 286 | 0.467 |
| Tylosin | PM13B | H10 | 299 | 310 | 297 | 292 | 291 | 286 | |
| Tylosin | PM13B | H11 | 270 | 286 | 298 | 291 | 289 | 286 | |
| Tylosin | PM13B | H12 | 254 | 254 | 237 | 235 | 245 | 228 | |
| Acriflavine | PM14A | A01 | 293 | 271 | 272 | 305 | 307 | 308 | 0.733 |
| Acriflavine | PM14A | A02 | 291 | 303 | 305 | 310 | 299 | 303 | |
| Acriflavine | PM14A | A03 | 318 | 323 | 324 | 315 | 309 | 313 | |
| Acriflavine | PM14A | A04 | 336 | 341 | 337 | 326 | 326 | 324 | |
| Furaltadone | PM14A | A05 | 299 | 306 | 308 | 307 | 302 | 303 | 0.490 |
| Furaltadone | PM14A | A06 | 299 | 308 | 309 | 305 | 297 | 301 | |
| Furaltadone | PM14A | A07 | 300 | 296 | 304 | 299 | 294 | 293 | |
| Furaltadone | PM14A | A08 | 289 | 284 | 290 | 290 | 289 | 286 | |
| Sanguinarine | PM14A | A09 | 302 | 302 | 301 | 303 | 302 | 293 | 0.879 |
| Sanguinarine | PM14A | A10 | 291 | 290 | 288 | 302 | 301 | 301 | |
| Sanguinarine | PM14A | A11 | 295 | 291 | 293 | 303 | 302 | 299 | |
| Sanguinarine | PM14A | A12 | | 83 | 80 | 95 | 88 | 114 | |
| 9-Aminoacridine | PM14A | B01 | 308 | 311 | 310 | 296 | 292 | 293 | 0.197 |
| 9-Aminoacridine | PM14A | B02 | 308 | 311 | 308 | 302 | 296 | 291 | |
| 9-Aminoacridine | PM14A | B03 | 317 | 317 | 314 | 300 | 297 | 299 | |
| 9-Aminoacridine | PM14A | B04 | 238 | 224 | | 232 | 234 | 216 | |
| Fusaric Acid | PM14A | B05 | 312 | 312 | 307 | 298 | 290 | 282 | 0.004 |
| Fusaric Acid | PM14A | B06 | 298 | 289 | 297 | 298 | 299 | 288 | |
| Fusaric Acid | PM14A | B07 | 304 | 311 | 310 | 300 | 296 | 293 | |
| Fusaric Acid | PM14A | B08 | | 295 | 311 | 293 | 252 | 284 | |
| Sodium Arsenate | PM14A | B09 | 278 | 269 | 277 | 282 | 279 | 270 | 0.960 |

| | | | | | | | | | |
|---|---|---|---|---|---|---|---|---|---|
| Sodium Arsenate | PM14A | B10 | 195 | 150 | 186 | 166 | 207 | 180 | |
| Sodium Arsenate | PM14A | B11 | 62 | | 68 | 51 | 63 | 54 | |
| Sodium Arsenate | PM14A | B12 | 21 | 26 | 24 | 33 | 43 | 43 | |
| Boric Acid | PM14A | C01 | 285 | 290 | 289 | 284 | 281 | 282 | 0.892 |
| Boric Acid | PM14A | C02 | 286 | 292 | 297 | 275 | 274 | 267 | |
| Boric Acid | PM14A | C03 | 273 | 269 | 282 | 267 | 264 | 267 | |
| Boric Acid | PM14A | C04 | 14 | 13 | 15 | 14 | 27 | 23 | |
| 1-Hydroxy-Pyridine-2-thione | PM14A | C05 | 297 | 300 | 302 | 294 | 291 | 288 | 0.007 |
| 1-Hydroxy-Pyridine-2-thione | PM14A | C06 | 305 | 305 | 304 | 296 | 298 | 289 | |
| 1-Hydroxy-Pyridine-2-thione | PM14A | C07 | 306 | 301 | 304 | 294 | 288 | 288 | |
| 1-Hydroxy-Pyridine-2-thione | PM14A | C08 | 289 | 285 | 281 | 282 | 286 | 278 | |
| Sodium Cyanate | PM14A | C09 | 293 | 304 | 302 | 283 | 287 | 283 | 0.770 |
| Sodium Cyanate | PM14A | C10 | 295 | 290 | 285 | 265 | 272 | 265 | |
| Sodium Cyanate | PM14A | C11 | 265 | 270 | 281 | 256 | 246 | 247 | |
| Sodium Cyanate | PM14A | C12 | 33 | 36 | 34 | 42 | 44 | 40 | |
| Cadmium Chloride | PM14A | D01 | 299 | 292 | 294 | 296 | 296 | 289 | 0.957 |
| Cadmium Chloride | PM14A | D02 | 280 | 273 | 277 | 278 | 273 | 267 | |
| Cadmium Chloride | PM14A | D03 | | 26 | 26 | 23 | | 16 | |
| Cadmium Chloride | PM14A | D04 | 35 | 61 | 51 | 56 | 44 | 43 | |
| Iodoacetate | PM14A | D05 | 303 | 291 | 302 | 292 | 290 | 289 | 0.955 |
| Iodoacetate | PM14A | D06 | 311 | 289 | 307 | 307 | 283 | 293 | |
| Iodoacetate | PM14A | D07 | 243 | 276 | 266 | 237 | 286 | 283 | |
| Iodoacetate | PM14A | D08 | 20 | 19 | 17 | 16 | 19 | 15 | |
| Sodium Dichromate | PM14A | D09 | 290 | 286 | 298 | 294 | 288 | 285 | 0.262 |
| Sodium Dichromate | PM14A | D10 | 277 | 280 | 274 | 273 | 276 | 272 | |
| Sodium Dichromate | PM14A | D11 | 275 | 266 | 279 | 268 | 267 | 256 | |
| Sodium Dichromate | PM14A | D12 | 276 | 256 | 248 | | 235 | 215 | |
| Cefoxitin | PM14A | E01 | 288 | 285 | 289 | 285 | 288 | 285 | 0.429 |
| Cefoxitin | PM14A | E02 | 290 | 286 | 284 | 300 | 284 | 295 | |
| Cefoxitin | PM14A | E03 | 287 | 292 | 283 | 280 | 289 | 291 | |
| Cefoxitin | PM14A | E04 | | 290 | 298 | | 277 | 275 | |
| Nitrofurantoin | PM14A | E05 | 292 | 301 | 300 | 286 | 284 | 282 | 0.002 |
| Nitrofurantoin | PM14A | E06 | 297 | 296 | 295 | 302 | 295 | 303 | |
| Nitrofurantoin | PM14A | E07 | 303 | 298 | 298 | 291 | 289 | 283 | |
| Nitrofurantoin | PM14A | E08 | 298 | 298 | 298 | 291 | 289 | 292 | |
| Sodium Metaborate | PM14A | E09 | 275 | 289 | 283 | 285 | 275 | 286 | 0.965 |

| | | | | | | | | | |
|---|---|---|---|---|---|---|---|---|---|
| Sodium Metaborate | PM14A | E10 | 264 | 272 | 269 | 265 | 279 | 267 | |
| Sodium Metaborate | PM14A | E11 | 247 | 231 | 248 | 258 | 243 | 238 | |
| Sodium Metaborate | PM14A | E12 | 19 | 21 | 21 | 23 | 22 | 22 | |
| Chloramphenicol | PM14A | F01 | 276 | 274 | 279 | 283 | 283 | 286 | 0.501 |
| Chloramphenicol | PM14A | F02 | 273 | 266 | 263 | 287 | 280 | 263 | |
| Chloramphenicol | PM14A | F03 | 272 | 249 | 271 | 250 | 264 | 276 | |
| Chloramphenicol | PM14A | F04 | 113 | 92 | | 20 | 31 | 17 | |
| Piperacillin | PM14A | F05 | 292 | 293 | 302 | 279 | 281 | 283 | 0.103 |
| Piperacillin | PM14A | F06 | 305 | 301 | 296 | 303 | 292 | 294 | |
| Piperacillin | PM14A | F07 | 286 | 289 | 276 | 291 | 282 | 296 | |
| Piperacillin | PM14A | F08 | 290 | 296 | 294 | 284 | 289 | 283 | |
| Sodium Metavanadate | PM14A | F09 | 17 | 17 | 14 | 11 | 14 | 11 | 0.595 |
| Sodium Metavanadate | PM14A | F10 | 17 | 15 | 13 | 19 | 18 | 20 | |
| Sodium Metavanadate | PM14A | F11 | 19 | 19 | 21 | 12 | 20 | 16 | |
| Sodium Metavanadate | PM14A | F12 | 27 | 20 | 25 | 23 | 25 | 23 | |
| Chelerythrine | PM14A | G01 | 283 | 289 | 288 | 304 | 303 | 305 | 0.018 |
| Chelerythrine | PM14A | G02 | 299 | 292 | 282 | 294 | 310 | 303 | |
| Chelerythrine | PM14A | G03 | 297 | 296 | 301 | 296 | 293 | 295 | |
| Chelerythrine | PM14A | G04 | 298 | 302 | 302 | 304 | 303 | 298 | |
| Carbenicillin | PM14A | G05 | | | | | | | 0.001 |
| Carbenicillin | PM14A | G06 | 20 | 19 | 18 | 13 | | 11 | |
| Carbenicillin | PM14A | G07 | 18 | 17 | 17 | 11 | 11 | 11 | |
| Carbenicillin | PM14A | G08 | 12 | 14 | 11 | 11 | 13 | 11 | |
| Sodium Nitrite | PM14A | G09 | 282 | 288 | 278 | 294 | 283 | 283 | 0.794 |
| Sodium Nitrite | PM14A | G10 | 265 | 271 | 273 | 275 | 277 | 271 | |
| Sodium Nitrite | PM14A | G11 | 240 | 231 | 240 | 264 | 255 | 281 | |
| Sodium Nitrite | PM14A | G12 | 34 | 34 | 34 | 45 | 43 | 36 | |
| EGTA | PM14A | H01 | 297 | 295 | 296 | 290 | 283 | 292 | 0.868 |
| EGTA | PM14A | H02 | 287 | 282 | 291 | 289 | 288 | 279 | |
| EGTA | PM14A | H03 | 284 | 289 | 291 | 291 | 292 | 290 | |
| EGTA | PM14A | H04 | 284 | 283 | 286 | 296 | 288 | 291 | |
| Promethazine | PM14A | H05 | 283 | 287 | 288 | 299 | 302 | 286 | 0.958 |
| Promethazine | PM14A | H06 | 293 | 291 | 289 | 291 | 296 | 305 | |
| Promethazine | PM14A | H07 | 305 | 311 | 305 | 299 | 304 | 300 | |
| Promethazine | PM14A | H08 | 18 | 19 | 21 | 14 | 27 | 20 | |
| Sodium Orthovanadate | PM14A | H09 | 21 | 19 | 22 | 11 | 25 | 13 | 0.363 |

| | | | | | | | | | |
|---|---|---|---|---|---|---|---|---|---|
| Sodium Orthovanadate | PM14A | H10 | 19 | 24 | 18 | 13 | 25 | 17 | |
| Sodium Orthovanadate | PM14A | H11 | 27 | 30 | 25 | 17 | 28 | 20 | |
| Sodium Orthovanadate | PM14A | H12 | 32 | 37 | 31 | 26 | 35 | 40 | |
| Procaine | PM15B | A01 | 291 | 292 | 302 | 308 | 308 | 305 | 0.771 |
| Procaine | PM15B | A02 | 295 | 299 | 300 | 305 | 303 | 298 | |
| Procaine | PM15B | A03 | 292 | 294 | 297 | 294 | 287 | 285 | |
| Procaine | PM15B | A04 | 272 | 251 | 257 | 263 | 265 | 248 | |
| Guanidine hydrochloride | PM15B | A05 | 285 | 290 | 291 | 297 | 292 | 291 | 0.966 |
| Guanidine hydrochloride | PM15B | A06 | 294 | 289 | 291 | 293 | 292 | 294 | |
| Guanidine hydrochloride | PM15B | A07 | 277 | 285 | 287 | 286 | 284 | 277 | |
| Guanidine hydrochloride | PM15B | A08 | 20 | 15 | 13 | 19 | 20 | 18 | |
| Cefmetazole | PM15B | A09 | 281 | 275 | 284 | 289 | 287 | 285 | 0.191 |
| Cefmetazole | PM15B | A10 | 284 | 294 | 296 | 297 | 304 | 295 | |
| Cefmetazole | PM15B | A11 | 290 | 292 | 299 | 297 | 288 | 306 | |
| Cefmetazole | PM15B | A12 | 297 | 286 | 300 | | 288 | 299 | |
| D-Cycloserine | PM15B | B01 | 288 | 302 | 292 | 288 | 285 | 280 | 0.625 |
| D-Cycloserine | PM15B | B02 | 293 | 294 | 291 | 287 | 288 | 285 | |
| D-Cycloserine | PM15B | B03 | 294 | 286 | 293 | 290 | 288 | 287 | |
| D-Cycloserine | PM15B | B04 | | 252 | 233 | 290 | 287 | 283 | |
| EDTA | PM15B | B05 | 294 | 288 | 293 | 295 | 290 | 294 | 0.951 |
| EDTA | PM15B | B06 | 302 | 300 | 307 | 304 | 293 | 296 | |
| EDTA | PM15B | B07 | 302 | 301 | 312 | 301 | 299 | 293 | |
| EDTA | PM15B | B08 | | 24 | 26 | 28 | | 24 | |
| quinaldine | PM15B | B09 | 304 | 305 | 303 | 288 | 291 | 281 | 0.0000042 |
| quinaldine | PM15B | B10 | 296 | 299 | 306 | 292 | 289 | 287 | |
| quinaldine | PM15B | B11 | 292 | 297 | 294 | 276 | 272 | 280 | |
| quinaldine | PM15B | B12 | 289 | 297 | 293 | 283 | 275 | 280 | |
| 5,7-Dichloro-8-hydroxyquinoline | PM15B | C01 | 288 | 307 | 296 | 286 | 278 | 286 | 0.257 |
| 5,7-Dichloro-8-hydroxyquinoline | PM15B | C02 | 296 | 300 | 299 | 280 | 282 | 283 | |
| 5,7-Dichloro-8-hydroxyquinoline | PM15B | C03 | 295 | 298 | 300 | 295 | 291 | 288 | |
| 5,7-Dichloro-8-hydroxyquinoline | PM15B | C04 | | 77 | 60 | 307 | 301 | | |
| Fusidic acid | PM15B | C05 | 284 | 280 | 287 | 288 | 287 | 280 | 0.809 |
| Fusidic acid | PM15B | C06 | 302 | 293 | 294 | 293 | 294 | 284 | |
| Fusidic acid | PM15B | C07 | 294 | 293 | 299 | 296 | 289 | 287 | |
| Fusidic acid | PM15B | C08 | 252 | 229 | 231 | 255 | 266 | 245 | |
| 1,10-Phenanthroline | PM15B | C09 | 309 | 307 | 307 | 298 | 295 | 290 | 0.904 |

| | | | | | | | | | |
|---|---|---|---|---|---|---|---|---|---|
| 1,10-Phenanthroline | PM15B | C10 | 293 | 284 | 287 | 277 | 279 | 266 | |
| 1,10-Phenanthroline | PM15B | C11 | 34 | 44 | 37 | 33 | 37 | 60 | |
| 1,10-Phenanthroline | PM15B | C12 | 30 | 40 | 33 | 31 | 34 | 25 | |
| Phleomycin | PM15B | D01 | 291 | 291 | 296 | 294 | 289 | 285 | 0.896 |
| Phleomycin | PM15B | D02 | 286 | | 301 | 279 | 287 | 280 | |
| Phleomycin | PM15B | D03 | 29 | 22 | 22 | 23 | 23 | 18 | |
| Phleomycin | PM15B | D04 | 23 | 21 | 20 | 22 | 20 | 20 | |
| Domiphen bromide | PM15B | D05 | 283 | 287 | 292 | 288 | 291 | 287 | 0.972 |
| Domiphen bromide | PM15B | D06 | 296 | 300 | 295 | 298 | 296 | 293 | |
| Domiphen bromide | PM15B | D07 | 296 | 304 | 306 | 300 | 306 | 291 | |
| Domiphen bromide | PM15B | D08 | 25 | 22 | 20 | 20 | 18 | 16 | |
| Nordihydroguaiaretic acid | PM15B | D09 | 293 | 281 | 298 | 291 | 299 | 284 | 0.301 |
| Nordihydroguaiaretic acid | PM15B | D10 | 296 | 302 | 300 | 289 | 292 | 293 | |
| Nordihydroguaiaretic acid | PM15B | D11 | 306 | 306 | 312 | 293 | 297 | 295 | |
| Nordihydroguaiaretic acid | PM15B | D12 | 318 | 316 | 319 | 315 | 324 | 314 | |
| Alexidine | PM15B | E01 | 282 | 282 | 284 | 286 | 281 | 278 | 0.876 |
| Alexidine | PM15B | E02 | 289 | 287 | 280 | 279 | 273 | 281 | |
| Alexidine | PM15B | E03 | 249 | 241 | 284 | 296 | 297 | 289 | |
| Alexidine | PM15B | E04 | 15 | 18 | 14 | 18 | 27 | 12 | |
| Nitrofurazone | PM15B | E05 | 286 | 304 | 301 | 295 | 302 | 281 | 0.226 |
| Nitrofurazone | PM15B | E06 | 301 | 300 | 300 | 302 | 302 | 308 | |
| Nitrofurazone | PM15B | E07 | 300 | 301 | 307 | 295 | 297 | 289 | |
| Nitrofurazone | PM15B | E08 | 279 | 289 | 291 | 278 | 276 | 270 | |
| Methyl viologen | PM15B | E09 | 283 | 288 | 273 | 278 | 279 | 276 | 0.847 |
| Methyl viologen | PM15B | E10 | 275 | 286 | 292 | 293 | 293 | 290 | |
| Methyl viologen | PM15B | E11 | 272 | 275 | 273 | 274 | 273 | 278 | |
| Methyl viologen | PM15B | E12 | 282 | 274 | 279 | 280 | 275 | 270 | |
| 3, 4-Dimethoxybenzyl alcohol | PM15B | F01 | 285 | 292 | 288 | 293 | 286 | 291 | 0.862 |
| 3, 4-Dimethoxybenzyl alcohol | PM15B | F02 | 267 | 265 | 262 | 269 | 268 | 266 | |
| 3, 4-Dimethoxybenzyl alcohol | PM15B | F03 | 244 | 231 | 251 | 263 | 240 | 253 | |
| 3, 4-Dimethoxybenzyl alcohol | PM15B | F04 | 22 | 17 | 19 | 67 | 21 | 21 | |
| Oleandomycin | PM15B | F05 | 287 | 285 | 291 | 294 | 284 | 278 | 0.771 |
| Oleandomycin | PM15B | F06 | 302 | 304 | 305 | 297 | 293 | 298 | |
| Oleandomycin | PM15B | F07 | 280 | 274 | 282 | 281 | 284 | 280 | |
| Oleandomycin | PM15B | F08 | 262 | 277 | 279 | 285 | 284 | 285 | |
| Puromycin | PM15B | F09 | 280 | 268 | 292 | 287 | 285 | 277 | 0.467 |

| | | | | | | | | | |
|---|---|---|---|---|---|---|---|---|---|
| Puromycin | PM15B | F10 | 277 | 280 | 267 | 276 | 279 | 273 | |
| Puromycin | PM15B | F11 | 279 | 273 | 281 | 294 | 294 | 290 | |
| Puromycin | PM15B | F12 | 314 | 313 | 309 | 313 | 315 | 307 | |
| CCCP | PM15B | G01 | 280 | 296 | 293 | 311 | 297 | 306 | 0.853 |
| CCCP | PM15B | G02 | 295 | 298 | 297 | 299 | 291 | 307 | |
| CCCP | PM15B | G03 | | 178 | 196 | 196 | 189 | | |
| CCCP | PM15B | G04 | 132 | 158 | 174 | 150 | 169 | 143 | |
| Sodium azide | PM15B | G05 | 292 | 309 | 296 | 292 | 293 | 293 | 0.928 |
| Sodium azide | PM15B | G06 | 292 | 281 | 291 | 287 | 271 | 285 | |
| Sodium azide | PM15B | G07 | 22 | 23 | 20 | 11 | 14 | 11 | |
| Sodium azide | PM15B | G08 | 17 | 19 | 18 | 22 | 23 | 14 | |
| Menadione | PM15B | G09 | 304 | 295 | 299 | 309 | 307 | 304 | 0.005 |
| Menadione | PM15B | G10 | 305 | 304 | 308 | 308 | 312 | 307 | |
| Menadione | PM15B | G11 | 300 | 292 | 300 | 312 | 313 | 311 | |
| Menadione | PM15B | G12 | 311 | 305 | 309 | 300 | 311 | 316 | |
| 2-Nitroimidazole | PM15B | H01 | 291 | 287 | 300 | 288 | 283 | 283 | 0.466 |
| 2-Nitroimidazole | PM15B | H02 | 291 | 286 | 291 | 290 | 287 | 288 | |
| 2-Nitroimidazole | PM15B | H03 | 305 | 297 | 306 | 313 | 308 | 306 | |
| 2-Nitroimidazole | PM15B | H04 | 112 | | 67 | 210 | 247 | 221 | |
| Hydroxyurea | PM15B | H05 | 295 | 295 | 299 | 300 | 295 | 293 | 0.757 |
| Hydroxyurea | PM15B | H06 | 297 | 304 | 301 | 313 | 315 | 295 | |
| Hydroxyurea | PM15B | H07 | 290 | 279 | 273 | 282 | 280 | 280 | |
| Hydroxyurea | PM15B | H08 | | 26 | 22 | 13 | 15 | 63 | |
| Zinc chloride | PM15B | H09 | 293 | 288 | 290 | 291 | 284 | 280 | 0.207 |
| Zinc chloride | PM15B | H10 | 298 | 291 | 295 | 281 | 288 | 280 | |
| Zinc chloride | PM15B | H11 | 293 | 290 | 298 | 293 | 284 | 284 | |
| Zinc chloride | PM15B | H12 | 297 | 300 | 307 | 308 | 306 | 307 | |
| Cefotaxime | PM16A | A01 | 305 | 302 | 311 | 333 | 328 | 316 | 0.941 |
| Cefotaxime | PM16A | A02 | 280 | 316 | 305 | 298 | 285 | 292 | |
| Cefotaxime | PM16A | A03 | 36 | 20 | 16 | 26 | 26 | 24 | |
| Cefotaxime | PM16A | A04 | 25 | 13 | 18 | 24 | 29 | 20 | |
| Phosphomycin | PM16A | A05 | 308 | 305 | 313 | 308 | 304 | 298 | 0.007 |
| Phosphomycin | PM16A | A06 | 311 | 313 | 317 | 307 | 301 | 299 | |
| Phosphomycin | PM16A | A07 | 308 | 308 | 312 | 308 | 305 | 303 | |
| Phosphomycin | PM16A | A08 | 303 | 299 | 305 | 295 | 307 | 295 | |
| 5-Chloro-7-Iodo-8-Hydroxyquinoline | PM16A | A09 | 303 | 302 | 306 | 297 | 302 | 293 | 0.098 |

| | | | | | | | | | |
|---|---|---|---|---|---|---|---|---|---|
| 5-Chloro-7-Iodo-8-Hydroxyquinoline | PM16A | A10 | 280 | 267 | 269 | 302 | 291 | 307 | |
| 5-Chloro-7-Iodo-8-Hydroxyquinoline | PM16A | A11 | | 264 | 275 | 283 | 279 | 276 | |
| 5-Chloro-7-Iodo-8-Hydroxyquinoline | PM16A | A12 | 266 | | 276 | | 287 | 285 | |
| Norfloxacin | PM16A | B01 | 307 | 308 | 307 | 298 | 293 | 288 | 0.000 |
| Norfloxacin | PM16A | B02 | 300 | 303 | 303 | 292 | 292 | 286 | |
| Norfloxacin | PM16A | B03 | 304 | 301 | 309 | 288 | 295 | 285 | |
| Norfloxacin | PM16A | B04 | 306 | 302 | 310 | 297 | 296 | 292 | |
| Sulfanilamide | PM16A | B05 | 315 | 311 | 315 | 306 | 303 | 297 | 0.000 |
| Sulfanilamide | PM16A | B06 | 316 | 312 | 314 | 311 | 302 | 303 | |
| Sulfanilamide | PM16A | B07 | 313 | 309 | 314 | 308 | 298 | 300 | |
| Sulfanilamide | PM16A | B08 | 308 | 298 | 306 | 286 | 293 | 291 | |
| Trimethoprim | PM16A | B09 | 300 | 299 | 301 | 284 | 289 | 283 | 0.595 |
| Trimethoprim | PM16A | B10 | 300 | 292 | 298 | 283 | 288 | 283 | |
| Trimethoprim | PM16A | B11 | 288 | 271 | 279 | 282 | 265 | 259 | |
| Trimethoprim | PM16A | B12 | | 19 | 22 | 32 | 36 | 26 | |
| Dichlofluanid | PM16A | C01 | 302 | 303 | 299 | 309 | 290 | 299 | 0.096 |
| Dichlofluanid | PM16A | C02 | 296 | 303 | 304 | 294 | 294 | 293 | |
| Dichlofluanid | PM16A | C03 | 308 | 296 | 310 | 312 | 289 | 289 | |
| Dichlofluanid | PM16A | C04 | | | | | | | |
| Protamine Sulfate | PM16A | C05 | 302 | 297 | 306 | 292 | 303 | 292 | 0.983 |
| Protamine Sulfate | PM16A | C06 | 301 | 295 | 292 | 299 | 299 | 293 | |
| Protamine Sulfate | PM16A | C07 | 48 | 22 | 28 | 26 | 57 | 22 | |
| Protamine Sulfate | PM16A | C08 | 26 | 21 | 22 | 22 | 24 | 16 | |
| Cetylpyridinium Chloride | PM16A | C09 | 301 | 308 | 309 | 289 | 291 | 282 | 0.000 |
| Cetylpyridinium Chloride | PM16A | C10 | 295 | 291 | 297 | 272 | 281 | 280 | |
| Cetylpyridinium Chloride | PM16A | C11 | 290 | 293 | 280 | 264 | 261 | 249 | |
| Cetylpyridinium Chloride | PM16A | C12 | 293 | 295 | 292 | 279 | 275 | 272 | |
| 1-Chloro-2,4-Dinitrobenzene | PM16A | D01 | 315 | 315 | 312 | 313 | 316 | 315 | 0.905 |
| 1-Chloro-2,4-Dinitrobenzene | PM16A | D02 | 305 | 299 | 304 | 298 | 300 | 292 | |
| 1-Chloro-2,4-Dinitrobenzene | PM16A | D03 | 308 | 296 | 314 | 307 | 306 | 297 | |
| 1-Chloro-2,4-Dinitrobenzene | PM16A | D04 | 93 | 83 | 81 | 70 | 84 | 66 | |
| Diamide | PM16A | D05 | 313 | 310 | 314 | 308 | 313 | 303 | 0.000 |
| Diamide | PM16A | D06 | 313 | 309 | 310 | 307 | 302 | 297 | |
| Diamide | PM16A | D07 | 308 | 306 | 314 | 302 | 296 | 301 | |

| | | | | | | | | | |
|---|---|---|---|---|---|---|---|---|---|
| Diamide | PM16A | D08 | 313 | 315 | 320 | 309 | 290 | 302 | |
| Cinoxacin | PM16A | D09 | 300 | 293 | 300 | 288 | 294 | 290 | 0.761 |
| Cinoxacin | PM16A | D10 | 290 | 285 | 275 | 273 | 273 | 266 | |
| Cinoxacin | PM16A | D11 | | 18 | 21 | 22 | 23 | 20 | |
| Cinoxacin | PM16A | D12 | 28 | 26 | 28 | 23 | 27 | 23 | |
| Streptomycin | PM16A | E01 | 293 | 312 | 296 | 307 | 310 | 310 | 0.020 |
| Streptomycin | PM16A | E02 | 298 | 298 | 307 | 310 | 304 | 300 | |
| Streptomycin | PM16A | E03 | 299 | 299 | 306 | 311 | 313 | 300 | |
| Streptomycin | PM16A | E04 | 307 | 304 | 309 | 314 | 308 | 306 | |
| 5-Azacytidine | PM16A | E05 | 312 | 310 | 314 | 303 | 307 | 299 | 0.029 |
| 5-Azacytidine | PM16A | E06 | 306 | 305 | 309 | 306 | 307 | 292 | |
| 5-Azacytidine | PM16A | E07 | 308 | 299 | 307 | 291 | 295 | 299 | |
| 5-Azacytidine | PM16A | E08 | 296 | 299 | 305 | 308 | 299 | 299 | |
| Rifamycin SV | PM16A | E09 | 299 | 302 | 307 | 293 | 294 | 290 | 0.085 |
| Rifamycin SV | PM16A | E10 | 299 | 299 | 298 | 304 | 286 | 291 | |
| Rifamycin SV | PM16A | E11 | 307 | 309 | 313 | 304 | 306 | 301 | |
| Rifamycin SV | PM16A | E12 | 317 | 316 | 319 | 317 | 313 | 308 | |
| Potassium Tellurite | PM16A | F01 | 301 | 293 | 301 | 303 | 301 | 299 | 0.817 |
| Potassium Tellurite | PM16A | F02 | 290 | 295 | 299 | 316 | 318 | 310 | |
| Potassium Tellurite | PM16A | F03 | 310 | | 273 | 286 | | 287 | |
| Potassium Tellurite | PM16A | F04 | 133 | 126 | | | 129 | 146 | |
| Sodium Selenite | PM16A | F05 | 306 | 287 | 299 | 307 | 304 | 291 | 0.303 |
| Sodium Selenite | PM16A | F06 | 308 | 301 | 304 | 305 | 299 | 297 | |
| Sodium Selenite | PM16A | F07 | 301 | 302 | 310 | 306 | 310 | 305 | |
| Sodium Selenite | PM16A | F08 | 302 | 308 | 306 | 324 | 315 | 309 | |
| Aluminum Sulfate | PM16A | F09 | 301 | 298 | 308 | 304 | 310 | 304 | 0.126 |
| Aluminum Sulfate | PM16A | F10 | 287 | 283 | 287 | 285 | 288 | 278 | |
| Aluminum Sulfate | PM16A | F11 | 282 | 274 | 281 | 300 | 299 | 299 | |
| Aluminum Sulfate | PM16A | F12 | 289 | 288 | 297 | 297 | 294 | 290 | |
| Chromium Chloride | PM16A | G01 | 299 | 285 | 290 | 297 | 297 | 307 | 0.021 |
| Chromium Chloride | PM16A | G02 | 295 | 282 | 287 | 299 | 308 | 287 | |
| Chromium Chloride | PM16A | G03 | 301 | 296 | 296 | 304 | 299 | 296 | |
| Chromium Chloride | PM16A | G04 | 296 | 301 | 304 | 303 | 307 | 310 | |
| Ferric Chloride | PM16A | G05 | 319 | 313 | 314 | 317 | 320 | 290 | 0.641 |
| Ferric Chloride | PM16A | G06 | 314 | 313 | 318 | 314 | 321 | 321 | |
| Ferric Chloride | PM16A | G07 | 316 | 304 | 317 | 313 | 310 | 308 | |

| | | | | | | | | | |
|---|---|---|---|---|---|---|---|---|---|
| Ferric Chloride | PM16A | G08 | 307 | 291 | 287 | 314 | 298 | 310 | |
| L-Glutamic-g-Hydroxamate | PM16A | G09 | 303 | 299 | 309 | 304 | 304 | 298 | 0.192 |
| L-Glutamic-g-Hydroxamate | PM16A | G10 | 294 | 294 | 297 | 299 | 294 | 296 | |
| L-Glutamic-g-Hydroxamate | PM16A | G11 | 269 | 285 | 275 | 293 | 289 | 293 | |
| L-Glutamic-g-Hydroxamate | PM16A | G12 | 291 | 289 | 293 | 296 | 295 | 293 | |
| Glycine Hydroxamate | PM16A | H01 | 293 | 286 | 299 | 286 | 287 | 286 | 0.850 |
| Glycine Hydroxamate | PM16A | H02 | 289 | 285 | 289 | 296 | 287 | 288 | |
| Glycine Hydroxamate | PM16A | H03 | 291 | 290 | 292 | 295 | 289 | 290 | |
| Glycine Hydroxamate | PM16A | H04 | 295 | 298 | 301 | 310 | 300 | 300 | |
| Chloroxylenol | PM16A | H05 | 323 | 307 | 325 | 320 | 325 | 318 | 0.135 |
| Chloroxylenol | PM16A | H06 | 322 | 328 | 323 | 325 | 328 | 322 | |
| Chloroxylenol | PM16A | H07 | 326 | 301 | 315 | 324 | 324 | 319 | |
| Chloroxylenol | PM16A | H08 | 320 | 317 | 276 | 319 | 323 | 317 | |
| Sorbic Acid | PM16A | H09 | 321 | 317 | 322 | 310 | 308 | 305 | 0.000 |
| Sorbic Acid | PM16A | H10 | 326 | 321 | 319 | 304 | 304 | 304 | |
| Sorbic Acid | PM16A | H11 | 316 | 306 | 309 | 313 | 311 | 303 | |
| Sorbic Acid | PM16A | H12 | 314 | 313 | 311 | 311 | 294 | 313 | |
| D-Serine | PM17A | A01 | 292 | 284 | 301 | 297 | 303 | 301 | 0.964 |
| D-Serine | PM17A | A02 | 310 | 309 | 308 | 308 | 311 | 312 | |
| D-Serine | PM17A | A03 | 313 | 310 | 317 | 314 | 292 | 309 | |
| D-Serine | PM17A | A04 | 16 | 16 | 15 | 34 | 21 | 18 | |
| b-Chloro-L-Alanine | PM17A | A05 | 289 | 287 | 292 | 294 | 288 | 281 | 0.808 |
| b-Chloro-L-Alanine | PM17A | A06 | 300 | 297 | 297 | 301 | 296 | 298 | |
| b-Chloro-L-Alanine | PM17A | A07 | 297 | 294 | 299 | 303 | 299 | 295 | |
| b-Chloro-L-Alanine | PM17A | A08 | 305 | 299 | 302 | 304 | 296 | 296 | |
| Thiosalicylate | PM17A | A09 | 295 | 295 | 296 | 292 | 296 | 292 | 0.038 |
| Thiosalicylate | PM17A | A10 | 292 | 297 | 299 | 297 | 303 | 305 | |
| Thiosalicylate | PM17A | A11 | 296 | 297 | 299 | 303 | 308 | 301 | |
| Thiosalicylate | PM17A | A12 | 300 | 298 | 298 | 303 | 305 | 300 | |
| Salicylate | PM17A | B01 | 284 | 287 | 284 | 286 | 275 | 276 | 0.879 |
| Salicylate | PM17A | B02 | 257 | 258 | 253 | 271 | 255 | 253 | |
| Salicylate | PM17A | B03 | 212 | | 204 | 223 | 237 | 215 | |
| Salicylate | PM17A | B04 | 13 | 17 | 13 | 26 | 20 | 20 | |
| Hygromycin B | PM17A | B05 | 307 | 302 | 302 | 289 | 296 | 295 | 0.954 |
| Hygromycin B | PM17A | B06 | 304 | 301 | 304 | 297 | 300 | 300 | |
| Hygromycin B | PM17A | B07 | | | | | | | |

| | | | | | | | | | |
|---|---|---|---|---|---|---|---|---|---|
| Hygromycin B | PM17A | B08 | 21 | 18 | 20 | 21 | 24 | 22 | |
| Ethionamide | PM17A | B09 | 299 | 294 | 306 | 284 | 291 | 279 | 0.012 |
| Ethionamide | PM17A | B10 | 298 | 300 | 311 | 279 | 285 | 290 | |
| Ethionamide | PM17A | B11 | 272 | 287 | 272 | 262 | 255 | 256 | |
| Ethionamide | PM17A | B12 | 275 | 285 | 265 | 270 | 259 | 264 | |
| 4-Aminopyridine | PM17A | C01 | 298 | 309 | 293 | 295 | 290 | 292 | 0.912 |
| 4-Aminopyridine | PM17A | C02 | 302 | 301 | 302 | 279 | 284 | 291 | |
| 4-Aminopyridine | PM17A | C03 | 303 | 303 | 302 | 290 | 289 | 292 | |
| 4-Aminopyridine | PM17A | C04 | 11 | 13 | 18 | 20 | 45 | 20 | |
| Sulfachloropyridazine | PM17A | C05 | 300 | 302 | 307 | 306 | 304 | 304 | 0.003 |
| Sulfachloropyridazine | PM17A | C06 | 307 | 307 | 309 | 303 | 305 | 302 | |
| Sulfachloropyridazine | PM17A | C07 | 302 | 302 | 306 | 296 | 288 | 288 | |
| Sulfachloropyridazine | PM17A | C08 | 309 | 302 | 310 | 289 | 294 | 294 | |
| Sulfamonomethoxine | PM17A | C09 | 301 | 300 | 308 | 286 | 287 | 286 | 0.000 |
| Sulfamonomethoxine | PM17A | C10 | 288 | 299 | 301 | 277 | 274 | 276 | |
| Sulfamonomethoxine | PM17A | C11 | 293 | 291 | 299 | 262 | 264 | 273 | |
| Sulfamonomethoxine | PM17A | C12 | 295 | 290 | 288 | 287 | 287 | 286 | |
| Oxycarboxin | PM17A | D01 | 304 | 300 | 299 | 299 | 298 | 301 | 0.924 |
| Oxycarboxin | PM17A | D02 | 278 | 272 | 289 | 284 | 284 | 277 | |
| Oxycarboxin | PM17A | D03 | 136 | | 155 | 185 | 182 | | |
| Oxycarboxin | PM17A | D04 | 26 | 23 | 18 | 15 | 15 | 16 | |
| Aminotriazole | PM17A | D05 | 302 | 300 | 309 | 297 | 293 | 292 | 0.479 |
| Aminotriazole | PM17A | D06 | 297 | 298 | 301 | 296 | 301 | 293 | |
| Aminotriazole | PM17A | D07 | 288 | 294 | 292 | 292 | 292 | 283 | |
| Aminotriazole | PM17A | D08 | 282 | 272 | 269 | 279 | 276 | 272 | |
| Chlorpromazine | PM17A | D09 | 289 | 295 | 298 | 282 | 283 | 277 | 0.076 |
| Chlorpromazine | PM17A | D10 | 293 | 297 | 300 | 286 | 282 | 296 | |
| Chlorpromazine | PM17A | D11 | 311 | 309 | 317 | 300 | 297 | 294 | |
| Chlorpromazine | PM17A | D12 | 317 | | 286 | 30 | | 27 | |
| Niaproof | PM17A | E01 | 302 | 300 | 302 | 305 | 301 | 300 | 0.407 |
| Niaproof | PM17A | E02 | 298 | 296 | 305 | 301 | 307 | 302 | |
| Niaproof | PM17A | E03 | 274 | 270 | 256 | 287 | 281 | 274 | |
| Niaproof | PM17A | E04 | 171 | 207 | 175 | 213 | 197 | | |
| Compound 48/80 | PM17A | E05 | 299 | 299 | 308 | 279 | 289 | 291 | 0.581 |
| Compound 48/80 | PM17A | E06 | 298 | 300 | 302 | 297 | 302 | 291 | |
| Compound 48/80 | PM17A | E07 | 275 | 291 | 292 | 293 | 283 | 279 | |

| | | | | | | | | | |
|---|---|---|---|---|---|---|---|---|---|
| Compound 48/80 | PM17A | E08 | | 60 | 21 | 23 | 25 | 26 | |
| Sodium Tungstate | PM17A | E09 | 283 | 276 | 281 | 282 | 271 | 272 | 0.169 |
| Sodium Tungstate | PM17A | E10 | 293 | 282 | 286 | 289 | 288 | 285 | |
| Sodium Tungstate | PM17A | E11 | 296 | 281 | 283 | 281 | 296 | 282 | |
| Sodium Tungstate | PM17A | E12 | 254 | | 297 | 244 | 246 | 251 | |
| Lithium Chloride | PM17A | F01 | 281 | 290 | 274 | 291 | 289 | 278 | 0.900 |
| Lithium Chloride | PM17A | F02 | 254 | 246 | 259 | 268 | 264 | 261 | |
| Lithium Chloride | PM17A | F03 | 225 | | 200 | | 229 | 190 | |
| Lithium Chloride | PM17A | F04 | 34 | 57 | 36 | 41 | 63 | 43 | |
| D,L-Methionine Hydroxamate | PM17A | F05 | 295 | 296 | 283 | 289 | 281 | 291 | 0.810 |
| D,L-Methionine Hydroxamate | PM17A | F06 | 290 | 296 | 297 | 298 | 302 | 293 | |
| D,L-Methionine Hydroxamate | PM17A | F07 | 16 | | 23 | 15 | 13 | 20 | |
| D,L-Methionine Hydroxamate | PM17A | F08 | 19 | 21 | 16 | 11 | 14 | 18 | |
| Tannic acid | PM17A | F09 | 312 | 308 | 308 | 312 | 319 | 313 | 0.157 |
| Tannic acid | PM17A | F10 | 304 | 305 | 307 | 306 | 310 | 309 | |
| Tannic acid | PM17A | F11 | 318 | 315 | 314 | 341 | 339 | 342 | |
| Tannic acid | PM17A | F12 | 339 | 342 | 338 | 344 | 343 | 340 | |
| Chlorambucil | PM17A | G01 | 289 | 273 | 282 | 308 | 302 | 288 | 0.119 |
| Chlorambucil | PM17A | G02 | 284 | 291 | 285 | 287 | 291 | 289 | |
| Chlorambucil | PM17A | G03 | 283 | 274 | 284 | 303 | 291 | 285 | |
| Chlorambucil | PM17A | G04 | 293 | 274 | 272 | 277 | 280 | 263 | |
| Cefamandole | PM17A | G05 | 304 | 311 | 313 | 296 | 296 | 300 | 0.458 |
| Cefamandole | PM17A | G06 | 298 | 293 | 294 | 308 | 296 | 298 | |
| Cefamandole | PM17A | G07 | 295 | 302 | 307 | 294 | 302 | 280 | |
| Cefamandole | PM17A | G08 | 300 | 298 | 303 | 307 | 310 | 304 | |
| Cetoperazone | PM17A | G09 | 298 | 300 | 285 | 290 | 295 | 291 | 0.713 |
| Cetoperazone | PM17A | G10 | 289 | 287 | 285 | 291 | 288 | 291 | |
| Cetoperazone | PM17A | G11 | 279 | 280 | 288 | 288 | 286 | 295 | |
| Cetoperazone | PM17A | G12 | 259 | 269 | 264 | 256 | 231 | 249 | |
| Cefsulodin | PM17A | H01 | 300 | 290 | 301 | 297 | 291 | 286 | 0.766 |
| Cefsulodin | PM17A | H02 | 299 | 290 | 290 | 293 | 295 | 290 | |
| Cefsulodin | PM17A | H03 | 296 | 292 | 295 | 297 | 300 | 297 | |
| Cefsulodin | PM17A | H04 | 301 | 296 | 301 | 307 | 306 | 300 | |
| Caffeine | PM17A | H05 | 311 | 303 | 297 | 310 | 308 | 308 | 0.941 |
| Caffeine | PM17A | H06 | 315 | 314 | 323 | 310 | 315 | 313 | |
| Caffeine | PM17A | H07 | 314 | 306 | 313 | 310 | 311 | 310 | |

| | | | | | | | | | |
|---|---|---|---|---|---|---|---|---|---|
| Caffeine | PM17A | H08 | | 102 | 100 | | 113 | 119 | |
| Phenylarsine Oxide | PM17A | H09 | 298 | 291 | 295 | 286 | 294 | 290 | 0.789 |
| Phenylarsine Oxide | PM17A | H10 | 304 | 295 | 304 | 287 | 280 | 291 | |
| Phenylarsine Oxide | PM17A | H11 | 291 | 296 | 299 | 296 | | 291 | |
| Phenylarsine Oxide | PM17A | H12 | 43 | 42 | 40 | 43 | 22 | 39 | |
| Ketoprofen | PM18C | A01 | 293 | 275 | 292 | 311 | 304 | 306 | 0.884 |
| Ketoprofen | PM18C | A02 | 310 | 313 | 315 | 310 | 313 | 303 | |
| Ketoprofen | PM18C | A03 | 299 | 296 | 296 | 295 | 298 | 292 | |
| Ketoprofen | PM18C | A04 | 195 | | 212 | 166 | | 164 | |
| Sodium pyrophosphate decahydrate | PM18C | A05 | 323 | 319 | 327 | 314 | 318 | 313 | 0.356 |
| Sodium pyrophosphate decahydrate | PM18C | A06 | 307 | 290 | 311 | 304 | 296 | 304 | |
| Sodium pyrophosphate decahydrate | PM18C | A07 | 310 | 308 | 300 | 311 | 301 | 307 | |
| Sodium pyrophosphate decahydrate | PM18C | A08 | 315 | 305 | 309 | 312 | 302 | 303 | |
| Thiamphenicol | PM18C | A09 | 291 | 281 | 288 | 293 | 289 | 285 | 0.705 |
| Thiamphenicol | PM18C | A10 | 279 | 268 | 271 | 290 | 289 | 287 | |
| Thiamphenicol | PM18C | A11 | 83 | 43 | 60 | 101 | 136 | 95 | |
| Thiamphenicol | PM18C | A12 | 26 | 21 | 28 | 37 | 30 | 34 | |
| Trifluorothymidine | PM18C | B01 | 290 | 289 | 288 | 285 | 277 | 279 | 0.006 |
| Trifluorothymidine | PM18C | B02 | 288 | 292 | 302 | 291 | 287 | 283 | |
| Trifluorothymidine | PM18C | B03 | 292 | 302 | 298 | 290 | 293 | 292 | |
| Trifluorothymidine | PM18C | B04 | 302 | 308 | 307 | 292 | 297 | 290 | |
| Pipemidic Acid | PM18C | B05 | 304 | 302 | 302 | 297 | 299 | 304 | 0.914 |
| Pipemidic Acid | PM18C | B06 | 308 | 307 | 312 | 311 | 306 | 302 | |
| Pipemidic Acid | PM18C | B07 | 278 | | 240 | 278 | 276 | 269 | |
| Pipemidic Acid | PM18C | B08 | 21 | 20 | 18 | 18 | 21 | 20 | |
| Azathioprine | PM18C | B09 | 285 | 286 | 291 | 289 | 287 | 282 | 0.051 |
| Azathioprine | PM18C | B10 | 286 | 279 | 288 | 286 | 282 | 277 | |
| Azathioprine | PM18C | B11 | 288 | 290 | 287 | 281 | 271 | 273 | |
| Azathioprine | PM18C | B12 | 301 | 296 | 306 | 300 | 286 | 291 | |
| Poly-L-lysine | PM18C | C01 | 281 | 281 | 287 | 275 | 278 | 275 | 0.004 |
| Poly-L-lysine | PM18C | C02 | 286 | 277 | 285 | 262 | 269 | 266 | |
| Poly-L-lysine | PM18C | C03 | 286 | 288 | 298 | 282 | 285 | 279 | |
| Poly-L-lysine | PM18C | C04 | | | | | | | |
| Sulfisoxazole | PM18C | C05 | 303 | 307 | 308 | 304 | 302 | 301 | 0.005 |

| | | | | | | | | | |
|---|---|---|---|---|---|---|---|---|---|
| Sulfisoxazole | PM18C | C06 | 312 | 310 | 314 | 313 | 312 | 301 | |
| Sulfisoxazole | PM18C | C07 | 312 | 309 | 312 | 306 | 310 | 301 | |
| Sulfisoxazole | PM18C | C08 | 320 | 322 | 320 | 310 | 309 | 296 | |
| Pentachlorophenol (PCP) | PM18C | C09 | 308 | 313 | 313 | 295 | 296 | 292 | 0.002 |
| Pentachlorophenol (PCP) | PM18C | C10 | 304 | 309 | 305 | 292 | 288 | 292 | |
| Pentachlorophenol (PCP) | PM18C | C11 | 289 | 285 | 288 | 272 | 272 | 267 | |
| Pentachlorophenol (PCP) | PM18C | C12 | 296 | 295 | 300 | 286 | 294 | 284 | |
| Sodium m-arsenite | PM18C | D01 | 19 | 24 | 18 | 24 | 21 | 23 | 0.063 |
| Sodium m-arsenite | PM18C | D02 | 18 | 20 | 23 | 15 | 16 | 15 | |
| Sodium m-arsenite | PM18C | D03 | 19 | 24 | 20 | 18 | 16 | 13 | |
| Sodium m-arsenite | PM18C | D04 | 15 | 18 | 20 | 18 | 16 | 11 | |
| Sodium bromate | PM18C | D05 | 291 | 292 | 298 | 289 | 292 | 299 | 0.376 |
| Sodium bromate | PM18C | D06 | 308 | 311 | 305 | 317 | 310 | 309 | |
| Sodium bromate | PM18C | D07 | 316 | 301 | 305 | 304 | 301 | 310 | |
| Sodium bromate | PM18C | D08 | 309 | 314 | 317 | 300 | 298 | 301 | |
| Lidocaine | PM18C | D09 | 329 | 326 | 324 | 323 | 320 | 317 | 0.919 |
| Lidocaine | PM18C | D10 | 327 | 321 | 324 | 321 | 320 | 317 | |
| Lidocaine | PM18C | D11 | 31 | 34 | 34 | 42 | 32 | 29 | |
| Lidocaine | PM18C | D12 | 29 | 43 | 70 | 32 | 35 | 29 | |
| Sodium metasilicate | PM18C | E01 | 304 | 310 | 307 | 310 | 315 | 308 | 0.578 |
| Sodium metasilicate | PM18C | E02 | 291 | 278 | 289 | 285 | 306 | 298 | |
| Sodium metasilicate | PM18C | E03 | 270 | 262 | 269 | 287 | 281 | 267 | |
| Sodium metasilicate | PM18C | E04 | 52 | 64 | 54 | 73 | | 52 | |
| Sodium periodate | PM18C | E05 | 294 | 297 | 300 | 301 | 300 | 296 | 0.956 |
| Sodium periodate | PM18C | E06 | 301 | 287 | 294 | 304 | 306 | 300 | |
| Sodium periodate | PM18C | E07 | 17 | 15 | 21 | 16 | 15 | 23 | |
| Sodium periodate | PM18C | E08 | 21 | 23 | 24 | 26 | 22 | 25 | |
| Antimony (III) chloride | PM18C | E09 | 296 | 297 | 307 | 299 | 294 | 288 | 0.937 |
| Antimony (III) chloride | PM18C | E10 | 305 | 301 | 299 | 318 | 309 | 309 | |
| Antimony (III) chloride | PM18C | E11 | 24 | 33 | 27 | 24 | 29 | 30 | |
| Antimony (III) chloride | PM18C | E12 | 19 | 20 | 21 | 62 | 19 | 24 | |
| Semicarbazide hydrochloride | PM18C | F01 | 272 | 263 | 272 | 280 | 272 | 277 | 0.327 |
| Semicarbazide hydrochloride | PM18C | F02 | 260 | 258 | 267 | 278 | 272 | 270 | |
| Semicarbazide hydrochloride | PM18C | F03 | 287 | 285 | 277 | 302 | 292 | 290 | |
| Semicarbazide hydrochloride | PM18C | F04 | 256 | | 261 | 56 | | 71 | |
| Tinidazole | PM18C | F05 | 291 | 280 | 288 | 302 | 290 | 294 | 0.074 |

| | | | | | | | | | |
|---|---|---|---|---|---|---|---|---|---|
| Tinidazole | PM18C | F06 | 286 | 298 | 310 | 304 | 305 | 301 | |
| Tinidazole | PM18C | F07 | 300 | 306 | 306 | 308 | 307 | 310 | |
| Tinidazole | PM18C | F08 | 307 | 307 | 313 | 332 | 317 | 330 | |
| Aztreonam | PM18C | F09 | 304 | 304 | 303 | 309 | 299 | 303 | 0.780 |
| Aztreonam | PM18C | F10 | 292 | 294 | 294 | 295 | 291 | 292 | |
| Aztreonam | PM18C | F11 | 278 | 279 | 275 | 298 | 287 | 280 | |
| Aztreonam | PM18C | F12 | | 25 | 24 | 25 | 48 | 26 | |
| Triclosan | PM18C | G01 | 273 | 265 | 272 | 296 | 285 | 286 | 0.132 |
| Triclosan | PM18C | G02 | 291 | 289 | 296 | 283 | 301 | 298 | |
| Triclosan | PM18C | G03 | 291 | 301 | 299 | 310 | 301 | 283 | |
| Triclosan | PM18C | G04 | 287 | 275 | 285 | 280 | 286 | 296 | |
| 3,5- Diamino-1,2,4-triazole (Guanazole) | PM18C | G05 | 294 | 308 | 300 | 315 | 307 | 293 | 0.032 |
| 3,5- Diamino-1,2,4-triazole (Guanazole) | PM18C | G06 | 307 | 298 | 299 | 311 | 312 | 313 | |
| 3,5- Diamino-1,2,4-triazole (Guanazole) | PM18C | G07 | 288 | 298 | 297 | 309 | 307 | 294 | |
| 3,5- Diamino-1,2,4-triazole (Guanazole) | PM18C | G08 | 286 | 275 | 275 | 290 | 293 | 295 | |
| Myricetin | PM18C | G09 | 292 | 310 | 310 | 317 | 313 | 308 | 0.024 |
| Myricetin | PM18C | G10 | 309 | 305 | 311 | 317 | 310 | 311 | |
| Myricetin | PM18C | G11 | 309 | 306 | 307 | 324 | 326 | 324 | |
| Myricetin | PM18C | G12 | 325 | 325 | 326 | 333 | 334 | 333 | |
| 5-Fluoro-5'-deoxyuridine | PM18C | H01 | 292 | 293 | 297 | 287 | 280 | 280 | 0.687 |
| 5-Fluoro-5'-deoxyuridine | PM18C | H02 | 295 | 287 | 291 | 285 | 287 | 290 | |
| 5-Fluoro-5'-deoxyuridine | PM18C | H03 | 284 | 284 | 282 | 293 | 287 | 290 | |
| 5-Fluoro-5'-deoxyuridine | PM18C | H04 | 286 | 278 | 284 | 297 | 285 | 281 | |
| 2- Phenylphenol | PM18C | H05 | 310 | 299 | 307 | 312 | 315 | 319 | 0.002 |
| 2- Phenylphenol | PM18C | H06 | 317 | 316 | 316 | 325 | 320 | 322 | |
| 2- Phenylphenol | PM18C | H07 | 314 | 315 | 319 | 320 | 322 | 319 | |
| 2- Phenylphenol | PM18C | H08 | 318 | 319 | 312 | 324 | 322 | 326 | |
| Plumbagin | PM18C | H09 | 301 | 301 | 301 | 299 | 300 | 298 | 0.281 |
| Plumbagin | PM18C | H10 | 316 | 314 | 311 | 301 | 297 | 306 | |
| Plumbagin | PM18C | H11 | 313 | 312 | 317 | 315 | 311 | 308 | |
| Plumbagin | PM18C | H12 | 321 | 316 | 319 | 323 | 320 | 319 | |
| Josamycin | PM19 | A01 | 295 | 283 | 300 | 306 | 292 | 295 | 0.434 |
| Josamycin | PM19 | A02 | 301 | 299 | 303 | 302 | 304 | 297 | |
| Josamycin | PM19 | A03 | 278 | 304 | 307 | 306 | 302 | 288 | |

| | | | | | | | | | |
|---|---|---|---|---|---|---|---|---|---|
| Josamycin | PM19 | A04 | 245 | 244 | 243 | 257 | 263 | 274 | |
| Gallic Acid | PM19 | A05 | 342 | 336 | 340 | 335 | 334 | 335 | 0.067 |
| Gallic Acid | PM19 | A06 | 362 | 360 | 361 | 352 | 352 | 349 | |
| Gallic Acid | PM19 | A07 | 366 | 363 | 365 | 356 | 356 | 358 | |
| Gallic Acid | PM19 | A08 | 364 | 363 | 364 | 354 | 354 | 356 | |
| Coumarin | PM19 | A09 | 293 | 296 | 294 | 298 | 300 | 297 | 0.815 |
| Coumarin | PM19 | A10 | 293 | 292 | 301 | 305 | 309 | 308 | |
| Coumarin | PM19 | A11 | 272 | 291 | 289 | 294 | 300 | 297 | |
| Coumarin | PM19 | A12 | 68 | 56 | 37 | 69 | 63 | 66 | |
| Chloride | PM19 | B01 | 291 | 291 | 295 | 289 | 280 | 278 | 0.979 |
| Chloride | PM19 | B02 | 298 | 302 | 302 | 295 | 298 | 282 | |
| Chloride | PM19 | B03 | 290 | 294 | 296 | 298 | 299 | 290 | |
| Chloride | PM19 | B04 | 17 | 16 | 15 | 32 | 29 | 53 | |
| Harmane | PM19 | B05 | 309 | 313 | 314 | 304 | 302 | 291 | 0.906 |
| Harmane | PM19 | B06 | 318 | 317 | 319 | 312 | 314 | 301 | |
| Harmane | PM19 | B07 | 295 | 292 | 291 | 293 | 295 | 288 | |
| Harmane | PM19 | B08 | | 50 | 41 | 50 | | 51 | |
| 2,4-Dintrophenol | PM19 | B09 | 307 | 309 | 312 | 294 | 299 | 288 | 0.856 |
| 2,4-Dintrophenol | PM19 | B10 | 302 | 310 | 303 | 300 | 299 | 296 | |
| 2,4-Dintrophenol | PM19 | B11 | 298 | 305 | 306 | 292 | 288 | 289 | |
| 2,4-Dintrophenol | PM19 | B12 | 134 | 132 | 141 | 155 | 151 | 144 | |
| Chlorhexidine | PM19 | C01 | 287 | 295 | 293 | 290 | 293 | 283 | 0.001 |
| Chlorhexidine | PM19 | C02 | 301 | 297 | 300 | 281 | 281 | 275 | |
| Chlorhexidine | PM19 | C03 | 282 | 300 | 301 | 293 | 298 | 290 | |
| Chlorhexidine | PM19 | C04 | 298 | 297 | 302 | 281 | 282 | 272 | |
| Umbelliferone | PM19 | C05 | 302 | 307 | 312 | 303 | 300 | 289 | 0.000 |
| Umbelliferone | PM19 | C06 | 301 | 314 | 314 | 295 | 300 | 273 | |
| Umbelliferone | PM19 | C07 | 306 | 312 | 308 | 287 | 295 | 287 | |
| Umbelliferone | PM19 | C08 | 311 | 299 | 302 | 298 | 296 | 285 | |
| Cinnamic Acid | PM19 | C09 | 303 | 304 | 304 | 288 | 292 | 282 | 0.015 |
| Cinnamic Acid | PM19 | C10 | 300 | 301 | 297 | 274 | 274 | 274 | |
| Cinnamic Acid | PM19 | C11 | 289 | 290 | 289 | 269 | 270 | 268 | |
| Cinnamic Acid | PM19 | C12 | | 251 | 254 | 263 | 259 | 242 | |
| Disulphiram | PM19 | D01 | 295 | 294 | 297 | 299 | 296 | 289 | 0.031 |
| Disulphiram | PM19 | D02 | 294 | 298 | 301 | 292 | 289 | 284 | |
| Disulphiram | PM19 | D03 | 301 | 303 | 303 | 302 | 298 | 290 | |

| | | | | | | | | | |
|---|---|---|---|---|---|---|---|---|---|
| Disulphiram | PM19 | D04 | 298 | 302 | 304 | 301 | 299 | 297 | |
| Iodonitro Tetrazolium Violet | PM19 | D05 | 344 | 343 | 343 | 341 | 343 | 339 | 0.754 |
| Iodonitro Tetrazolium Violet | PM19 | D06 | 358 | 355 | 358 | 354 | 355 | 354 | |
| Iodonitro Tetrazolium Violet | PM19 | D07 | 346 | 331 | 327 | 333 | 354 | 325 | |
| Iodonitro Tetrazolium Violet | PM19 | D08 | 340 | 322 | 330 | 327 | 332 | 321 | |
| Phenyl-Methyl-Sulfonyl-Fluoride (PMSF) | PM19 | D09 | 305 | 307 | 310 | 301 | 302 | 298 | 0.006 |
| Phenyl-Methyl-Sulfonyl-Fluoride (PMSF) | PM19 | D10 | 311 | 307 | 308 | 302 | 303 | 295 | |
| Phenyl-Methyl-Sulfonyl-Fluoride (PMSF) | PM19 | D11 | 307 | 312 | 308 | 297 | 294 | 289 | |
| Phenyl-Methyl-Sulfonyl-Fluoride (PMSF) | PM19 | D12 | 299 | 295 | | 299 | 314 | 294 | |
| FCCP | PM19 | E01 | 282 | 288 | 294 | 290 | 289 | 282 | 0.177 |
| FCCP | PM19 | E02 | 289 | 285 | 288 | 300 | 300 | 298 | |
| FCCP | PM19 | E03 | 295 | 305 | 304 | 311 | 316 | 307 | |
| FCCP | PM19 | E04 | 308 | 284 | 279 | 286 | 299 | 291 | |
| D,L-Thioctic Acid | PM19 | E05 | 302 | 306 | 308 | 307 | 305 | 301 | 0.764 |
| D,L-Thioctic Acid | PM19 | E06 | 309 | 305 | 310 | 316 | 313 | 308 | |
| D,L-Thioctic Acid | PM19 | E07 | 268 | 290 | 301 | 301 | 291 | 295 | |
| D,L-Thioctic Acid | PM19 | E08 | | 25 | 31 | 31 | 34 | 24 | |
| Lawsone | PM19 | E09 | 320 | 318 | 319 | 318 | 318 | 314 | 0.924 |
| Lawsone | PM19 | E10 | 324 | 317 | 324 | 327 | 328 | 326 | |
| Lawsone | PM19 | E11 | 325 | 325 | 326 | 332 | 332 | 333 | |
| Lawsone | PM19 | E12 | 247 | 239 | 242 | 238 | 238 | 240 | |
| Phenethicillin | PM19 | F01 | 288 | 284 | 285 | 293 | 289 | 291 | 0.674 |
| Phenethicillin | PM19 | F02 | 266 | 265 | 274 | 288 | 267 | 261 | |
| Phenethicillin | PM19 | F03 | 278 | 273 | 261 | 261 | 286 | 268 | |
| Phenethicillin | PM19 | F04 | 27 | 19 | 26 | 24 | 20 | | |
| Blasticidin S | PM19 | F05 | 298 | 302 | 292 | 289 | 295 | 288 | 0.966 |
| Blasticidin S | PM19 | F06 | 295 | 292 | 289 | 295 | 286 | 291 | |
| Blasticidin S | PM19 | F07 | | 33 | 31 | 39 | | 27 | |
| Blasticidin S | PM19 | F08 | 37 | 25 | 27 | 23 | 41 | 19 | |
| Sodium Caprylate | PM19 | F09 | 301 | 274 | 294 | 304 | 292 | 292 | 0.835 |
| Sodium Caprylate | PM19 | F10 | 290 | 278 | 273 | 282 | 290 | 277 | |
| Sodium Caprylate | PM19 | F11 | | 176 | 136 | 53 | | 77 | |
| Sodium Caprylate | PM19 | F12 | 40 | 20 | 40 | 64 | 23 | 50 | |
| Lauryl sulfobetaine | PM19 | G01 | 306 | 294 | 310 | 325 | 325 | 321 | 0.679 |

| | | | | | | | | | |
|---|---|---|---|---|---|---|---|---|---|
| Lauryl sulfobetaine | PM19 | G02 | 291 | 280 | 292 | 303 | 295 | 288 | |
| Lauryl sulfobetaine | PM19 | G03 | 187 | | 164 | 175 | | 152 | |
| Lauryl sulfobetaine | PM19 | G04 | 227 | 229 | 238 | 250 | 271 | 222 | |
| Dihydrostreptomycin | PM19 | G05 | 301 | 287 | 289 | 292 | 289 | 274 | 0.641 |
| Dihydrostreptomycin | PM19 | G06 | 293 | 288 | 279 | 298 | 296 | 305 | |
| Dihydrostreptomycin | PM19 | G07 | 289 | 294 | 288 | 288 | 287 | 269 | |
| Dihydrostreptomycin | PM19 | G08 | 295 | 284 | 278 | 292 | 300 | 295 | |
| Hydroxylamine | PM19 | G09 | 283 | 277 | 294 | 296 | 290 | 284 | 0.009 |
| Hydroxylamine | PM19 | G10 | 292 | 290 | 281 | 295 | 293 | 291 | |
| Hydroxylamine | PM19 | G11 | 280 | 274 | 279 | 302 | 297 | 297 | |
| Hydroxylamine | PM19 | G12 | 296 | 291 | 303 | 308 | 302 | 294 | |
| Hexaminecobalt (III) Chloride | PM19 | H01 | 281 | 296 | 316 | 293 | 296 | 281 | 0.543 |
| Hexaminecobalt (III) Chloride | PM19 | H02 | 300 | 304 | 311 | 293 | 303 | 284 | |
| Hexaminecobalt (III) Chloride | PM19 | H03 | 298 | 292 | 275 | 305 | 305 | 292 | |
| Hexaminecobalt (III) Chloride | PM19 | H04 | 317 | 298 | 290 | 326 | 317 | 325 | |
| Thioglycerol | PM19 | H05 | 292 | 296 | 283 | 288 | 290 | 274 | 0.607 |
| Thioglycerol | PM19 | H06 | 284 | 300 | 292 | 289 | 306 | 292 | |
| Thioglycerol | PM19 | H07 | 256 | 234 | 252 | 269 | 255 | 281 | |
| Thioglycerol | PM19 | H08 | 261 | 249 | 261 | 264 | 277 | 229 | |
| Polymyxin B | PM19 | H09 | 295 | 285 | 286 | 286 | 285 | 277 | 0.518 |
| Polymyxin B | PM19 | H10 | | 27 | 33 | 290 | 281 | | |
| Polymyxin B | PM19 | H11 | 33 | 32 | 34 | 20 | 19 | 16 | |
| Polymyxin B | PM19 | H12 | 37 | 38 | 47 | 21 | 23 | 25 | |
| Amitriptyline | PM20B | A01 | | 205 | 216 | 304 | 311 | 300 | 0.628 |
| Amitriptyline | PM20B | A02 | 296 | 288 | 289 | 301 | 303 | 298 | |
| Amitriptyline | PM20B | A03 | 320 | 320 | 320 | 310 | 310 | 300 | |
| Amitriptyline | PM20B | A04 | 14 | 13 | 14 | 26 | 30 | 26 | |
| Apramycin | PM20B | A05 | 295 | 289 | 290 | 294 | 299 | 295 | 0.109 |
| Apramycin | PM20B | A06 | 297 | 289 | 294 | 302 | 298 | 292 | |
| Apramycin | PM20B | A07 | 291 | 285 | 288 | 292 | 301 | 291 | |
| Apramycin | PM20B | A08 | 297 | 291 | 290 | 291 | 288 | 288 | |
| Benserazide | PM20B | A09 | 309 | 304 | 306 | 307 | 309 | 306 | 0.899 |
| Benserazide | PM20B | A10 | 322 | 317 | 318 | 329 | 330 | 326 | |
| Benserazide | PM20B | A11 | 176 | 173 | 183 | 171 | 183 | 175 | |
| Benserazide | PM20B | A12 | 231 | 226 | 232 | 226 | 242 | 232 | |
| Orphenadrine | PM20B | B01 | 289 | 282 | 288 | 286 | 285 | 283 | 0.954 |

| | | | | | | | | | |
|---|---|---|---|---|---|---|---|---|---|
| Orphenadrine | PM20B | B02 | 291 | 289 | 289 | 290 | 289 | 282 | |
| Orphenadrine | PM20B | B03 | 109 | | 122 | 128 | 106 | 126 | |
| Orphenadrine | PM20B | B04 | 14 | 14 | 11 | 22 | 29 | 18 | |
| D,L-Propranolol | PM20B | B05 | 288 | 282 | 286 | 289 | 292 | 283 | 0.985 |
| D,L-Propranolol | PM20B | B06 | 305 | 299 | 301 | 309 | 301 | 297 | |
| D,L-Propranolol | PM20B | B07 | | | | | | | |
| D,L-Propranolol | PM20B | B08 | 15 | 17 | 18 | 14 | 19 | 18 | |
| Tetrazolium Violet | PM20B | B09 | 344 | 346 | 344 | 340 | 338 | 337 | 0.935 |
| Tetrazolium Violet | PM20B | B10 | 352 | 350 | 351 | 336 | 339 | 337 | |
| Tetrazolium Violet | PM20B | B11 | 193 | 229 | 206 | 212 | 201 | 195 | |
| Tetrazolium Violet | PM20B | B12 | 53 | 51 | 53 | 60 | 67 | 60 | |
| Thioridazine | PM20B | C01 | 308 | 296 | 299 | 303 | 301 | 297 | 0.031 |
| Thioridazine | PM20B | C02 | 317 | 316 | 316 | 295 | 302 | 294 | |
| Thioridazine | PM20B | C03 | 325 | 305 | 315 | 292 | 318 | 308 | |
| Thioridazine | PM20B | C04 | 290 | 317 | 331 | 312 | 289 | | |
| Atropine | PM20B | C05 | 290 | 280 | 292 | 293 | 291 | 285 | 0.940 |
| Atropine | PM20B | C06 | 286 | 276 | 290 | 297 | 284 | 279 | |
| Atropine | PM20B | C07 | | 152 | 117 | 96 | | 124 | |
| Atropine | PM20B | C08 | 15 | 20 | 16 | 13 | 16 | 11 | |
| Ornidazole | PM20B | C09 | 278 | 278 | 277 | 269 | 276 | 263 | 0.815 |
| Ornidazole | PM20B | C10 | 280 | 278 | 274 | 266 | 261 | 261 | |
| Ornidazole | PM20B | C11 | 294 | 289 | 298 | 276 | 272 | 269 | |
| Ornidazole | PM20B | C12 | | 43 | 38 | | 59 | 52 | |
| Proflavine | PM20B | D01 | 295 | 288 | 293 | 301 | 302 | 294 | 0.607 |
| Proflavine | PM20B | D02 | 294 | 287 | 292 | 283 | 296 | 285 | |
| Proflavine | PM20B | D03 | 305 | 303 | 286 | 306 | 308 | 306 | |
| Proflavine | PM20B | D04 | 308 | 298 | 310 | 293 | 302 | 304 | |
| Ciprofloxacin | PM20B | D05 | 283 | 277 | 279 | 252 | 284 | 280 | 0.095 |
| Ciprofloxacin | PM20B | D06 | 296 | 291 | 287 | 294 | 286 | 283 | |
| Ciprofloxacin | PM20B | D07 | 286 | 289 | 283 | 284 | 281 | 271 | |
| Ciprofloxacin | PM20B | D08 | 286 | 291 | 299 | 292 | 281 | 283 | |
| 18-Crown-6-Ether | PM20B | D09 | 282 | 279 | 279 | 278 | 278 | 273 | 0.715 |
| 18-Crown-6-Ether | PM20B | D10 | 273 | 274 | 281 | 275 | 272 | 271 | |
| 18-Crown-6-Ether | PM20B | D11 | 249 | 250 | 253 | 248 | 246 | 246 | |
| 18-Crown-6-Ether | PM20B | D12 | | 117 | 111 | 129 | 129 | 126 | |
| Crystal Violet | PM20B | E01 | 290 | 290 | 291 | 289 | 288 | 286 | 0.500 |

| | | | | | | | | | |
|---|---|---|---|---|---|---|---|---|---|
| Crystal Violet | PM20B | E02 | 270 | 268 | 272 | 281 | 283 | 273 | |
| Crystal Violet | PM20B | E03 | 293 | 289 | 291 | 298 | 300 | 294 | |
| Crystal Violet | PM20B | E04 | 314 | 315 | 316 | 318 | 323 | 322 | |
| Dodine | PM20B | E05 | 286 | 292 | 276 | 293 | 285 | 283 | 0.963 |
| Dodine | PM20B | E06 | 283 | 287 | 294 | 280 | 285 | 287 | |
| Dodine | PM20B | E07 | 279 | 276 | 277 | 297 | 286 | 270 | |
| Dodine | PM20B | E08 | 16 | 16 | 18 | 23 | 19 | 20 | |
| Hexachlorophene | PM20B | E09 | 274 | 285 | 270 | 274 | 280 | 272 | 0.314 |
| Hexachlorophene | PM20B | E10 | 287 | 283 | 265 | 281 | 276 | 283 | |
| Hexachlorophene | PM20B | E11 | 250 | 272 | 261 | 279 | 277 | 261 | |
| Hexachlorophene | PM20B | E12 | 217 | 221 | 197 | 236 | 222 | 267 | |
| 4-Hydroxycoumarin | PM20B | F01 | 295 | 288 | 293 | 294 | 298 | 295 | 0.594 |
| 4-Hydroxycoumarin | PM20B | F02 | 269 | 267 | 270 | 286 | 291 | 288 | |
| 4-Hydroxycoumarin | PM20B | F03 | 271 | 258 | 267 | 280 | 277 | 275 | |
| 4-Hydroxycoumarin | PM20B | F04 | 66 | 50 | 43 | 49 | 34 | | |
| Oxytetracycline | PM20B | F05 | 278 | 289 | 265 | 286 | 288 | 280 | 0.518 |
| Oxytetracycline | PM20B | F06 | 292 | 297 | 295 | 303 | 296 | 282 | |
| Oxytetracycline | PM20B | F07 | 276 | 286 | 293 | 278 | 282 | 280 | |
| Oxytetracycline | PM20B | F08 | 290 | 292 | 288 | 277 | 276 | 285 | |
| Pridinol | PM20B | F09 | 279 | 286 | 275 | 284 | 293 | 287 | 0.869 |
| Pridinol | PM20B | F10 | 280 | 278 | 283 | 286 | 276 | 267 | |
| Pridinol | PM20B | F11 | | 216 | 255 | 128 | | 124 | |
| Pridinol | PM20B | F12 | 60 | 70 | 54 | 125 | 111 | 81 | |
| Captan | PM20B | G01 | 271 | 269 | 272 | 291 | 290 | 288 | 0.006 |
| Captan | PM20B | G02 | 266 | 264 | 265 | 277 | 297 | 281 | |
| Captan | PM20B | G03 | 283 | 289 | 275 | 300 | 297 | 298 | |
| Captan | PM20B | G04 | 300 | 296 | 300 | 294 | 300 | 310 | |
| 3,5-Dinitrobenzene | PM20B | G05 | 290 | 289 | 285 | 290 | 292 | 286 | 0.995 |
| 3,5-Dinitrobenzene | PM20B | G06 | 306 | 297 | 303 | 314 | 312 | 305 | |
| 3,5-Dinitrobenzene | PM20B | G07 | 299 | 301 | 321 | 302 | 310 | 292 | |
| 3,5-Dinitrobenzene | PM20B | G08 | 24 | 21 | 20 | 18 | 15 | 16 | |
| 8-Hydroxyquinoline | PM20B | G09 | 282 | 291 | 274 | 300 | 289 | 290 | 0.956 |
| 8-Hydroxyquinoline | PM20B | G10 | 274 | 268 | 276 | 270 | 275 | 268 | |
| 8-Hydroxyquinoline | PM20B | G11 | 203 | | | | | | |
| 8-Hydroxyquinoline | PM20B | G12 | 29 | 28 | 32 | 31 | 40 | 26 | |
| Patulin | PM20B | H01 | 297 | 285 | 299 | 290 | 287 | 286 | 0.985 |

| | | | | | | | | | |
|---|---|---|---|---|---|---|---|---|---|
| Patulin | PM20B | H02 | 28 | | | | | | |
| Patulin | PM20B | H03 | 27 | 24 | 27 | 12 | 14 | 16 | |
| Patulin | PM20B | H04 | 41 | 33 | 40 | 33 | 28 | 35 | |
| Tolylfluanid | PM20B | H05 | 290 | 280 | 282 | 287 | 287 | 279 | 0.812 |
| Tolylfluanid | PM20B | H06 | 290 | 280 | 290 | 293 | 286 | 289 | |
| Tolylfluanid | PM20B | H07 | 287 | 284 | 286 | 282 | 291 | 278 | |
| Tolylfluanid | PM20B | H08 | 287 | 289 | 294 | 298 | 290 | 285 | |
| Troleandomycin | PM20B | H09 | 290 | 279 | 283 | 277 | 293 | 281 | 0.120 |
| Troleandomycin | PM20B | H10 | 293 | 292 | 286 | 272 | 277 | 262 | |
| Troleandomycin | PM20B | H11 | 281 | 277 | 280 | 275 | 281 | 277 | |
| Troleandomycin | PM20B | H12 | 275 | 248 | | | 137 | 138 | |
