# Supplementary material for: Membrane properties modulation by SanA: implications for xenobiotic resistance in Salmonella Typhimurium
Source: Front Microbiol. 2024 Jan 5;14:1340143. doi: 10.3389/fmicb.2023.1340143 (PMC10797042; doi:10.3389/fmicb.2023.1340143)
Supplement: Supplementary file 2 [file Data_Sheet_2.docx]

**Supplementary material**


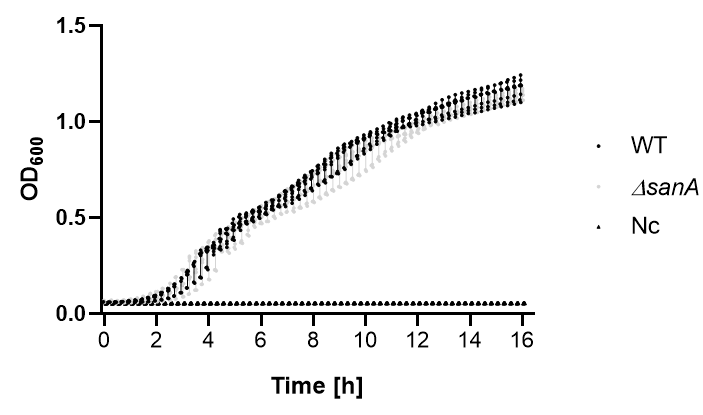


**Fig. S1** **Growth curves of *S*. Typhimurium 4/74**

Growth curves of *S*. Typhimurium 4/74 and its deletion mutant *ΔsanA* in LB medium. Optical density (OD_600_) was measured 16 h in 15 min intervals at 37°C. Data shown are means and SEM for at least three independent experiments.

**Fig. S2** **Growth curve of *S*. Typhimurium 4/74 transformed with empty pWSK29 plasmid or vector with *sanA*** **in the presence of vancomycin or bile salts**

Growth curve of *S*. Typhimurium 4/74 transformed with empty pWSK29 plasmid or vector with *sanA* in the presence of: **A)** vancomycin in the concentration 62.5 and 125 µg/ml, **B)** bile salts in the concentration 0.94 and 1.88 %. Optical density (OD_600_) was measured 16 h in 15 min intervals at 37°C. Data shown are means and SEM for at least three independent experiments.

**Fig. S3** **Growth of *S*. Typhimurium 4/74 and its deletion mutant *ΔsanA* in MHB medium in the presence of different agents**

Optical density (OD_600_) was measured after 16 h incubation at 37°C. Data shown are means and SEM for at least three independent experiments. Statistical significance was determined by two-way ANOVA with Tukey’s correction (*, p<0.05; **, p <0.01; ***, p<0.001). Concentrations with statistically significant differences indicated with asterix were chosen to the further analysis.

**A) B)**


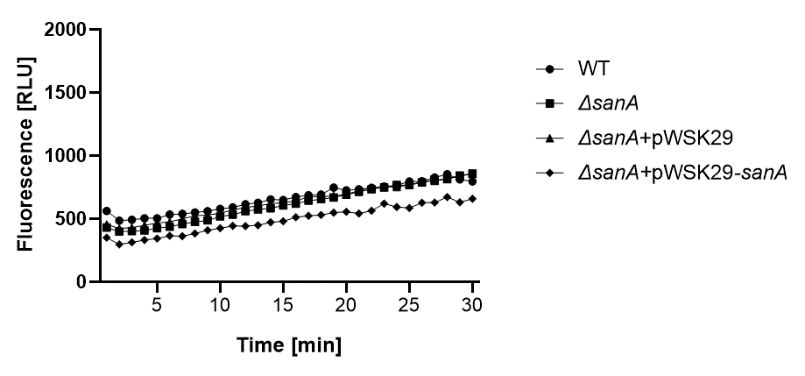

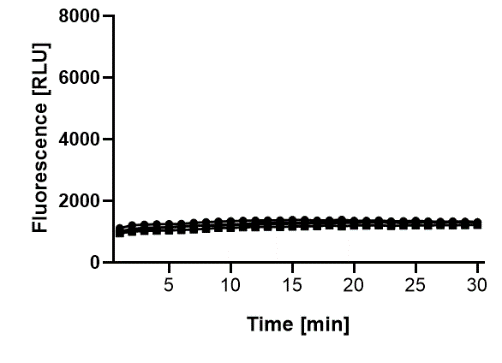


**Fig. S4** **Outer membrane permeability of *S*. Typhimurium 4/74**

Outer membrane permeability of *S*. Typhimurium 4/74, its deletion mutant *ΔsanA* and *ΔsanA* transformed with empty pWSK29 plasmid or vector with *sanA* **A)** cationic dye ethidium bromide uptake **B)** neutral dye Nile red uptake. The assay was conducted in the absence of CCCP to measure a passive dyes uptake with the interference of efflux mechanisms. Data shown are representative of at least three independent experiments with similar results.

**
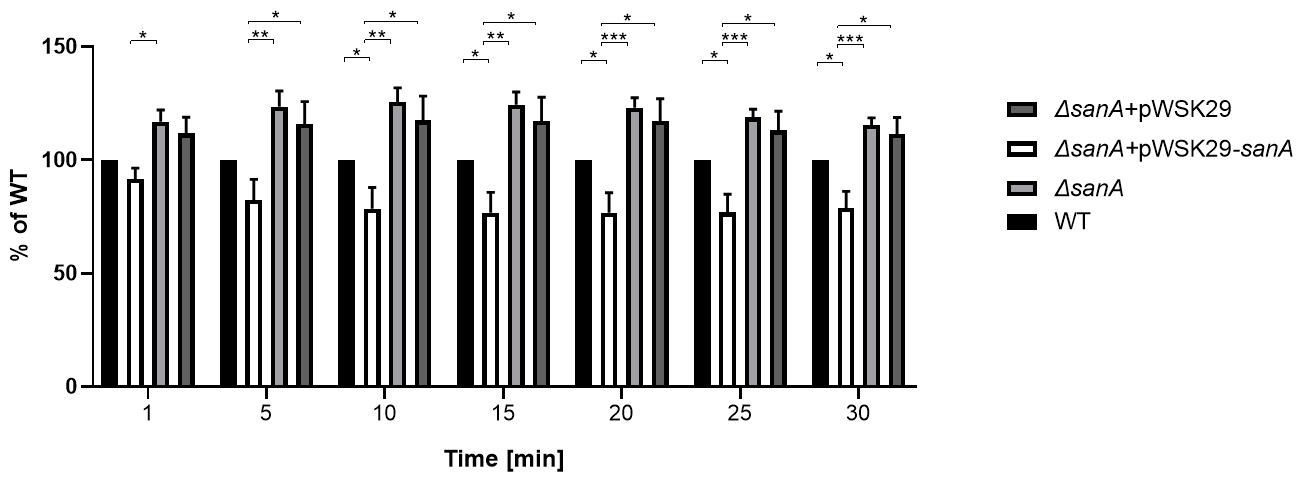
A)**

**
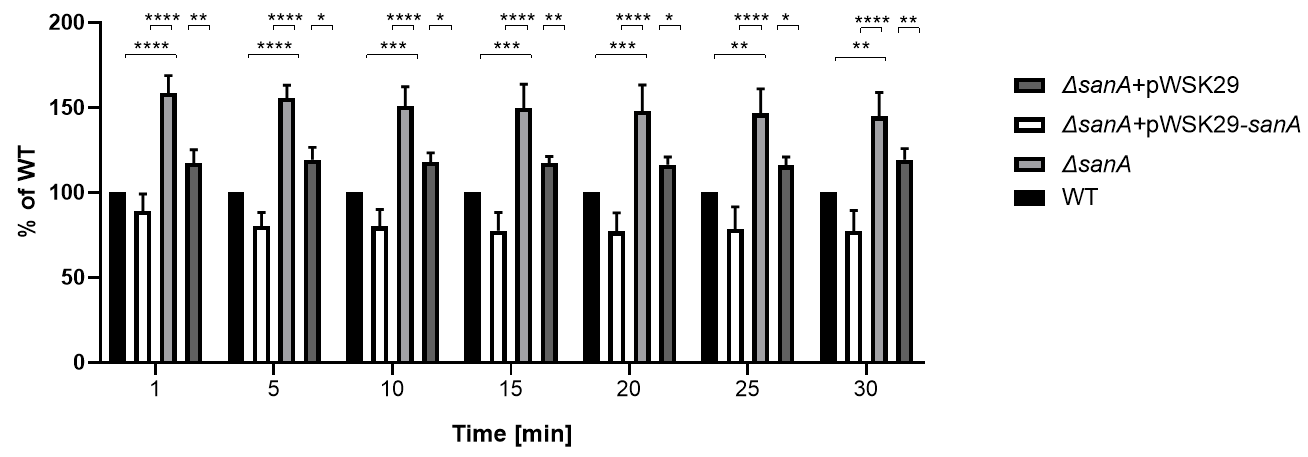
B)**

**Fig. S5** Outer membrane permeability of *S*. Typhimurium 4/74 deletion mutant *ΔsanA* transformed with empty pWSK29 plasmid or vector with *sanA* wild type version; *ΔsanA*, and WT **A)** cationic dye ethidium bromide uptake **B)** neutral dye Nile red uptake. The assays were conducted in the presence of CCCP to prevent the efflux of compound by active pump to measure only passive permeability. Data are represented as the percent of isolate’s permeability relative to WT. Data shown are means and SEM for at least three independent experiments. Statistical significance was determined by two-way ANOVA with Tukey’s correction (*, p<0.05; **, p <0.01; ***, p<0.001; ****, p<0.0001).
